# Cyclohexene‐Embedded Dicyanomethylene Merocyanines – Consecutive Three‐Component Coupling‐Addition Synthesis and Chromophore Characteristics

**DOI:** 10.1002/open.202300128

**Published:** 2023-09-15

**Authors:** Julian Papadopoulos, Guido J. Reiss, Bernhard Mayer, Thomas J. J. Müller

**Affiliations:** ^1^ Institut für Organische Chemie und Makromolekulare Chemie Heinrich-Heine-Universität Düsseldorf Universitätsstraße 1 40225 Düsseldorf Germany; ^2^ Institut für Anorganische Chemie und Strukturchemie Heinrich-Heine-Universität Düsseldorf Universitätsstraße 1 40225 Düsseldorf Germany

**Keywords:** cross-coupling, DFT calculations, merocyanines, Michael addition, multicomponent reactions

## Abstract

A concise and efficient consecutive three‐component alkynylation‐addition synthesis of cyclohexene‐embedded dicyanomethylene merocyanines furnishes a small library of dyes in moderate to excellent yield. The dyes possess strong absorption coefficients of the longest wavelength absorption bands. According to the crystal structure, the small bond length alternations account for a highly delocalized electronic ground state. The electronic structure of the absorption bands is qualitatively rationalized by TDDFT calculations, which explain that intense HOMO‐LUMO transitions along the merocyanine axis lead to cyanine similar Stokes shifts.

## Graphical abstract

A library of cyclohexene‐embedded dicyanomethylene merocyanines is efficiently obtained by a consecutive three‐component alkynylation‐addition synthesis in moderate to excellent yield. The dominant intense longest wavelength absorption bands can be assigned, by TDDFT calculations, as HOMO‐LUMO transitions along the merocyanine axis.

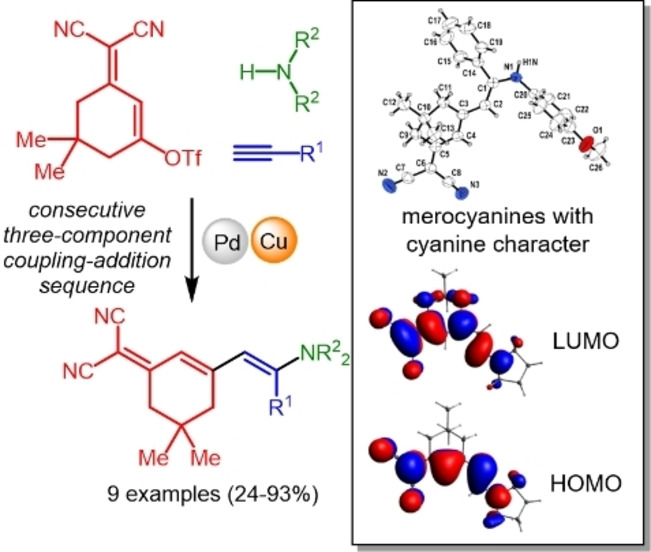

## Introduction

Multicomponent reactions (MCR) combine efficiency and efficacy as one‐pot processes, where more than three compounds react to give a single product in a rather atom‐economical fashion.^[1]^ As a reactivity‐based concept,^[1c]^ MCRs have ever since enabled catenating various elementary steps, thereby even reaching to transition‐metal catalyzed heterocycle syntheses^[2]^ and one‐pot syntheses employing photo‐ and electrochemistry.^[3]^ By virtue of covering a vast structural and functional space, MCRs have considerably enriched interdisciplinary research in medicinal and natural product chemistry as well as biological chemistry.^[4]^ This diversity‐oriented synthetic concept also has been expanded to functional chromophores,^[5]^ that is, π‐systems that can also be electroactive or luminescent. Among functional chromophores, merocyanines^[6]^ are particularly interesting. They are neutral polymethines and highly polarizable π‐electron systems with intensive longest wavelength absorption bands. As a consequence, they find application in optoelectronics,^[7]^ organic semiconductors,^[8]^ and photovoltaics,^[9]^ and for self‐assembly in solution to nanoscale objects and supramolecular materials.^[10]^ While merocyanines and many polymethine dyes are classically synthesized by conventional aldol or Knoevenagel condensations,[^6^, ^11^] MCR processes founded on the catalytic generation of alkynoyl intermediates^[12]^ have become a valuable tool to access fluorophores in a modular one‐pot fashion.[^5c^, ^13^] In recent years, we have successfully employed a concise consecutive three‐component alkynylation‐addition sequence to access of luminescent merocyanines,^[14]^ which also could be embedded as covalently bound constituents in highly fluorescent PMMA copolymers.^[15]^ Moreover, we could establish coumarin based luminophores with tunable electronic properties both on the stage of coumarin merocyanines^[14b]^ and their alkynyl coumarin intermediates.^[16]^ Just very recently, cyclohexene‐embedded merocyanines and cyanines were established by consecutive coupling‐addition synthesis^[17]^ for taking advantage of rigidization of the merocyanines, similarly as previously reported by the Laschat group.^[18]^ Here, we report the extension of the alkynylation‐addition merocyanine formation with a dicyanomethylene expanded acceptor unit and the investigation of their electronic properties.

## Results and Discussion

### Synthesis and Structure

**Scheme 1:**
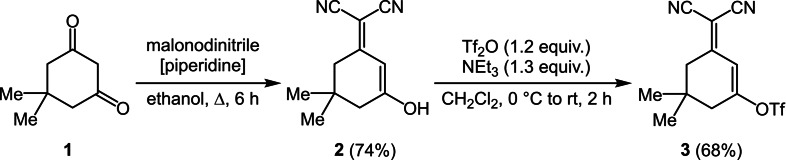
Synthesis of triflate **3** by Knoevenagel condensation of dimedone (**1**) and malononitrile and triflation.

Starting from dimedone (**1**), that is, 5,5‐dimethylcyclohexane‐1,3‐dione, is reacted by Knoevenagel condensation with malonodinitrile in boiling ethanol in presence of catalytic amounts of piperidine as a base to give the condensation product (**2**) in 74 % yield (Scheme 1).^[19]^ From this product, by reaction with triflic anhydride, the corresponding triflate **3** is formed according to the literature in 68 % yield.^[20]^

In contrast to the previously published standard protocol of the coupling‐addition synthesis of cyclohexene‐embedded merocyanines,^[17]^ in a model reaction, only a low yield of the desired dicyanomethylene merocyanine was obtained. Indeed, the initial Sonogashira step turned out to be the limiting step (see Supporting Information). A short optimization study with respect to the palladium catalyst and the employed auxiliary base turned out that of the consecutive three‐component synthesis PdCl_2_(PPh_3_)_2_ and diisopropyl ethylamine (DIPEA) gives almost quantitative yield after isolation. The terminal Michael addition step did not require optimization and proceeded rapidly upon dielectric heating (100 °C) for 1 h.

**Scheme 2:**
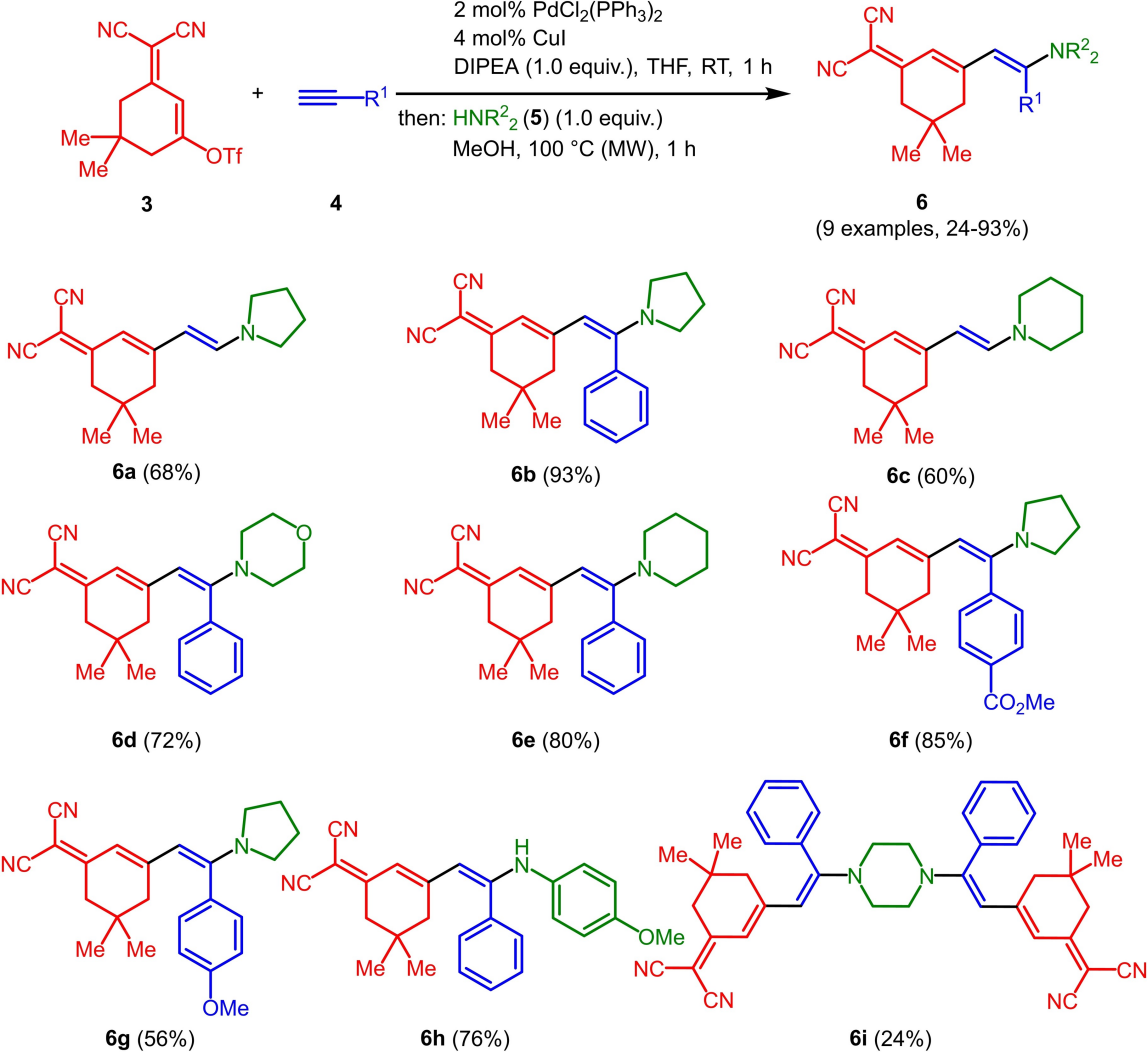
Three‐component alkynylation‐addition synthesis of merocyanines **6**.

With the optimized conditions in hand, triflate **3**, alkynes **4** and amines **5** are reacted in a consecutive one‐pot process to give 9 different merocyanines **6** in moderate to excellent yield (Scheme 2).

**Scheme 3:**
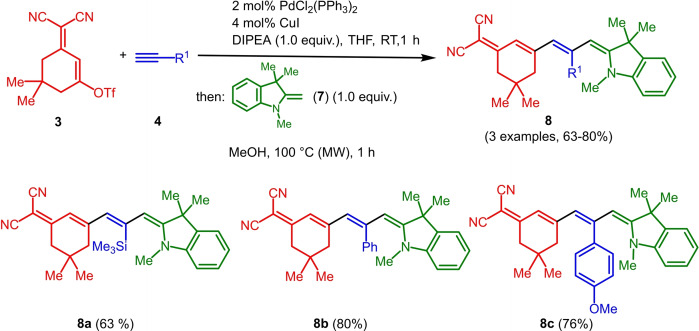
Three‐component alkynylation‐addition synthesis of merocyanines **8**.

**Figure 1:**
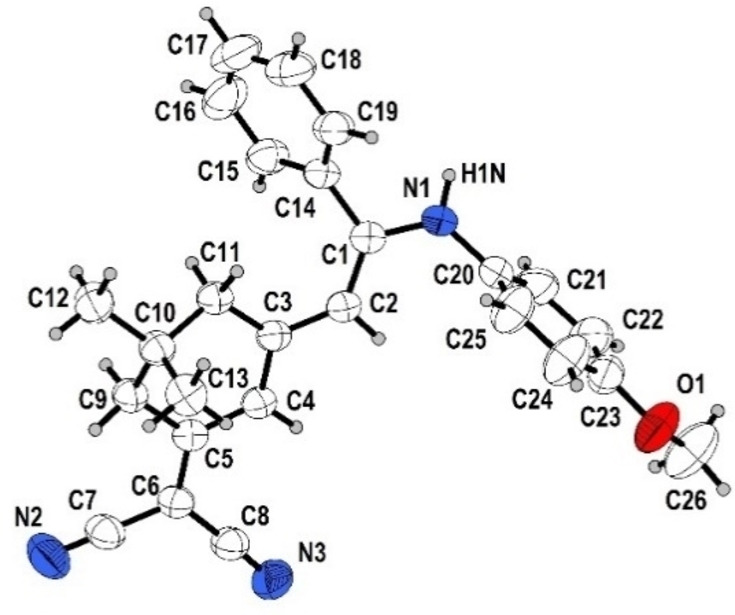
ORTEP plot (50 % probability) of merocyanine **6 h**.

**Figure 2:**
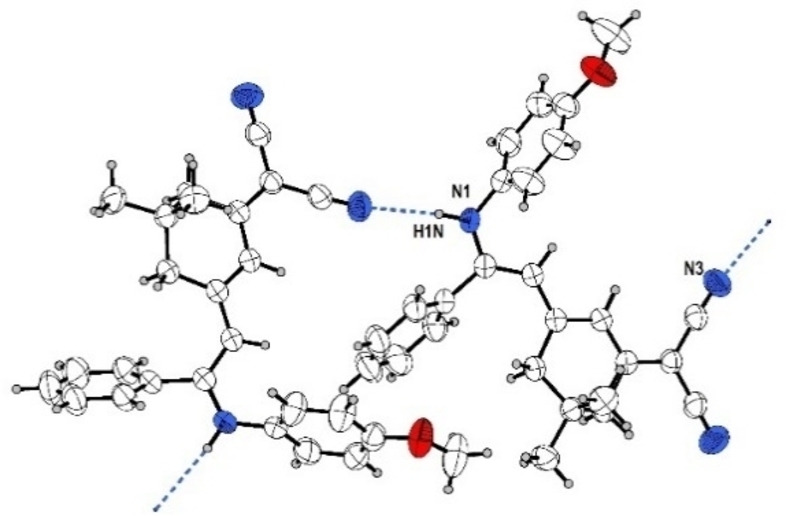
Formation of hydrogen bonded zig‐zag chains of merocyanine **6 h** in the single crystal.

As seen before in MCR merocyanine syntheses^[14a]^ for (TMS)acetylene (**4 a**) the TMS group is cleaved concomitantly with the Michael addition of the amine. The scope of aromatic acetylenes ranges from electron‐deficient (R^1^=*p*‐MeO_2_CC_6_H_4_) over electroneutral (R^1^=C_6_H_5_) to electron‐rich (R^1^=*p*‐MeOC_6_H_4_). Secondary heterocyclic amines, such as morpholine, piperidine and pyrrolidine, as well as the primary amine *p*‐anisidine are the amine nucleophiles **5**, which then constitute the merocyanines’ donor moieties. As a showcase for twofold Michael addition, piperazine as a substrate furnishes the bismerocyanine **6 i**. Likewise, vinyloguous merocyanines **8** with expanded π‐conjugation are obtained by one‐pot coupling addition sequence of triflate **3**, alkynes **4** and Fischer's base (**7**) in good yield (Scheme 3). The structures of the merocyanines **6** and **8** are unambiguously supported by NMR spectroscopy, MS spectrometry and/or combustion analyses. Broad proton signals for the alkyl protons adjacent to the nitrogen atoms indicate a coalescence and therefore dynamic phenomena (vide infra). The preferred ground state configuration of the enamino double bond is *E* according to coupling constants and/or cross‐peaks in the NOESY between the corresponding spatially close protons. In addition, the connectivity and stereochemistry were later corroborated by a crystal structure analysis of merocyanine **6 h** (Figures 1 and 2; see also Table S3, Supporting Information).^[21]^

The inspection of the bond lengths of merocyanine **6 h** (see Supporting Information, Table S4) allows for a calculation of the bond length alternation.^[22]^ With 1.4189 Å, the average CC single bond length in merocyanine **6 h** is shortened and the double bond length is lengthened with 1.3793 Å. This accounts to a bond length alternation of 0.04 Å. Small bond lengths alternations are typical for dicyanomethylene‐type merocyanines.^[22a]^ However, in contrast to typical stacking, for merocyanine **6 h** weak intermolecular hydrogen bonds between the amino groups and the cyano groups form infinite zig‐zag chains in the crystal lattice (Figure 2).

**Figure 3:**
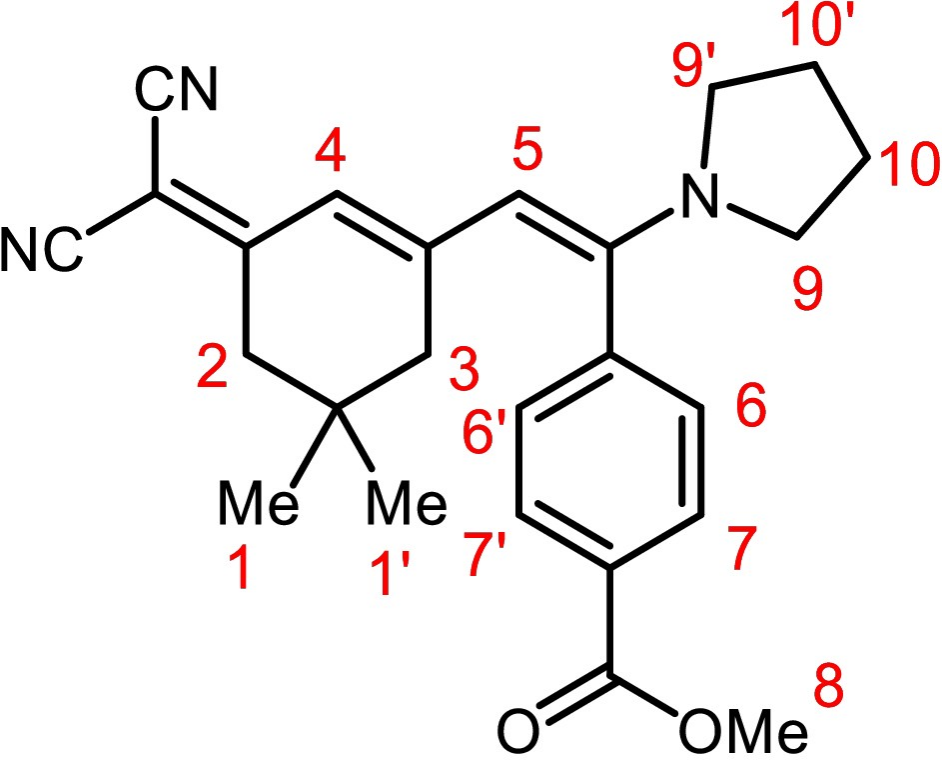
Locant set of the protons of merocyanine **6 f**.

**Figure 4:**
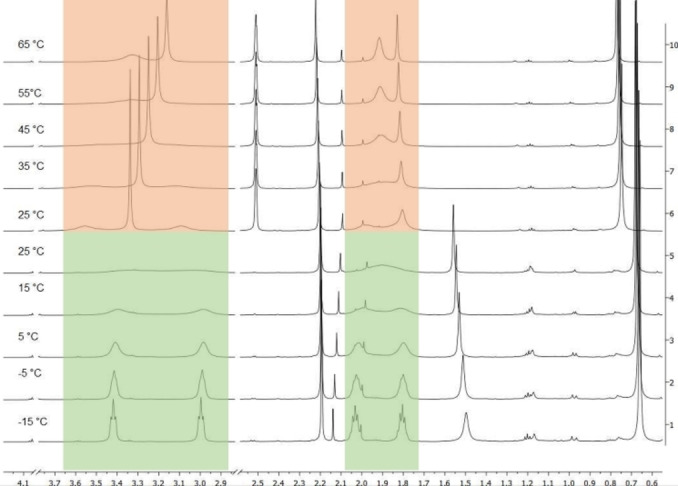
Sections of VT‐^1^H NMR spectra at temperatures from −15 to 65 °C (green: CDCl_3_, orange: DMSO‐d_6_, 600 MHz) with coalescence of the pyrrolidine signals of merocyanine **6 f**.

The occurrence of broad signals for the α‐protons of the dialkylamino moieties of the pyrrolidinyl, piperidinyl and morpholinyl substituents prompted us to carefully study the dynamic behavior by VT (variable temperature) proton NMR spectroscopy. In detail the results from VT NMR spectroscopy of merocyanine **6 f** are exemplarily discussed (Figures 3 and 4). Due to the volatility of CDCl_3_ and the melting point of DMSO‐d_6_,measurements below 25 °C were recorded in CDCl_3_ (green marked area for the coalescent signals) and above 25 °C in DMSO‐d_6_ (orange marked area for the coalescent signals) (Figure 4).

The occurrence of single signals of the chemically and magnetically equivalent methyl groups (H^1^) at *δ* 0.74 with an integral of 6 protons below the coalescence temperature (below room temperature) already indicates that no *E*/*Z* double bond isomerism is relevant in this temperature regime. However, above this temperature the double bond configuration cannot be assigned unambiguously. The NOESY spectrum shows the expected cross‐peaks between the methine signal H^5^ (*δ* 5.45) and H^9,9’^ (*δ* 3.08 and 3.55 or *δ* 3.32, respectively) and methine signal H^4^ (*δ* 5.69) and H^6,6’^ (*δ* 7.47) that unambiguously assign the *E*‐configuration of the enamino moiety. Indeed, the relevant changes in the VT NMR spectra can be seen by resolution at lower temperatures in the section of the spectra where the nonisochronous pyrrolidine signals H^9,9’^ (*δ* 3.08 and 3.55) and H^10,10’^ (*δ* 1.98 and 1.79) appear, which can be identified by the coupling pattern (Figure 4). The coalescence of the pyrrolidinyl protons H^9,9’^ and H^10,10’^ that causes coinciding to isochronous broad signals at *δ* 3.32 and *δ* 1.89, respectively. This can be interpreted as a freely rotating pyrrolidinyl substituent overcoming the rotational barrier imposed by the push‐pull system of the merocyanine.

From the VT ^1^H NMR spectra the rate constant $k_{T_{c}}$ and, thereby, the Gibbs free enthalpy of activation for rotation about the C−N bond can be calculated to Δ*G_c_* ^*ǂ*^=61 kJ/mol (for details, see Supporting Information, Chpt. 8).^[23]^ The rotational barrier of merocyanine **6 f** lies in the same regime as previously determined barriers of ethyl β‐pyrrolidino 3‐aryl enoates, however, due to the expanded π‐electron conjugation by roughly 20 kJ/mol lower than those of β‐nitroenamines.^[24]^ Furthermore, at *T_c_* =−5 °C (268.15 K) a splitting into a coupling pattern occurs which can be identified by triplet coupling constants at −15 °C at *δ* 1.84 (^*3*^ *J*=6.8 Hz), 2.07 (^*3*^ *J*=6.9 Hz), 3.04 (^*3*^ *J*=6.8 Hz) and 3.46 (^*3*^ *J*=6.9 Hz.). This splitting accounts for the inversion of the pyrrolidine ring and its barrier Δ*G_c_* ^*ǂ*^ of approximately 51 kJ/mol can be calculated from the corresponding coalescence temperature *T_c_* =−5 °C,^[23]^ the signal frequency differences and the geminal *J* ^*3*^ coupling constants.

### Photophysical Properties

**Figure 5:**
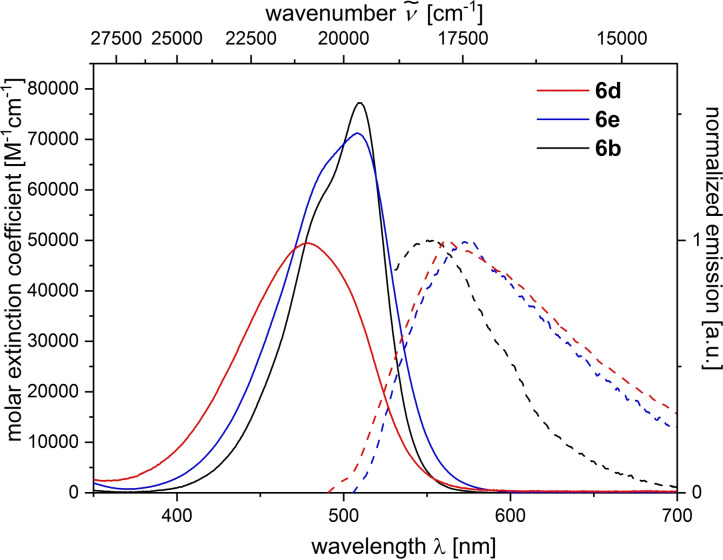
Absorption (solid line) and emission (dashed line) spectra of merocyanines **6 b**, **6 d**, **6 e** (recorded in dichloromethane at *T*=293 K).

Merocyanines **6** and **8** are red to violet solids and their solutions are intensively colored, yet, they are only weakly luminescent. Therefore, they were investigated by UV/Vis and emission spectroscopy in dichloromethane solution and show broad unstructured absorption and emission bands (Figure 5, Table 1). Absorption solvatochromism was checked by recording qualitative absorption spectra of compound **6 h** in dichloromethane (493 nm) and acetonitrile (496 nm). This minute positive absorption solvatochromism was therefore not considered for further investigation. The low quantum yield (*Φ_f_* =0.01) was only determined as a proxy for merocyanine **6 a** with coumarin 153 as a standard^[25]^ (Table 1, entry 1). The longest wavelength absorption maximum *λ*_max,abs_ of compounds **6 a**–**i** is found between 479 to 513 nm with molar extinction coefficients *ϵ* between 42100 and 98000 m ^−1^ cm^−1^. The emission maxima *λ*_max,em_ are in the range of 512–623 nm (Table 1, entries 1–9). Merocyanines **8** contain the more expanded π‐electron conjugation and therefore the absorption maxima appear redshifted between 537 and 549 nm (Table 1, entries 10–12). Simultaneously, the steric interactions between the substituents towards the donor end tend to enhance deviation from coplanarity as seen from significantly lower absorption coefficients. The emission maxima are even more bathochromically shifted than those of merocyanines **6 a**–**6 i** and are found between 633 and 695 nm, however, very weak in intensity.

**Table 1:** Selected photophysical data of merocyanines **6** and **8** (recorded in CH_2_Cl_2_ at *T*=293 K).

entry	compound	*λ*_max,abs_ [nm] (*ϵ* [m ^−1^ cm^−1^])^[a]^	*λ*_max,em_ [nm] (*Φ_F_*)^[b]^	Stokes shift Δ$\tilde{\upsilon }$ [cm^−1^]^c^
1	**6 a**	493 (98000)	513 (<0.01)	800
2	**6 b**	510 (77700)	550	1400
3	**6 c**	490 (72600)	512	860
4	**6 d**	479 (46600)	562	3100
5	**6 e**	508 (71700)	576	2300
6	**6 f**	507 (46000)	570	2200
7	**6 g**	513 (56800)	559	1600
8	**6 h**	493 (42100)	623	4200
9	**6 i**	498 (71100)	576	2700
10	**8 a**	549 (32600)	695	3800
11	**8 b**	537 (21400)	633	2800
12	**8 c**	539 (27700)	647	3100

[a] Longest wavelength absorption maximum. [b] Fluorescence quantum yield *Φ_F_* is determined with coumarin 153 as a standard in ethanol UVASOL® *Φ_F_=*0.55 at λ_*exc*_=420 nm.^[25]^ [c] Δ$\tilde{\upsilon }$ =1/*λ*_*max,abs*_−1/*λ*_*max,em*_ [cm^−1^].

Structure‐property relationships can be identified in comparing consanguineous series. In the first comparison merocyanines **6 a** and **6 c** which are void of a substituent R^2^ show that the stronger donor pyrrolidine (**6 a**) not only causes a redshift of the longest wavelength maximum to 493 nm in comparison to the piperidinyl derivative **6 c**, but also a clear hyperchromic shift of the intensity (Table 1, entries 1 and 3). The emission maxima, however, are very similar. In the second comparison merocyanines **6 b**, **6 d**, and **6 e** bear with the phenyl moiety the same substituent R^2^, however, variable amino donors (Table 1, entries 2, 4, and 5). The longest wavelength absorption maxima blueshift from merocyanine **6 b** (510 nm) over derivative **6 b** (508 nm) to compound **6 e** (479 nm) reflecting the degression of the donor strength in the order from pyrrolidinyl over piperidinyl to morpholinyl, which is likewise seen in the extinction coefficients. Surprisingly, the most redshifted emission maximum at 576 nm is seen for the piperidinyl merocyanine **6 e**. The third comparison finally shows the effect of the absence (merocyanine **6 a**) and presence of aromatic substituents R^2^ (merocyanines **6 b**, **6 f**, **6 g**) and the *p*‐substituent effect of the aryl moiety (Table 1, entries 1, 2, 6, and 7). The aryl substituents R^2^ causes clear redshifts of the lowest energy absorption bands in comparison to the unsubstituted dye **6 a**, however, on the expense of the absorption coefficient, which can be attributed to steric distortion from coplanarity by the presence of the aryl moiety. However, in the excited state the emission is significantly redshifted for the aryl substituted merocyanines revealing an intriguing substituent effect. The most electroneutral phenyl derivative **6 b** possess the most blue‐shifted emission maximum (550 nm) in comparison to the donor‐type anisyl derivative **6 g** (559 nm) and the acceptor type benzoate merocyanine **6 f** (570 nm). This discontinuous behavior resembles the substituent effects previously seen in the coumarin merocyanine series.^[17]^ All R^2^ substituted merocyanines **6** display distinctly large Stokes shifts in comparison to unsubstituted dyes **6 a** and **6 c** as a consequence of larger conformational changes between S_0_ ground state and vibrationally relaxed S_1_ state. Also, large Stokes shifts are found for the merocyanines **8**. However, the aryl substituents conformational and rotational degrees of freedom might open nonradiative deactivation pathways.

### Calculated Electronic Structure

For assigning the photophysical characteristics of merocyanines **6**, calculations of the UV/Vis transitions of geometry optimized structures of merocyanines **6 a**, **6 b**, **6 f**, and **6 g** were performed on the DFT and TDDFT level of theory using various exchange correlation hybrid functionals (B3LYP,^[26]^ LC‐ωB97XD,^[27]^ and LC‐ωHPBE^[28]^) and the 6‐311++G** basis set,^[29]^ which was chosen to most precisely describe polarization as well as long‐range interactions. The long‐range corrected (LC) hybrid density functional affects the solvation (polarizability and the dipole moment) of the ground state energy and of the wavefunctions. The longest wavelength bands are calculated as corrected Linear Response (cLR, S_1_ specific solvation)^[30]^ and as Linear Response (LRS0 solvated). Besides gas phase calculations the polarizable continuum model (PCM)^[31]^ for the dichloromethane as a dielectric continuum is employed as implemented in the program package Gaussian 16.^[32]^ The comparative calculations of the S_1_ states are summarized in (Table 2).

**Table 2:** Experimentally determined (recorded in dichloromethane at *T*=298 K) and TDDFT calculated longest wavelength absorption bands of merocyanines **6 a, 6 b, 6 f**, and **6 g** using various functionals (B3LYP, LC‐ωB97XD, LC‐ωHPBE) with cLR (corrected linear response) and LR (linear response) and 6‐311++G** as a basis set, oscillator strengths *f* and deviations Δ*E*_calcd‐exp_.

	functional	cLR λ_max,abs_ [nm] ([eV])	LR λ_max,abs_ [nm] ([eV])	transition	oscillator strength *f*	Δ*E*_calcd–exp_^[a]^
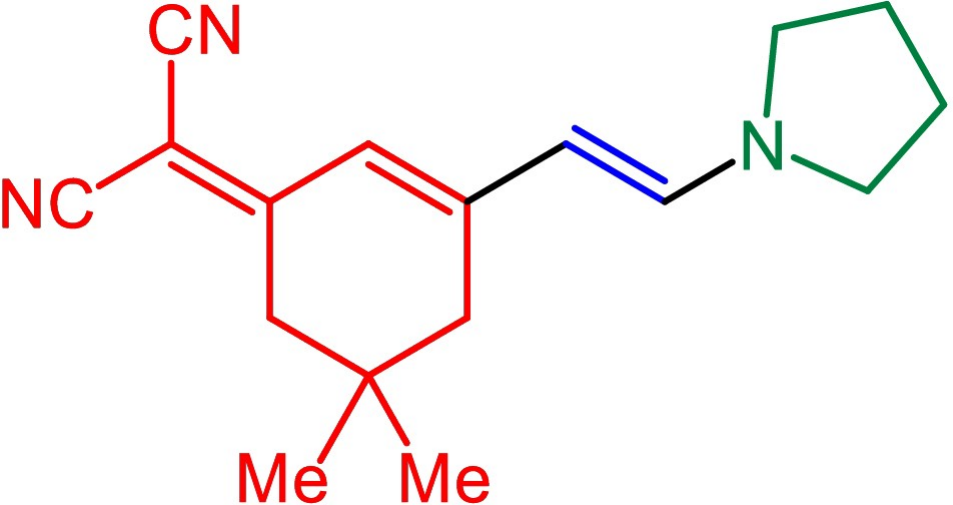	B3LYP	395.6 (3.134)	422.2 (2.937)	HOMO→LUMO (100 %)	1.3829	0.422
**6 a** λ_max,abs exp_ 493 nm/2.515 eV (98000 m ^−1^ cm^−1^)	LC‐ωB97XD	384.4 (3.226)	409.2 (3.030)	HOMO→LUMO (97.0 %)	1.4112	0.515
	LC‐ωHPBE	378.0 (3.280)	400.4 (3.097)	HOMO→LUMO (94.9 %)	1.424	0.582
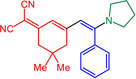	B3LYP	405.5 (3.057)	431.3 (2.875)	HOMO→LUMO (100 %)	1.3634	0.444
**6 b** λ_max,abs exp_ 510 nm/2.431 eV (77700 m ^−1^ cm^−1^)	LC‐ ωB97XD	399.6 (3.102)	423.9 (2.925)	HOMO→ LUMO (96.6 %)	1.3527	0.494
	LC‐ωHPBE	388.4 (3.193)	410.2 (3.023)	HOMO→LUMO (94.9 %)	1.4075	0.592
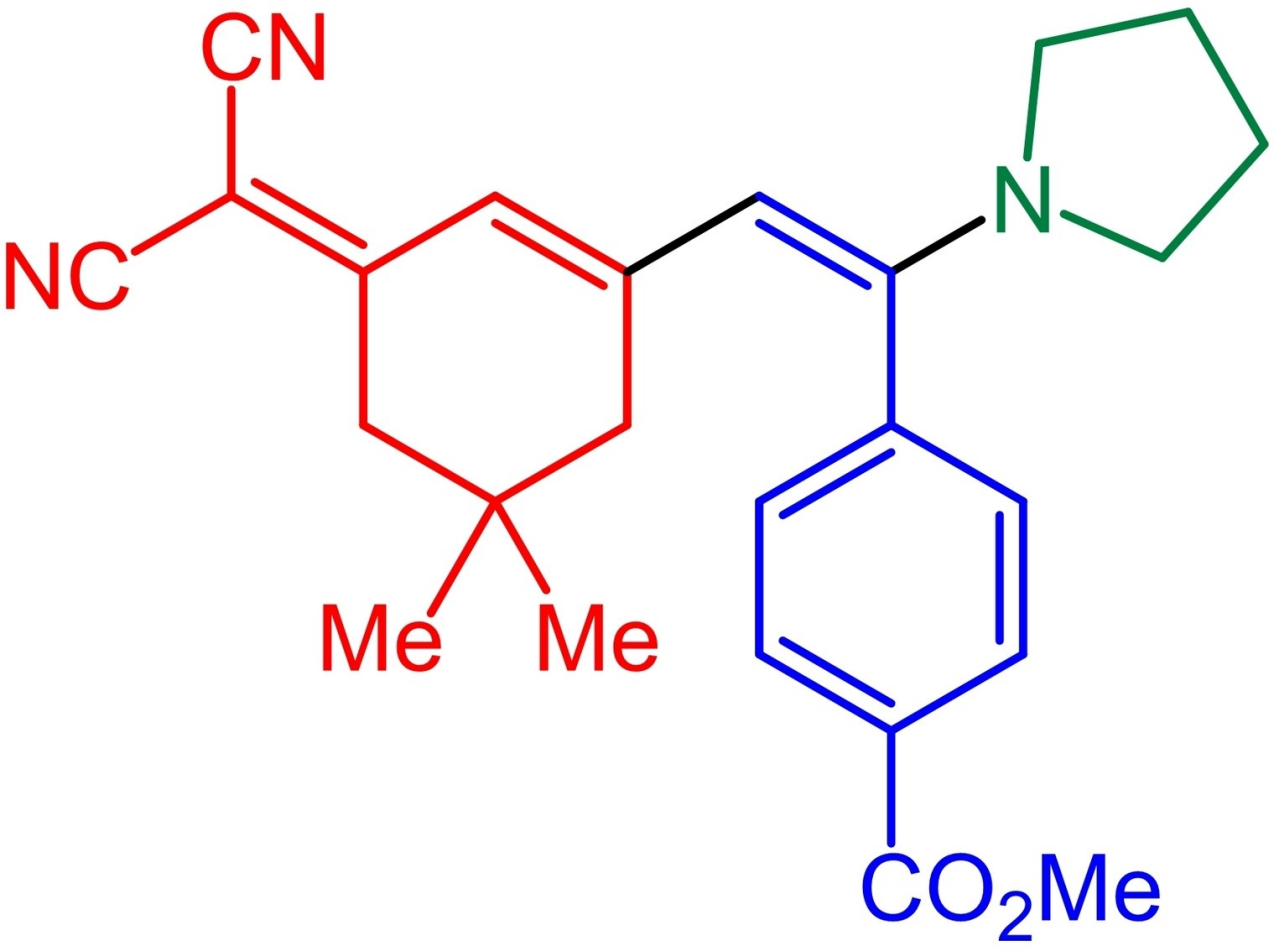	B3LYP	424.4 (2.921)	431.5 (2.873)	HOMO→LUMO (99.9 %)	1.3775	0.428
**6 f** λ_max,abs exp_ 507 nm/2.445 eV (46000 m ^−1^ cm^−1^)	LC‐ ωB97XD	395.0 (3.139)	418.6 (2.962)	HOMO→LUMO (96.7 %	1.4015	0.517
	LC‐ωHPBE	387.0 (3.204)	407.8 (3.040)	HOMO→LUMO (94.8 %)	1.4231	0.595
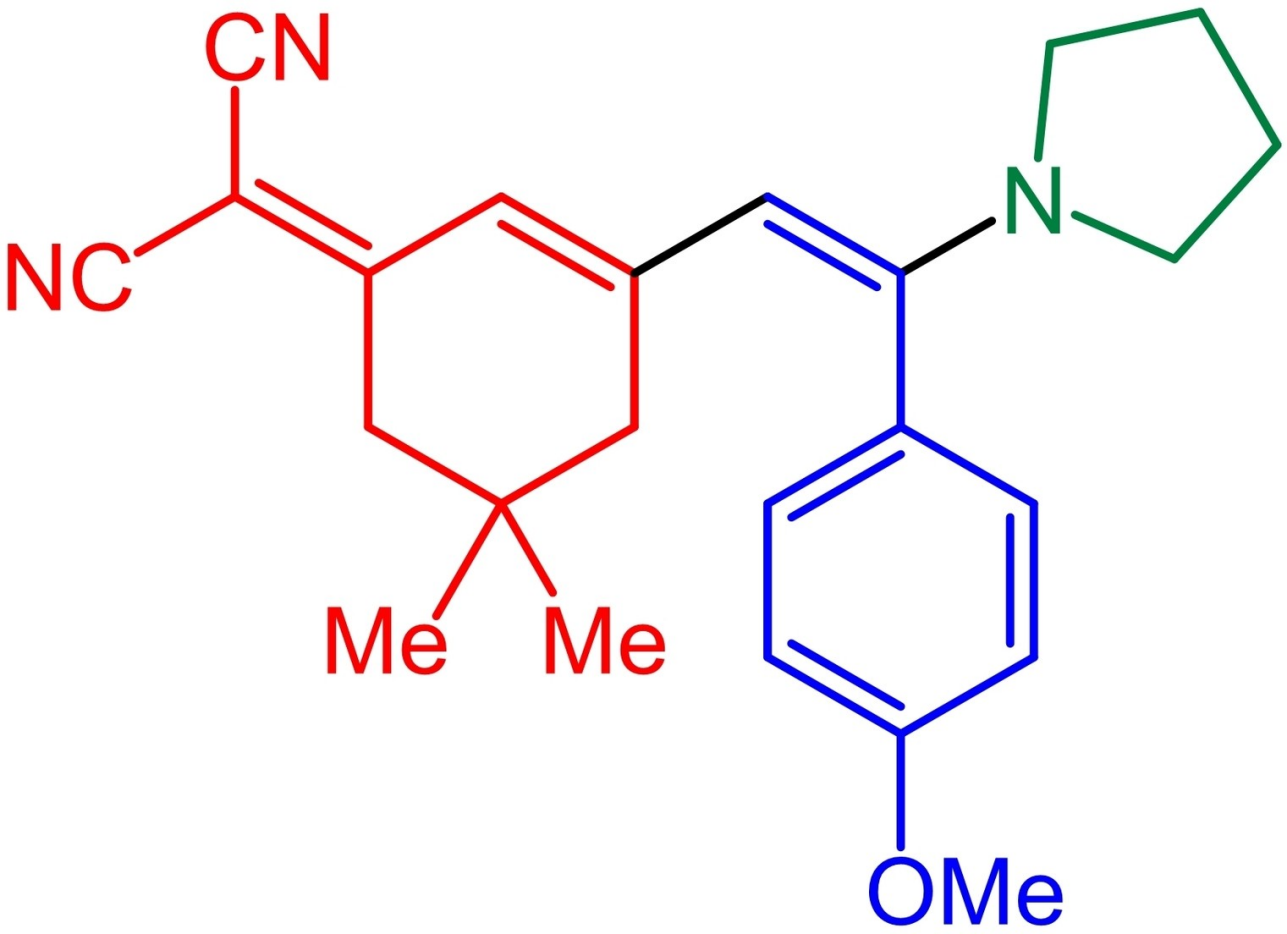	B3LYP	406.5 (3.050)	432.3 (2.868)	HOMO→LUMO (100 %)	1.3678	0.451
**6 g** λ_max,abs exp_ 513 nm/2.417 eV (56800 m ^−1^ cm^−1^)	LC‐ ωB97XD	396.2 (3.129)	420.4 (2.949)	HOMO→LUMO (96.8 %)	1.3891	0.532
	LC‐ωHPBE	389.8 (3.181)	411.9 (3.010)	HOMO→LUMO (94.9 %)	1.4125	0.593

[a] Δ*E*_calcd–exp_=*E*_LR calcd_–*E*_abs exp_ [eV].

**Figure 6:**
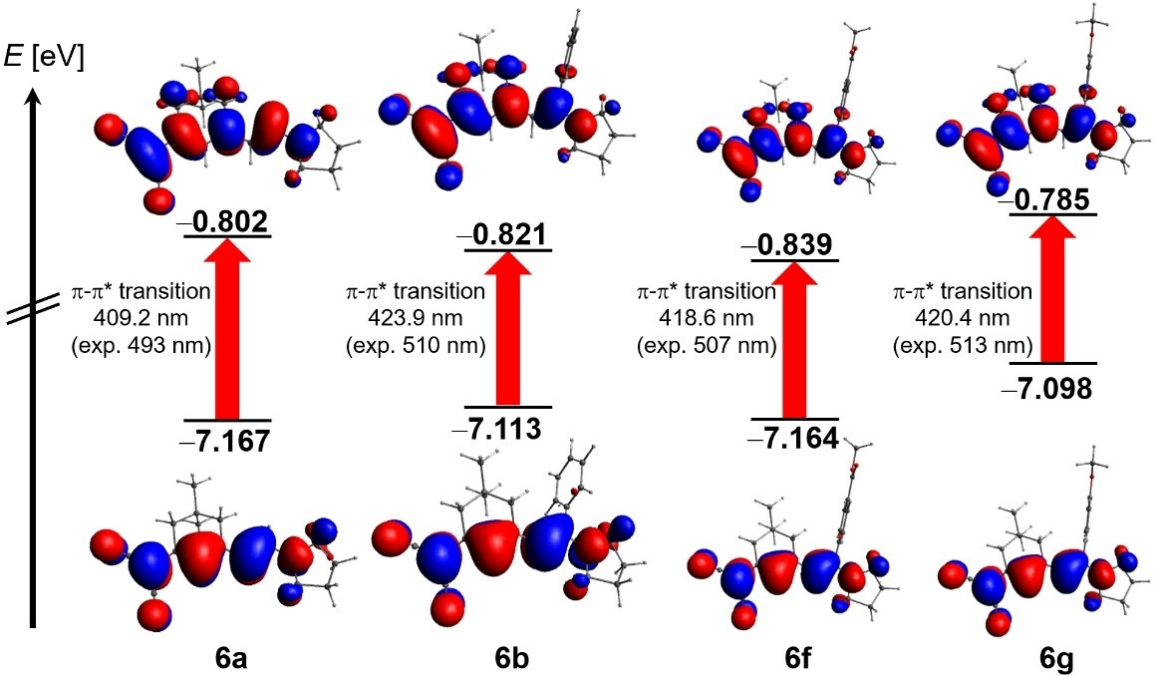
Selected Kohn‐Sham frontier molecular orbitals of merocyanines **6 a**, **6 b**, **6 f**, and **6 g** (Gaussian 16 LC‐ωB97XD/6‐311++G**, PCM CH_2_Cl_2_, isosurface value at 0.04) and the calculated HOMO and LUMO energies (in eV) as well as the calculated and experimentally determined absorption wavelengths (in nm).

The comparison of the three different functionals with corrected Linear Response and with Linear Response reveals that the B3LYP functional with LR gives the smallest, yet significant deviations between calculated and experimental longest wavelength absorption bands, overestimating the calculated S_1_ transitions by 0.422 to 0.451 eV (Table 2). However, plotting calculated against experimental S_1_ energies shows that exchange correlation hybrid functionals with LR correlated better with the experimental data (see Supporting Information, Figures S42‐S45), although they overestimate the transitions 0.494 to 0.532 eV (LC‐ωB97XD) and 0.583 to 0.595 eV (LC‐ωHPBE), respectively (Table 2). For all calculations, the longest wavelength bands are found as π–π*‐transitions with very large oscillator strengths, which are represented as cyanine typical HOMO‐LUMO transitions along the merocyanine chromophore axis as nicely seen from the Kohn‐Sham frontier molecular orbitals of the LC‐ωB97XD/6‐311++G** PCM calculations (Figure 6).

Finally, we also decided to rationalize the electronic structure of the observed weak, yet detectible emission of the merocyanines **6** by TDDFT calculations. Although, long‐range corrected (LC) hybrid density functionals overestimate the S_1_ energies in comparison to the experimental UV/Vis spectra, they systematically correlate well with experimental data. Therefore, the structures of the vibrationally relaxed S_1_ states of the merocyanines **6 a**, **6 b**, **6 f**, and **6 g** were optimized (LC‐ωB97XD/6‐311++G**, PCM CH_2_Cl_2_ and LC‐ωHPBE/6‐311++G**, PCM CH_2_Cl_2_; Table 3). As expected, the emission from the vibrationally relaxed S_1_ state to the vibrationally excited ground state S_0_^v^ overestimate these HOMO‐LUMO transitions in comparison to the experimental emission bands with 0.253 to 0.350 eV (LC‐ωB97XD) and 0.258 to 0.380 eV (LC‐ωHPBE), respectively. Consequently, also the experimental Stokes shifts between 800 to 2200 cm^−1^ are overestimated by the calculated date of the LC‐ωB97XD (2904 to 4065 cm^−1^) and LC‐ωHPBE functional (3404 to 3912 cm^−1^), however, in both cases describing the correct trend as seen in the experimental data. A major reason for the deviation of calculated from experimental absorption and emission data might arise from the occurrence of several conformations at room temperature, as seen from the coalescence in the NMR spectra. Most distinctly, the merocyanines **6** represent dyes with cyanine character, yet, with larger Stokes shifts than typically observed for cyanines.

**Table 3:** Experimentally determined (recorded in dichloromethane at *T*=298 K) and TDDFT calculated (LC‐ωB97XD, LC‐ωHPBE with LR (linear response) and 6311++G** as a basis set) longest wavelength absorption bands and shortest wavelength emission bands of merocyanines **6 a**, **6 b**, **6 f**, and **6 g** and Stokes shifts $\Delta \tilde{\upsilon }$ .

dye	λ_max,abs_ [nm] ([eV])	λ_max,em_ [nm] ([eV])	Δ$\tilde{\upsilon }$ cm^−1^ [eV]^[a]^	functional	λ_max,abs_ [nm] ([eV])	λ_max,em_ [nm] ([eV])	$\Delta \tilde{\upsilon }$ [cm^−1^] ([eV])^[a]^
**6 a**	493 (2.515)	513 (2.417)	800 (0.098)	LC‐ωB97XD	409.2 (3.030)	464.4 (2.670)	2904 (0.360)
				LC‐ωHPBE	400.4 (3.097)	463.5 (2.675)	3404 (0.422)
**6 b**	510 (2.431)	550 (2.254)	1400 (0.177)	LC‐ωB97XD	423.9 (2.925)	487 (2.546)	3057 (0.379)
				LC‐ωHPBE	410.2 (3.023)	480.1 (2.583)	3549 (0.440)
**6 f**	507 (2.445)	570 (2.175)	2200 (0.270)	LC‐ωB97XD	418.6 (2.962)	504.5 (2.458)	4065 (0.504)
				LC‐ω HPBE	407.8 (3.040)	485.2 (2.555)	3912 (0.485)
**6 g**	513 (2.417)	559 (2.218)	1600 (0.199)	LC‐ωB97XD	420.4 (2.949)	482.8 (2.568)	3073 (0.381)
				LC‐ωHPBE	411.9 (3.010)	478.6 (2.591)	3379 (0.419)

[a] Δ$\tilde{\upsilon }$ =1/*λ*_*max,abs*_−1/*λ*_*max,em*_ [cm^−1^].

## Conclusions

A triflate, readily obtained from dimedone (5,5‐dimethylcyclohexane‐1,3‐dione) by Knoevenagel condensation with malonodinitrile and triflation, sets the stage for a concise and efficient consecutive three‐component alkynylation‐addition synthesis of a small library of merocyanines with strong absorption coefficients for the longest wavelength absorption bands. Inspection of a crystal structure reveals that these merocyanines are cyanine like with small bond length alternation. This ground state delocalization is additionally supported by a VT‐NMR analysis of the intermolecular dynamics giving rise to a free enthalpy of activation for the rotation about the CN bond of Δ*G_c_* ^*ǂ*^=61 kJ/mol and a free enthalpy of activation for the pyrrolidinyl nitrogen inversion of Δ*G_c_* ^*ǂ*^=51 kJ/mol. The dyes are intensively red to violet solids and their solutions are intensively colored and their emission is weak, but detectable. Elucidation of the electronic structure by TDDFT calculations reveals that the trend of the absorption and emission bands can rationalized by intense HOMO‐LUMO transitions along the merocyanine axis leading to larger Stokes shifts than cyanines when aryl substituents are placed at the donor terminus. The straightforward synthetic one‐pot approach to merocyanines with broad absorption bands and high absorption coefficients enables an easy access to dye libraries with tunable electronics introduced at three points of diversity, that is, the acceptor part, the alkynyl moiety and the amine component. This can be favorably applied to establishing novel merocyanine libraries for absorbers in organic photovoltaics. Further studies directed to explore this concept are currently underway.

## Supporting Information

The Supporting Information contains experimental details on the synthesis and analytics of compounds **3**, **Aa**, **Ab**, **6**, and **8**, on the optimization of the consecutive three‐component reaction, NMR spectra of compounds **3**, **6**, and **8**, UV/Vis and emission spectra of compounds **6** and **8**, data of the X‐ray structure of compound **6 h**, data for determining the barrier of merocyanine **6 f** by VT NMR spectra, and computational data of merocyanines **6 a**, **6 b**, **6 f**, and **6 g**.

## Conflict of interest

The authors declare no conflict of interest.

1

## Supporting information

As a service to our authors and readers, this journal provides supporting information supplied by the authors. Such materials are peer reviewed and may be re‐organized for online delivery, but are not copy‐edited or typeset. Technical support issues arising from supporting information (other than missing files) should be addressed to the authors.

Supporting Information

## Data Availability

The data that support the findings of this study are available in the supplementary material of this article.
