# Supplementary material for: Cyclohexene‐Embedded Dicyanomethylene Merocyanines – Consecutive Three‐Component Coupling‐Addition Synthesis and Chromophore Characteristics
Source: ChemistryOpen. 2023 Sep 15;12(9):e202300128. doi: 10.1002/open.202300128 (PMC10504436; doi:10.1002/open.202300128)
Supplement: Supplementary file 1 — Supporting Information [file OPEN-12-e202300128-s001.pdf]

ChemistryOpen

Supporting Information

Cyclohexene-Embedded Dicyanomethylene Merocyanines – Consecutive Three-Component Coupling-Addition Synthesis and Chromophore Characteristics

Julian Papadopoulos, Guido J. Reiss, Bernhard Mayer, and Thomas J. J. Müller*

Table of Contents

1. General considerations ... 4
2. Starting materials ... 5
2.1. 2-(3-Hydroxy-5,5-dimethylcyclohex-2-en-1-ylidene)malononitrile ... 5
2.2. 3-(Dicyanomethylene)-5,5-dimethylcyclohex-1-en-1-yl trifluoromethanesulfonate (**3**) ... 5
3. Optimization of the Consecutive Three-component Reaction ... 7
3.1. Synthesis of Alkyne Intermediates **A** ... 7
3.1.1. 2-{5,5-Dimethyl-3-[(trimethylsilyl)ethynyl]cyclohex-2-en-1-ylidene}malononitrile (**Aa**). ... 8
3.1.2. 2-[5,5-Dimethyl-3-(phenylethynyl)cyclohex-2-en-1-ylidene]malononitrile (**Ab**) ... 8
3.2. Optimization of the Alkynylation within the Sequence ... 9
4. Syntheses of Merocyanines **4** and **6** ... 11
4.1. General procedure (GP) for the consecutive three-component synthesis of merocyanines **4** and **6** ... 11
4.2. (*E*)-2-{5,5-Dimethyl-3-[2-(pyrrolidin-1-yl)vinyl]cyclohex-2-en-1-ylidene}malononitrile (**6a**) ... 12
4.3. (*E*)-2-{5,5-Dimethyl-3-[2-phenyl-2-(pyrrolidin-1-yl)vinyl]cyclohex-2-en-1-ylidene}malononitrile (**6b**) ... 12
4.4. (*E*)-2-{5,5-Dimethyl-3-[2-(piperidin-1-yl)vinyl]cyclohex-2-en-1-ylidene}malononitrile (**6c**) ... 13
4.5. (*E*)-2-[5,5-Dimethyl-3-(2-morpholino-2-phenylvinyl)cyclohex-2-en-1-ylidene]malononitrile (**6d**) ... 14
4.6. (*E*)-2-{5,5-Dimethyl-3-[2-phenyl-2-(piperidin-1-yl)vinyl]cyclohex-2-en-1-ylidene}malononitrile (**6e**) ... 15
4.7. Methyl-(*E*)-4-{2-[3-(dicyanomethylene)-5,5-dimethylcyclohex-1-en-1-yl]-1-(pyrrolidin-1-yl)vinyl}benzoate (**6f**) ... 15
4.8. (*E*)-2-{3-[2-(4-Methoxyphenyl)-2-(pyrrolidin-1-yl)vinyl]-5,5-dimethylcyclohex-2-en-1-ylidene}malononitrile (**6g**) ... 16
4.9. (*E*)-2-(3-{2-[(4-Methoxyphenyl)amino]-2-phenylvinyl}-5,5-dimethylcyclohex-2-en-1-ylidene)malononitrile (**6h**) ... 17
4.10. 2,2'-{[(1*E*,1'*E*)-Piperazin-1,4-diylbis(2-phenylethen-2,1-diyl)]bis(5,5-dimethylcyclohex-2-en-3-yl-1-ylidene)}dimalononitrile (**6i**) ... 18
4.11. 2-{5,5-Dimethyl-3-[3-(1,3,3-trimethylindolin-2-ylidene)-2-(trimethylsilyl)prop-1-en-1-yl]cyclohex-2-en-1-ylidene}malononitrile (**8a**) ... 18
4.12. 2-{5,5-Dimethyl-3-[2-phenyl-3-(1,3,3-trimethylindolin-2-ylidene)prop-1-en-1-yl]cyclohex-2-en-1-ylidene}malononitrile (**6b**) ... 19
4.13. 2-{3-[2-(4-Methoxyphenyl)-3-(1,3,3-trimethylindolin-2-ylidene)prop-1-en-1-yl]-5,5-dimethylcyclohex-2-en-1-ylidene}malononitrile (**8c**) ... 20
5. NMR Spectra of Compound **3** and Merocyanines **4** and **6** ... 21
5.1. 3-(Dicyanomethylene)-5,5-dimethylcyclohex-1-en-1-yl trifluoromethanesulfonate (**3**) ... 21
5.2. (*E*)-2-{5,5-Dimethyl-3-[2-(pyrrolidin-1-yl)vinyl]cyclohex-2-en-1-ylidene}malononitrile (**6a**) ... 22
5.3. (*E*)-2-{5,5-Dimethyl-3-[2-phenyl-2-(pyrrolidin-1-yl)vinyl]cyclohex-2-en-1-ylidene}malononitrile (**6b**) ... 23
5.4. (*E*)-2-{5,5-Dimethyl-3-[2-(piperidin-1-yl)vinyl]cyclohex-2-en-1-ylidene}malononitrile (**6c**) ... 24
5.5. (*E*)-2-[5,5-Dimethyl-3-(2-morpholino-2-phenylvinyl)cyclohex-2-en-1-ylidene]malononitrile (**6d**) ... 25
5.6. (*E*)-2-{5,5-Dimethyl-3-[2-phenyl-2-(piperidin-1-yl)vinyl]cyclohex-2-en-1-ylidene}malononitrile (**6e**) ... 26

5.7. Methyl-(*E*)-4-{2-[3-(dicyanomethylene)-5,5-dimethylcyclohex-1-en-1-yl]-1-(pyrrolidin-1-yl)vinyl}benzoate (**6f**) 27
5.8. (*E*)-2-{3-[2-(4-Methoxyphenyl)-2-(pyrrolidin-1-yl)vinyl]-5,5-dimethylcyclohex-2-en-1-ylidene}malononitrile (**6g**) 28
5.9. (*E*)-2-(3-{2-[(4-Methoxyphenyl)amino]-2-phenylvinyl}-5,5-dimethylcyclohex-2-en-1-ylidene)malononitrile (**6h**) 29
5.10. 2,2'-{[(1*E*,1'*E*)-Piperazin-1,4-diylbis(2-phenylethen-2,1-diyl)]bis(5,5-dimethylcyclohex-2-en-3-yl-1-ylidene)}dimalononitrile (**6i**) 30
5.11. 2-{5,5-Dimethyl-3-[3-(1,3,3-trimethylindolin-2-ylidene)-2-(trimethylsilyl)prop-1-en-1-yl]cyclohex-2-en-1-ylidene}malononitrile (**8a**) 31
5.12. 2-{5,5-Dimethyl-3-[2-phenyl-3-(1,3,3-trimethylindolin-2-ylidene)prop-1-en-1-yl]cyclohex-2-en-1-ylidene}malononitrile (**6b**) 32
5.13. 2-{3-[2-(4-Methoxyphenyl)-3-(1,3,3-trimethylindolin-2-ylidene)prop-1-en-1-yl]-5,5-dimethylcyclohex-2-en-1-ylidene}malononitrile (**8c**) 33
6. UV/Vis and Emission Spectra of Merocyanines **4** and **6** 34
6.1. (*E*)-2-{5,5-Dimethyl-3-[2-(pyrrolidin-1-yl)vinyl]cyclohex-2-en-1-ylidene}malononitrile (**6a**) 34
6.2. (*E*)-2-{5,5-Dimethyl-3-[2-phenyl-2-(pyrrolidin-1-yl)vinyl]cyclohex-2-en-1-ylidene}malononitrile (**6b**) 34
6.3. (*E*)-2-{5,5-Dimethyl-3-[2-(piperidin-1-yl)vinyl]cyclohex-2-en-1-ylidene}malononitrile (**6c**) 35
6.4. (*E*)-2-[5,5-Dimethyl-3-(2-morpholino-2-phenylvinyl)cyclohex-2-en-1-ylidene]malononitrile (**6d**) 35
6.5. (*E*)-2-{5,5-Dimethyl-3-[2-phenyl-2-(piperidin-1-yl)vinyl]cyclohex-2-en-1-ylidene}malononitrile (**6e**) 36
6.6. Methyl-(*E*)-4-{2-[3-(dicyanomethylene)-5,5-dimethylcyclohex-1-en-1-yl]-1-(pyrrolidin-1-yl)vinyl}benzoate (**6f**) 36
6.7. (*E*)-2-{3-[2-(4-Methoxyphenyl)-2-(pyrrolidin-1-yl)vinyl]-5,5-dimethylcyclohex-2-en-1-ylidene}malononitrile (**6g**) 37
6.8. (*E*)-2-(3-{2-[(4-Methoxyphenyl)amino]-2-phenylvinyl}-5,5-dimethylcyclohex-2-en-1-ylidene)malononitrile (**6h**) 37
6.9. 2,2'-{[(1*E*,1'*E*)-Piperazin-1,4-diylbis(2-phenylethen-2,1-diyl)]bis(5,5-dimethylcyclohex-2-en-3-yl-1-ylidene)}dimalononitrile (**6i**) 38
6.10. 2-{5,5-Dimethyl-3-[3-(1,3,3-trimethylindolin-2-ylidene)-2-(trimethylsilyl)prop-1-en-1-yl]cyclohex-2-en-1-ylidene}malononitrile (**8a**) 38
6.11. 2-{5,5-Dimethyl-3-[2-phenyl-3-(1,3,3-trimethylindolin-2-ylidene)prop-1-en-1-yl]cyclohex-2-en-1-ylidene}malononitrile (**6b**) 39
6.12. 2-{3-[2-(4-Methoxyphenyl)-3-(1,3,3-trimethylindolin-2-ylidene)prop-1-en-1-yl]-5,5-dimethylcyclohex-2-en-1-ylidene}malononitrile (**8c**) 39
7. Crystal Structure of (*E*)-2-(3-{2-[(4-Methoxyphenyl)amino]-2-phenylvinyl}-5,5-dimethylcyclohex-2-en-1-ylidene)malononitrile (**6h**) 40
8. Determining the Barriers from the VT ^{1}H NMR Spectra of Merocyanine **6f** 42
9. Computational Data of Merocyanines **6a**, **6b**, **6f**, and **6g** 44
9.1. Computed xyz-coordinates (LC-ωB97XD/6-311++G** IEFPCM, dichloromethane) and excitation energies (LC-ωB97XD/6-311++G**) of merocyanine **6a** 44
9.2. Computed xyz-coordinates (LC-ωB97XD/6-311++G** IEFPCM, dichloromethane) S_1 and emission energies (LC-ωB97XD/6-311++G**) of merocyanine **6a** 46
9.3. Computed xyz-coordinates (LC-ωHPBE/6-311++G** IEFPCM, dichloromethane) and excitation energies (LC-ωHPBE/6-311++G**) of merocyanine **6a** 47
9.4. Computed xyz-coordinates (LC-ωHPBE6-311++G** IEFPCM, dichloromethane) S_1 and emission energies (LC-ωHPBE/6-311++G**) of merocyanine **6a** 49
9.5. Computed xyz-coordinates (LC-ωB97XD/6-311++G** IEFPCM, dichloromethane) and excitation energies (LC-ωB97XD/6-311++G**) of merocyanine **6b** 50
9.6. Computed xyz-coordinates (LC-ωB97XD/6-311++G** IEFPCM, dichloromethane) S_1 and emission energies (LC-ωB97XD/6-311++G**) of merocyanine **6b** 52

9.7. Computed xyz-coordinates (LC-ωHPBE/6-311++G** IEFPCM, dichloromethane) and excitation energies (LC-ωHPBE/6-311++G**) of merocyanine **6b** 54
9.8. Computed xyz-coordinates (LC-ωHPBE6-311++G** IEFPCM, dichloromethane) S_1 and emission energies (LC-ωHPBE/6-311++G**) of merocyanine **6b** 56
9.9. Computed xyz-coordinates (LC-ωB97XD/6-311++G** IEFPCM, dichloromethane) and excitation energies (LC-ωB97XD/6-311++G**) of merocyanine **6f** 58
9.10. Computed xyz-coordinates (LC-ωB97XD/6-311++G** IEFPCM, dichloromethane) S_1 and emission energies (LC-ωB97XD/6-311++G**) of merocyanine **6f** 60
9.11. Computed xyz-coordinates (LC-ωHPBE/6-311++G** IEFPCM, dichloromethane) and excitation energies (LC-ωHPBE/6-311++G**) of merocyanine **6f** 61
9.12. Computed xyz-coordinates (LC-ωHPBE6-311++G** IEFPCM, dichloromethane) S_1 and emission energies (LC-ωHPBE/6-311++G**) merocyanine **6f** 64
9.13. Computed xyz-coordinates (LC-ωB97XD/6-311++G** IEFPCM, dichloromethane) and excitation energies (LC-ωB97XD/6-311++G**) of merocyanine **6g** 65
9.14. Computed xyz-coordinates (LC-ωB97XD/6-311++G** IEFPCM, dichloromethane) S_1 and emission energies (LC-ωB97XD/6-311++G**) of merocyanine **6g** 68
9.15. Computed xyz-coordinates (LC-ωHPBE/6-311++G** IEFPCM, dichloromethane) and excitation energies (LC-ωHPBE/6-311++G**) of merocyanine **6g** 69
9.16. Computed xyz-coordinates (LC-ωHPBE6-311++G** IEFPCM, dichloromethane) S_1 and emission energies (LC-ωHPBE/6-311++G**) of merocyanine **6g** 72
9.17. Calculated S1 Excitation Energies and Deviations from the Experimental Longest Wavelength Absorption Bands of Merocyanines **6a**, **6b**, **6f**, and **6g** 73
10. References 77

1. General considerations

All reactions were carried out in Schlenk tubes or microwave vials under a nitrogen atmosphere. Solvents were dried by a solvent purification system (M. Braun Inertgas-Systeme GmbH/Garching MB-SPS-800). Dielectric heating was performed in a single mode microwave cavity producing continuous irradiation at 2450 MHz. Further purification of the compounds was performed by flash column chromatography (silica gel 60, mesh 230-400, MN). TLC: silica coated aluminium plates (60, F_{254}, Merck).^{1}H, ^{13}C, DEPT and NOESY NMR spectra were recorded in $CDCl_3$ (^{1}H δ 7.26, ^{13}C δ 77.2) or DMSO-d_6 (^{1}H δ 2.50, ^{13}C δ 39.52) on 300MHz (Bruker AVIII) or 600 MHz (Bruker Avance III-600) NMR spectrometers. The assignments of C_{quat}, CH, CH_2 and CH_3 nuclei were based on DEPT spectra. The elemental analyses were carried out in the microanalytical laboratory on a Perkin Elmer Series II Analyser 2400 of the Pharmazeutisches Institut of the Heinrich-Heine-Universität Düsseldorf. Mass spectra were recorded with a triple-quadrupole mass spectrometer (Finnigan MAR) or the ESI Ion-Trap-API-mass spectrometer Finnigan LCQ Deca (Thermo Quest). High resolution mass spectra were measured with a UHR-QTOF maxis 4G (Bruker Daltonics). Infrared spectra were recorded with a Shimadzu IR Affinity-1 with ATR technique. The intensities of signals are abbreviated as s (strong), m (medium) and w (weak). Absorption spectra were recorded in CH_2Cl_2 at 298 K on Perkin Elmer UV/VIS/NIR Lambda 19 Spectrometer. All solution spectra were recorded with dyes dissolved in spectroscopic grade solvents at 298 K using 1 cm-$_{quat}$z cuvettes from *Hellma GmbH*. The molar extinction coefficients of dye solutions of known dye concentration were determined by five-point regression line. Emission spectra were recorded in CH_2Cl_2 at 298 K on a Hitachi F7000 spectrometer (excitation at the longest wavelength absorption maximum).

2. Starting materials

2.1. 2-(3-Hydroxy-5,5-dimethylcyclohex-2-en-1-ylidene)malononitrile (2)[1]

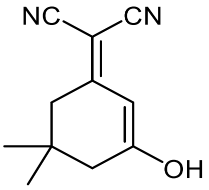

$C_{11}H_{12}N_2O$ [188.23]

Dimedone (**1**) (5.61 g, 40.0 mmol) and malononitrile (4.36 g, 40.0 mmol) were placed in round bottom flask with magnetic stir bar under nitrogen and ethanol (50 mL) was added. Piperidine (12 drops) were added to the reaction mixture, which was heated to reflux for 6 h. After cooling to room temp the solvents were removed in vacuo and the residue was purified by chromatography on silica gel (hexane/ethyl acetate3:1) to give after drying in vacuo 2-(3-hydroxy-5,5-dimethylcyclohex-2-en-1-ylidene)malononitrile (**2**) (5.58 g, 74%) as a yellow solid. Mp 123 °C, R_f (*n*-hexane/ethyl acetate 3:1): 0.33.

^{1}H NMR (600 MHz, $CDCl_3$): δ 1.07 (s, 6 H), 2.29 (s, 2 H), 2.52 (s, 2 H), 6.16 (s, 1 H), 9.40 (s, 1 H). ^{13}C NMR (151 MHz, $CDCl_3$): δ 27.9 (CH_3), 33.0 (C_{quat}), 42.6 (CH_2), 68.5 (C_{quat}), 100.3 (CH), 113.7 (C_{quat}), 114.1 (C_{quat}), 174.7 (C_{quat}), 176.9 (C_{quat}). EI-MS (70 eV, *m*/*z* (%)): 188 (6, [M^+]), 168 (64), 153 (15), 140 (12), 112 (93), 111 (13), 84 (100), 69 (57), 68 (59), 55 (13), 43 (23), 41 (12), 39 (11). HR MS (ESI, *m*/*z* (%)) calcd. for $[C_{11}H_{12}N_2ONa]^+$: 211.0842; Found: 211.0842.

2.2. 3-(Dicyanomethylene)-5,5-dimethylcyclohex-1-en-1-yl trifluoromethanesulfonate (3)[1]

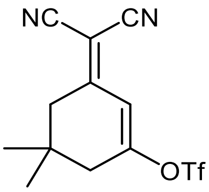

3

$C_{12}H_{11}F_3N_2O_3S$ [320.29]

2-(3-Hydroxy-5,5-dimethylcyclohex-2-en-1-ylidene)malononitrile (**2**) (1.88 g, 10 mmol) and triethylamine (1.8 mL, 13 mmol) were placed in round bottom flask with magnetic stir bar under nitrogen, dissolved in dichloromethane (50 mL) and cooled to 0 °C (water-ice bath). To this mixture was slowly added dropwise trifluoromethanesulfonic anhydride (2.18 mL, 13 mmol). The cooling was removed and stirring at room temp was continued for 2 h. The solvent was removed under reduced pressure and the crude product was purified by chromatography on

silica gel (dichloromethane) to give triflate **3** (2.18 g, 68%) as a yellow solid. R_f (dichloromethane): 0.98.

^{1}H NMR (600 MHz, $CDCl_3$): δ 1.13 (s, 6 H), 2.52 (d, *J* = 1.5 Hz, 2 H), 2.61 (s, 2 H), 6.77 (t, *J* = 1.4 Hz, 1 H). ^{19}F NMR (282 MHz, $CDCl_3$): δ 73.25. ^{13}C NMR (151 MHz, $CDCl_3$): δ 27.7 (CH_3), 33.8 (C_{quat}), 42.2 (CH_2), 42.5 (CH_2), 84.7 (C_{quat}), 111.0 (C_{quat}), 111.8 (C_{quat}), 115.0 (CH), 118.5 (q, *J* = 320.7 Hz, CF_3), 161.7 (C_{quat}), 167.0 (C_{quat}). EI-MS (70 eV, *m*/*z* (%)): 321 (13), 320 (88, [M^+]), 305 (18, [$C_{11}H_8N_2O_3SF_3^+$]), 241 (46), 228 (46), 227 (26), 216 (15), 214 (47), 201 (10), 200 (56), 187 (10, [$C_{11}H_{11}N_2O^+$]), 172 (49), 171 (23, [$C_{11}H_{11}N_2^+$]), 170 (16), 169 (21), 160 (11), 159 (16, [$C_{10}H_{11}N_2^+$]), 157 (12), 156 (24), 155 (78), 145 (17), 144 (39), 143 (44), 142 (25), 132 (11), 131 (13), 130 (14), 129 (15), 128 (37), 118 (10), 117 (19), 116 (25), 115 (21), 113 (20), 105 (16), 104 (14), 103 (19, [$C_6H_3N_2^+$]), 91 (13), 89 (11, [$C_5HN_2^+$]), 79 (16), 77 (28), 76 (16, [$C_4N_2^+$]), 69 (100, [CF_3^+]), 56 (17), 55 (15), 43 (22), 41 (34), 39 (22). IR: $\tilde{\nu}$ [cm^{-1}]: 2967 (w), 2878 (w), 2230 (w), 1636 (m), 1564 (w), 1470 (w), 1427 (m), 1373 (w), 1339 (w), 1317 (w), 1292 (w), 1277 (w), 1248 (m), 1209 (s), 1134 (s), 1076 (s), 1024 (w), 1005 (w), 986 (w), 934 (m), 916 (m), 881 (s), 845 (s), 785 (m), 746 (s), 652 (w), 602 (s). Anal. calcd. for $C_{12}H_{11}F_3N_2O_3S$ (320.0): C 45.00, H 3.46 N 8.75, S 10.01; Found: C 45.16, H 3.45, N 8.67, S 10.08.

3. Optimization of the Consecutive Three-component Reaction

For the synthesis of the merocyanines **6**, the standard conditions with the catalyst system $PdCl_2(PPh_3)_2$/CuI and triethylamine as a base[2] were first chosen for the Sonogashira coupling using (TMS)acetylene (**4a**) and phenylacetylene (**4b**) as alkynes (Scheme S1). The Michael addition was performed under microwave irradiation at 100 °C for 1 h.

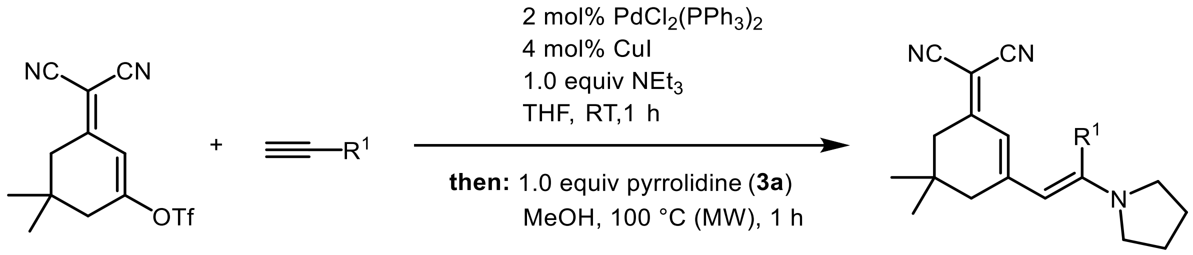

3 **4** **6a** (R^1 = H, 37%) **6b** (R^1 = Ph, 59%)

Scheme S1. Three-component reaction for the synthesis of merocyanines **6** under standard conditions.[2]

3.1. Synthesis of Alkyne Intermediates A

Due to the moderate yields of 37 and 59 %, respectively, the three-component synthesis was optimized. In order to determine which partial reaction of the three-component reaction is the problem and thus responsible for the low yields, the intermediate alkynes **A** were first isolated. Using $PdCl_2(PPh_3)_2$ and NEt_3, alkyne **Aa** was isolated in 39% yield and alkyne **Ab** in 20% yield (Scheme S2).

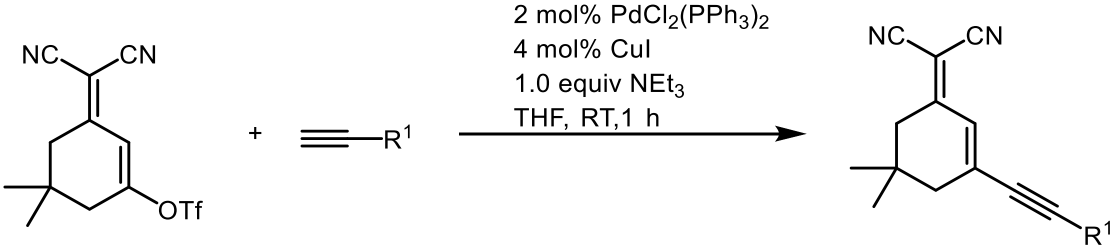

3 **4** **Aa** (R^1 = TMS, 85%) **Ab** (R^1 = Ph, 81%)

Scheme S2. Sonogashira coupling under standard conditions.

The low yields of the Sonogashira reaction indicated that the subsequent Michael addition obviously proceeded nearly quantitatively. Therefore, the parameters for Sonogashira coupling in the three-component sequence were optimized. Indeed, DIPEA (diisopropylethylamine) turned out to be a favorable base for efficiently performing the Sonogashira alkynylation.

3.1.1. 2-{5,5-Dimethyl-3-[(trimethylsilyl)ethynyl]cyclohex-2-en-1-ylidene}malononitrile (Aa)

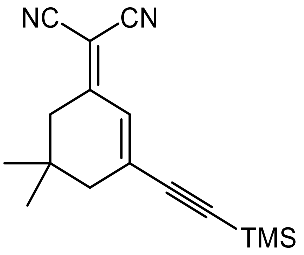

$C_{16}H_{20}N_2Si$ [268.44]

In a microwave vessel with magnetic stir bar were placed $PdCl_2(PPh_3)_2$ (2 mol%, 14 mg), CuI (4 mol%, 7 mg), and triflate **3** (0.32 g, 1.00 mmol) in dry, degassed THF (1 mL). After addition of (TMS)acetylene (**4a**) (0.14 mL, 0.98 mmol)) and DIPEA (0.17 mL, 1.00 mmol) the reaction mixture was stirred under nitrogen at room temp for 1 h. The solvents were removed in vacuo and the crude product was purified by flash chromatography on silica gel (*n*-hexane/ethyl acetate 10:1) to give compound **Aa** (228 mg, 85%) as a yellow solid, Mp 104 °C, R_f (*n*-hexane/ethyl acetate 10:1): 0.39.

^{1}H NMR (600 MHz, $CDCl_3$): δ 0.23 (s, 9 H), 1.03 (s, 6 H), 2.30 (d, J = 1.8 Hz, 2 H), 2.53 (s, 2 H), 7.00 (t, J = 1.7 Hz, 1 H). ^{13}C NMR (151 MHz, $CDCl_3$): δ -0.3 (CH_3), 27.8 (CH_3), 32.3 (C_{quat}), 42.4 (CH_2), 44.6 (CH_2), 80.9 (C_{quat}), 104.5 (C_{quat}), 107.9 (C_{quat}), 112.1 (C_{quat}), 112.9 (C_{quat}), 128.5 (CH), 139.3 (C_{quat}), 168.2 (C_{quat}). EI-MS (70 eV, *m*/*z* (%)): 268 (16, [M^+]), 254 (22), 253 (100, [$C_{15}H_{17}N_2Si^+$]), 226 (13), 97 (10, [$C_5H_9Si^+$]). IR: $\tilde{\nu}$ [cm^{-1}]: 2963 (w), 2936 (w), 2222 (w), 1570 (s), 1541 (m), 1522 (w), 1508 (w), 1474 (w), 1466 (w), 1458 (w), 1452 (w), 1416 (w), 1393 (w), 1364 (w), 1319 (w), 1281 (w), 1250 (m), 1207 (w), 1167 (w), 1148 (w), 1138 (w), 1130 (w), 930 (w), 895 (w), 876 (m), 841 (s), 760 (m), 760 (m), 760 (m), 702 (w), 652 (m), 636 (m). UV/Vis (CH_2Cl_2): λ_{max} [nm] (ε [$Lcm^{-1}mol^{-1}$]): 338 (22900). Anal. calcd. for $C_{16}H_{20}N_2Si$ (268.1): C 71.59, H 7.51, N 10.44; Found: C 71.72, H 7.64, N 10.46.

3.1.2. 2-[5,5-Dimethyl-3-(phenylethynyl)cyclohex-2-en-1-ylidene]malononitrile (Ab)

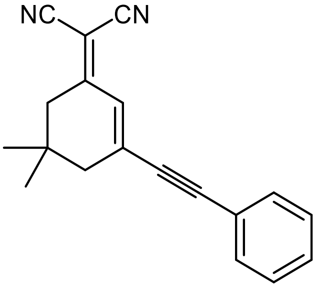

$C_{19}H_{16}N_2$ [272.35]

In a microwave vessel, $PdCl_2(PPh_3)_2$ (2 mol%, 14 mg), CuI (4 mol%, 7 mg), and compound **3** (160 mg, 0.50 mmol) were placed in dry and degassed THF (1 mL) under nitrogen. After addition of phenylacetylene (**4b**) (50 mg, 0.50 mmol) and DIPEA (86 µL, 0.50 mmol), the reaction mixture was stirred at room temperature for 1 h. The solvent was removed under reduced pressure and the crude product was purified by column chromatography on silica gel (*n*-hexane/ethyl acetate 10:1) to give compound **Ab** (110 mg, 81%) as a yellow solid, Mp 157 °C, R_f (*n*-hexane/ethyl acetate 10:1): 0.37.

^{1}H NMR (600 MHz, $CDCl_3$): δ 1.07 (s, 6 H), 2.42 (d, J = 1.7 Hz, 2 H), 2.58 (s, 2 H), 7.08 (t, J = 1.7 Hz, 1 H), 7.35-7.44 (m, 3 H), 7.47-7.54 (m, 2 H). ^{13}C NMR (151 MHz, $CDCl_3$): δ 27.8 (CH_3), 32.3 (C_{quat}), 42.4 (CH_2), 44.7 (CH_2), 80.3 (C_{quat}), 90.2 (C_{quat}), 101.3 (C_{quat}), 112.3 (C_{quat}), 113.1 (C_{quat}), 121.7 (C_{quat}), 127.7 (CH), 128.8 (CH), 130.1 (CH), 132.4 (CH), 139.7 (C_{quat}), 168.1 (C_{quat}). EI-MS (70 eV, m/z (%)): 273 (22), 272 (100, [M^+]), 257 (22, [$C_{18}H_{13}N_2^+$]), 242 (17, [$C_{17}H_{10}N_2^+$]), 230 (18), 115 (12), 43 (10). IR: $\tilde{\nu}$ [cm^{-1}]: 2967 (w), 2216 (w), 2185 (w), 1570 (m), 1558 (s), 1533 (m), 1506 (w), 1489 (w), 1472 (w), 1443 (w), 1416 (m), 1367 (m), 1323 (m), 1294 (w), 1155 (m), 868 (s), 760 (s), 690 (s). UV/Vis (CH_2Cl_2): λ_{max} [nm] (ε [$Lcm^{-1}mol^{-1}$]): 254 (10800), 374 (56800). Emission (CH_2Cl_2): λ_{max} [nm]: 499. Stokes shift [cm^{-1}]: 6700. Anal. calcd. for $C_{19}H_{16}N_2$ (272.1): C 83.79, H 5.92, N 10.29; Found: C 84.06, H 6.15, N 9.99.

3.2. Optimization of the Alkynylation within the Sequence

In the model reaction for optimizing the alkynylation within the sequence triflate **3**, (TMS)acetylene (**4a**), and pyrrolidine (**5a**) were reacted upon variation of the Pd catalyst and the employed base maintaining the standard reaction temperatures and reaction times (RT for 1 h for the alkynylation, MW (100 °C) for 1 h for the Michael addition with addition of methanol as a cosolvent) to give merocyanine **6a** (Scheme S3, Table S1).

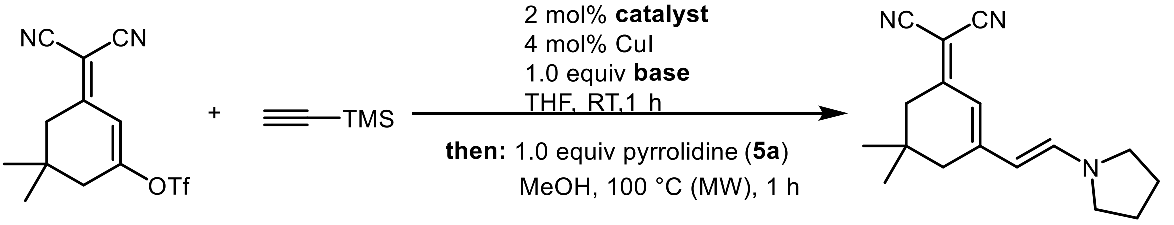

3 **4a** **6a**

Scheme S3. Optimization of the catalyst and the base in Sonogashira coupling step of the three-component synthesis of merocyanine **6a**.

In a first optimization approach of the three-component synthesis, a Pd^0 species, tetrakis(triphenylphosphane)palladium, was chosen instead of $PdCl_2(PPh_3)_2$. However, this resulted in a collapse of the yield to 22% (Table S1, entry 2). It was observed that the inorganic base K2CO3 did not lead to any conversion (Table S1, entry 3), however, diisopropylethylamine (DIPEA) doubled the yield to 68% (Table S1, entry 4). Subsequently,

the optimization was checked by using the phenylacetylene instead of TMSA (Table S1, entry 5) and merocyanine **6b** could was isolated in a yield of 95%. This represents a quantitative conversion to the desired reaction product.

Table S1. Catalyst and base optimization of the Sonogashira coupling within the three-component synthesis of merocyanines **6**.

entry	alkyne **4**	catalyst	base	compound **6**
1	**4a**	$PdCl_2(PPh_3)_2$	NEt_3	**6a** (37%)
2	**4a**	$Pd(PPh_3)_4$	NEt_3	**6a** (22%)
3	**4a**	$PdCl_2(PPh_3)_2$	K_2CO_3	– [a]
4	**4a**	$PdCl_2(PPh_3)_2$	DIPEA	**6a** (68%)
5	**4b**	$PdCl_2(PPh_3)_2$	DIPEA	**6b** (95%)

[a] No conversion to compound **6a** observed.

4. Syntheses of Merocyanines 6 and 8

4.1. General procedure (GP) for the consecutive three-component synthesis of merocyanines 6 and 8

In a microwave vessel with magnetic stir bar were placed $PdCl_2(PPh_3)_2$ (2 mol%, 14 mg), CuI (4 mol%, 7 mg), and triflate **3** (1.00 equiv.) in dry, degassed THF (1 mL) (for experimental details, see Table S1). After addition of alkyne **4** (1.00 equiv.) and DIPEA (1.00 equiv.) the reaction mixture was stirred under nitrogen at room temp for 1 h. Then, nucleophile **5** or **7** (1.00 equiv.) in methanol (1 mL) was added and the reaction mixture was heated in the microwave reactor at 100 °C for 1 h. After cooling to room temp the solvents were removed in vacuo and the crude product was purified by flash chromatography on silica gel to give merocyanines **6** or **8**.

Table S2. Experimental details for the consecutive three-component synthesis of merocyanines **6** or **8**.

entry	triflate **3**	Alkyne **4**	amine **5** or Fischer's base (**7**)	Yield of merocyanines **6** or **8**
1	160 mg (0.50 mmol)	70 mg (0.69 mmol) of **4a**	41 µL (0.50 mmol) of **5a**	90 mg (68 %) of **6a**
2	160 mg (0.50 mmol)	50 mg (0.50 mmol) **of 4b**	44 µL (0.50 mmol) of **5a**	160 mg (93%) of **6b**
3	160 mg (0.50 mmol)	70 mg (0.68 mmol) of **4a**	50 µL (0.50 mmol) of **5b**	85 mg (60 %) of **6c**
4	160 mg (0.50 mmol)	50 mg (0.50 mmol) of **4b**	44 µL (0.50 mmol) of **5c**	130 mg (72 %) of **6d**
5	160 mg (0.50 mmol)	50 mg (0.50 mmol) of **4b**	50 µL (0.50 mmol) of **5b**	143 mg (80 %) of **6e**
6	160 mg (0.50 mmol)	80 mg (0.50 mmol) of **4c**	41 µL (0.50 mmol) of **5a**	170 mg (85 %) of **6f**
7	160 mg (0.50 mmol)	66 mg (0.5 mmol) of **4d**	41 µL (0.50 mmol) of **5a**	105 mg (56 %) of **6g**
8	160 mg (0.50 mmol)	50 mg (0.50 mmol) of **4b**	62 mg (0.50 mmol) of *p*-anisidine (**5d**)	150 mg (76 %) of **6h**
9	320 mg (1.00 mmol)	100 mg (1.00 mmol) of **4b**	43 mg (0.5 mmol) of piperazine **5e**)	77 mg (24 %) of **6i**
10	160 mg (0.50 mmol)	70 mg (0.69 mmol) of **4a**	92 µL (0.50 mmol) of **7**	140 mg (63 %) of **8a**

11	0.16 g (0.50 mmol)	50 mg (0.50 mmol) of **4b**	92 µL (0.50 mmol) of **7**	178 mg (80 %) of **8b**
12	0.16 g (0.50 mmol)	66 mg (0.50 mmol) of **4d**	92 µL (0.50 mmol) of **7**	182 mg (76 %) of **8c**

4.2. (*E*)-2-{5,5-Dimethyl-3-[2-(pyrrolidin-1-yl)vinyl]cyclohex-2-en-1-ylidene}malononitrile (6a)

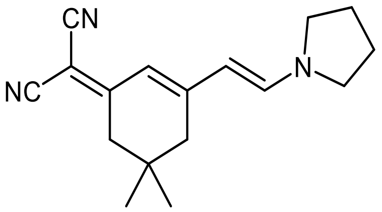

6a

$C_{17}H_{21}N_3$ [267.38]

According to the GP and after flash chromatography on silica gel (*n*-hexane/ethyl acetate 2:1) compound **6a** (90 mg, 68%) was obtained as a red solid, Mp 229 °C. R_f (*n*-hexane/ethyl acetate 2:1): 0.22. ^{1}H NMR (600 MHz, $CDCl_3$): δ 1.00 (s, 6 H), 1.89-2.09 (m, 4 H), 2.26 (s, 2 H), 2.45 (s, 2 H), 3.20-3.59 (m, 4 H), 5.24 (d, *J* = 12.7 Hz, 1 H), 6.36 (s, 1 H), 7.31 (d, *J* = 12.7 Hz, 1 H). ^{13}C NMR (151 MHz, $CDCl_3$): δ 25.3 (CH_2), 28.2 (CH_3), 32.0 (C_{quat}), 39.8 (CH_2), 42.8 (CH_2), 47.0 (CH_2), 53.0 (CH_2), 65.3 (C_{quat}), 102.1 (CH), 113.1 (CH), 116.1 (C_{quat}), 116.9 (C_{quat}), 144.8 (CH), 158.8 (C_{quat}), 167.8 (C_{quat}). EI-MS (70 eV, *m*/*z* (%)): 268 (20), 267 (100, [M^+]), 266 (25), 253 (12, [$C_{17}H_{21}N_2^+$]), 252 (63, [$C_{16}H_{18}N_3^+$]), 224 (10), 199 (11), 162 (15), 120 (25), 70 (17, [$C_4H_8N^+$]). IR: $\tilde{\nu}$ [cm^{-1}]: 2969 (w), 2930 (w), 2868 (w), 2195 (m), 2176 (m), 1611 (m), 1540 (s), 1537 (s), 1485 (s), 1458 (m), 1425 (m), 1406 (m), 1381 (m), 1341 (m), 1321 (m), 1273 (m), 1259 (m), 1213 (s), 1198 (m), 1184 (m), 1153 (s), 1142 (s), 1128 (m), 1112 (m), 1007 (m), 989 (m), 959 (m), 912 (m), 856 (s), 837 (m), 804 (m), 770 (m), 731 (m), 694 (m), 610 (m). UV/Vis (CH_2Cl_2): λ_{max} [nm] (ε [Lcm^{-1}mol^{-1}]): 493 (98000). Emission (CH_2Cl_2): λ_{max} [nm] (Φ_F): 513 (<1), Stokes shift [cm^{-1}]: 800. Anal. calcd. for $C_{17}H_{21}N_3$ (267.2): C 76.37, H 7.92, N 15.72; Found: C 76.08, H 8.20, N 15.63.

4.3. (*E*)-2-{5,5-Dimethyl-3-[2-phenyl-2-(pyrrolidin-1-yl)vinyl]cyclohex-2-en-1-ylidene}malononitrile (6b)

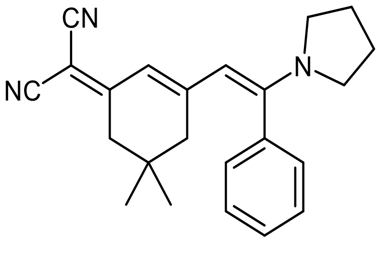

6b

$C_{23}H_{25}N_3$ [343.47]

According to the GP and after flash chromatography on silica gel (*n*-hexane/ethyl acetate 10:1) compound **6b** (160 mg, 93%) was obtained as a red solid, Mp 146 °C. R_f (*n*-hexane/ethyl acetate 10:1): 0.31. ^{1}H NMR (600 MHz, $CDCl_3$): δ 0.74 (s, 6 H), 1.60 (d, *J* = 2.3 Hz, 2 H), 1.88-2.02 (m, 4 H), 2.26 (s, 2 H), 2.9-3.6 (m, 4 H), 5.31 (s, 1 H), 6.07 (s, 1 H), 7.20-7.22 (m, 2 H), 7.44-7.50 (m, 3 H). ^{13}C NMR (151 MHz, $CDCl_3$): δ 25.4 (CH_2), 27.8 (CH_3), 31.8 (C_{quat}), 42.3 (CH_2), 43.9 (CH_2), 49.3 (CH_2), 50.8 (CH_2), 64.7 (C_{quat}), 103.2 (CH), 114.6 (CH), 116.1 (C_{quat}), 117.2 (C_{quat}), 128.0 (CH), 129.3 (CH), 129.8 (CH), 137.1 (C_{quat}), 158.7 (C_{quat}), 159.6 (C_{quat}), 167.3 (C_{quat}). EI-MS (70 eV, *m*/*z* (%)): 344 (17), 343 (68, [M^+]), 342 (10), 329 (10), 328 (42, [$C_{22}H_{22}N_3^+$]), 238 (23), 197 (18, [$C_{14}H_{15}N^+$]), 196 (100, [$C_{13}H_{12}N_2^+$]), 167 (16), 130 (13), 104 (12), 71 (11), 70 (11, [$C_4H_8N^+$]). IR: $\tilde{\nu}$ [cm^{-1}]: 2957 (w), 2920 (w), 2868 (w), 2195 (m), 1558 (w), 1514 (m), 1483 (m), 1460 (m), 1433 (s), 1396 (m), 1364 (s), 1329 (m), 1279 (s), 1267 (s), 1225 (s), 1177 (m), 1150 (s), 1121 (m), 1105 (m), 1072 (m), 1007 (m), 995 (m), 974 (m), 951 (m), 934 (m), 903 (m), 887 (m), 862 (m), 851 (m), 810 (m), 768 (m), 706 (m), 691 (m), 675 (m), 644 (m), 604 (m). UV/Vis (CH_2Cl_2): λ_{max} [nm] (ε [$Lcm^{-1}mol^{-1}$]): 510 (77700). Emission (CH_2Cl_2): λ_{max} [nm]: 550, Stokes shift [cm^{-1}]: 1400. Anal. calcd. for $C_{23}H_{25}N_3$ (343.2): C 80.43, H 7.34, N 12.23; Found: C 80.42, H 7.61, N 12.25.

4.4. (*E*)-2-{5,5-Dimethyl-3-[2-(piperidin-1-yl)vinyl]cyclohex-2-en-1-ylidene}malononitrile (6c)

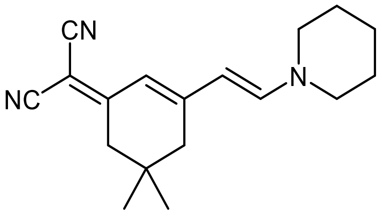

6c

$C_{18}H_{23}N_3$ [281.40]

According to the GP and after flash chromatography on silica gel (*n*-hexane/ethyl acetate 10:1) compound **6c** (85 mg, 60%) was obtained as a red solid, Mp 128 °C. R_f (*n*-hexane/ethyl acetate 5:1): 0.70. ^{1}H NMR (600 MHz, $CDCl_3$): δ 1.00 (s, 6 H), 1.74-1.53 (m, 6 H), 2.26 (s, 2 H), 2.45 (s, 2 H), 3.33 (t, *J* = 5.3 Hz, 4 H), 5.38 (d, *J* = 13.1 Hz, 1 H), 6.34 (s, 1 H), 7.01 (d, *J* = 12.9 Hz, 1 H). ^{13}C NMR (151 MHz, $CDCl_3$): δ 24.1 (CH_2), 25.8 (CH_2), 28.2 (CH_3), 32.0 (C_{quat}), 40.0 (CH_2), 42.9 (CH_2), 51.1 (CH_2), 65.7 (C_{quat}), 100.4 (CH), 112.90 (CH), 116.0 (C_{quat}), 116.8 (C_{quat}), 147.7 (CH), 159.1 (C_{quat}), 167.8 (C_{quat}). EI-MS (70 eV, *m*/*z* (%)): 282 (22), 281 (100, [M^+]), 280 (19), 266 (33, [$C_{17}H_{20}N_3^+$]), 176 (19), 149 (16, [$C_{10}H_{15}N^+$]), 141 (12), 134 (37), 122 (10, [$C_8H_{12}N^+$]), 84 (31, [$C_5H_{10}N^+$]), 83 (34), 55 (11). IR: $\tilde{\nu}$ [cm^{-1}]: 2966 (s), 2943 (w), 2191 (s), 2172 (w), 1616 (m), 1491 (s), 1466 (m), 1443 (m), 1437 (s), 1356 (m), 1298 (w), 1431 (m), 1356 (m), 1298 (w), 1190 (s), 1151 (s), 1277 (s), 1190 (s), 1151 (s), 1001 (m), 960 (m), 1128 (m), 1001 (m), 960

(m), 804 (m), 791 (m), 745 (m). UV/Vis (CH_2Cl_2): λ_{max} [nm] (ε [$Lcm^{-1}mol^{-1}$]): 213 (5500), 490 (72600). Emission (CH_2Cl_2): λ_{max} [nm]: 512, Stokes shift [cm^{-1}]: 860. HR-MS calcd. for $[C_{18}H_{23}N_3+H]^+$: 282.1965; Found: 282.1966.

4.5. (*E*)-2-[5,5-Dimethyl-3-(2-morpholino-2-phenylvinyl)cyclohex-2-en-1-ylidene]malononitrile (6d)

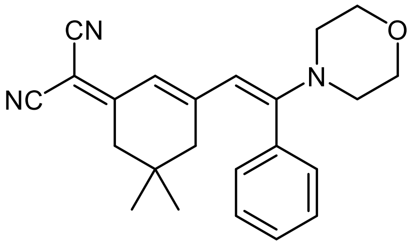

6d

$C_{23}H_{25}N_3O$ [359.47]

According to the GP and after flash chromatography on silica gel (*n*-hexane/ethyl acetate 10:1) compound **6d** (130 mg, 72%) was obtained as a red solid, Mp 159 °C. R_f (*n*-hexane/ethyl acetate 10:1): 0.11. ^{1}H NMR (600 MHz, $CDCl_3$): δ 0.77 (s, 6 H), 1.66 (s, 2 H), 2.30 (s, 2 H), 3.16 (t, J = 4.8 Hz, 4 H), 3.72 (t, J = 4.8 Hz, 4 H), 5.40 (s, 1 H), 6.14 (s, 1 H), 7.20-7.25 (m, 2 H), 7.43 (dd, J = 8.4, 6.9 Hz, 2 H), 7.47-7.51 (m, 1 H). ^{13}C NMR (151 MHz, $CDCl_3$): δ 27.7 (CH_3), 32.1 (C_{quat}), 42.4 (CH_2), 44.2 (CH_2), 48.9 (CH_2), 66.7 (CH_2), 69.9 (C_{quat}), 105.7 (CH), 114.5 (C_{quat}), 115.7 (C_{quat}), 118.0 (CH), 129.3 (CH), 129.6 (CH), 130.3 (CH), 136.1 (C_{quat}), 158.7 (C_{quat}), 159.9 (C_{quat}), 168.3 (C_{quat}). EI-MS (70 eV, *m*/*z* (%)): 360 (26), 359 (100, [M^+]), 358 (36), 344 (18, [$C_{22}H_{22}N_3O^+$]), 254 (28), 212 (44), 115 (10), 105 (26), 104 (11), 103 (10, [$C_6H_3N_2^+$]), 91 (14), 77 (11, [$C_6H_5^+$]). IR: $\tilde{\nu}$ [cm^{-1}]: 2953 (w), 2864 (w), 2199 (m), 1522 (m), 1474 (s), 1462 (s), 1443 (m), 1422 (m), 1389 (m), 1368 (m), 1352 (m), 1333 (w), 1302 (s), 1288 (s), 1263 (m), 1244 (s), 1221 (s), 1209 (s), 1186 (m), 1152 (s), 1109 (s), 1063 (m), 1032 (m), 1022 (m), 1007 (m), 991 (m), 978 (m), 922 (m), 905 (m), 880 (s), 870 (m), 856 (m), 808 (m), 777 (s), 770 (s), 731 (m), 704 (m), 679 (m), 654 (s). UV/Vis (CH_2Cl_2): λ_{max} [nm] (ε [$Lcm^{-1}mol^{-1}$]): 284 (8400), 479 (46600). Emission (CH_2Cl_2): λ_{max} [nm]: 562, Stokes shift [cm^{-1}]: 3100. Anal. calcd. for $C_{23}H_{25}N_3O$ (359.2): C 76.85, H 7.01, N 11.69; Found: C 76.57, H 7.27, N 11.60.

4.6. (*E*)-2-{5,5-Dimethyl-3-[2-phenyl-2-(piperidin-1-yl)vinyl]cyclohex-2-en-1-ylidene}malononitrile (6e)

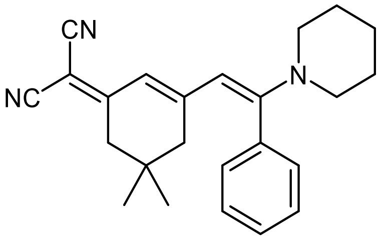

6e

$C_{24}H_{27}N_3$ [357.50]

According to the GP and after flash chromatography on silica gel (*n*-hexane/ethyl acetate 10:1) compound **6e** (178 mg, 80%) was obtained as a red solid, Mp 157 °C. R_f (*n*-hexane/ethyl acetate 10:1): 0.11. ^{1}H NMR (600 MHz, $CDCl_3$): δ 0.76 (s, 6 H), 1.61 (s, 6 H), 1.68 (q, *J* = 6.5, 3.1 Hz, 2 H), 2.28 (s, 2 H), 3.23 (s, 4 H), 5.44 (s, 1 H), 6.05 (s, 1 H), 7.22 (d, *J* = 7.4 Hz, 2 H), 7.43 (t, *J* = 7.6 Hz, 2 H), 7.54-7.47 (m, 1 H). ^{13}C NMR (151 MHz, $CDCl_3$): δ 24.4 (CH_2), 26.1 (CH_2), 27.8 (CH_3), 32.0 (C_{quat}), 42.4 (CH_2), 44.2 (CH_2), 50.1 (CH_2), 66.6 (C_{quat}), 104.6 (CH), 115.5 (C_{quat}), 116.2 (CH), 116.6 (C_{quat}), 129.2 (CH), 129.3 (CH), 130.2 (CH), 136.8 (C_{quat}), 159.9 (C_{quat}), 161.0 (C_{quat}), 167.6 (C_{quat}). EI-MS (70 eV, *m*/*z* (%)): 357 (3, [M^+]), 342 (1, [$C_{23}H_{24}N_3^+$]), 252 (2), 210 (4), 188 (6), 171 (4, [$C_{11}H_{11}N_2^+$]), 132 (5), 106 (8), 105 (100), 77 (23, [$C_6H_5^+$]). IR: $\tilde{\nu}$ [cm^{-1}]: 3007 (w), 2934 (w), 2864 (w), 2201 (m), 2180 (w), 1512 (m), 1460 (s), 1439 (s), 1402 (m), 1387 (m), 1366 (m), 1344 (s), 1333 (m), 1290 (m), 1223 (s), 1209 (s), 1196 (s), 1171 (s), 1144 (s), 1121 (m), 1096 (m), 1070 (m), 1016 (s), 993 (s), 952 (m), 905 (s), 880 (m), 868 (m), 851 (m), 837 (m), 814 (m), 785 (m), 762 (m), 721 (m), 700 (m), 673 (m), 642 (m), 606 (m). UV/Vis (CH_2Cl_2): λ_{max} [nm] (ε [$Lcm^{-1}mol^{-1}$]): 262 (9900), 508 (71700). Emission (CH_2Cl_2): λ_{max} [nm]: 576, Stokes shift [cm^{-1}]: 2300. Anal. calcd. for $C_{24}H_{27}N_3$ (357.2): C 80.63, H 7.61, N 11.75; Found: C 80.37, H 7.55; N 11.49.

4.7. Methyl-(*E*)-4-{2-[3-(dicyanomethylene)-5,5-dimethylcyclohex-1-en-1-yl]-1-(pyrrolidin-1-yl)vinyl}benzoate (6f)

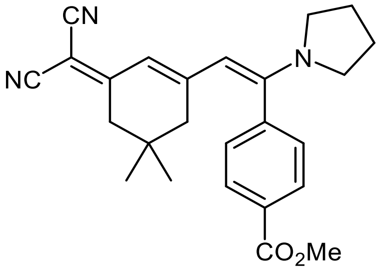

6f

$C_{25}H_{27}N_3O_2$ [401.51]

According to the GP and after flash chromatography on silica gel (*n*-hexane/ethyl acetate 4:1) compound **6f** (170 mg, 85%) was obtained as a red solid, Mp 185 °C. R_f (*n*-hexane/ethyl

acetate 4:1): 0.16. ^{1}H NMR (600 MHz, CDCzl$_3$): δ 0.74 (s, 6 H), 1.62 (s, 2 H), 1.97 (s, 4 H), 2.25 (s, 2 H), 3.09 (s, 2 H), 3.40 (s, 2 H), 3.95 (d, J = 1.3 Hz, 3 H), 5.29 (s, 1 H), 6.03 (s, 1 H), 7.29-7.37 (m, 2 H), 8.13 (dd, J = 8.1, 1.4 Hz, 2 H). ^{13}C NMR (151 MHz, CDCl$_3$): δ 25.4 (CH$_2$), 27.8 (CH$_3$), 31.9 (C$_{quat}$), 41.0 (C$_{quat}$), 42.3 (CH$_2$), 44.2 (CH$_2$), 49.6 (CH$_2$), 52.5 (CH$_3$), 66.1 (C$_{quat}$), 103.1 (CH), 115.0 (CH), 115.6 (C$_{quat}$), 116.7 (C$_{quat}$), 128.3 (CH), 130.5 (CH), 131.4 (C$_{quat}$), 141.6 (C$_{quat}$), 156.9 (C$_{quat}$), 158.5 (C$_{quat}$), 166.4 (C$_{quat}$), 167.5 (C$_{quat}$). EI-MS (70 eV, m/z (%)): 402 (23), 401 (91, [M$^+$]), 400 (11), 387 (11), 386 (42, [$C_{24}H_{24}N_3O_2^+$]), 296 (24), 255 (22, [$C_{16}H_{17}NO_2^+$]), 254 (100), 188 (12), 185 (14), 163 (28), 162 (16), 70 (13, [$C_4H_8N^+$]). IR: $\tilde{\nu}$ [cm^{-1}]: 2203 (m), 2189 (w), 1719 (m), 1566 (w), 1558 (m), 1522 (s), 1506 (m), 1468 (m), 1452 (m), 1443 (m), 1437 (m), 1414 (w), 1400 (m), 1366 (m), 1344 (m), 1325 (m), 1308 (w), 1288 (m), 1275 (m), 1225 (m), 1192 (m), 1177 (m), 1151 (m), 1128 (m), 1111 (m), 1099 (m), 1020 (m), 958 (w), 893 (m), 874 (m), 862 (w), 851 (w), 824 (w), 783 (m), 768 (m), 718 (w), 608 (m). UV/Vis (CH$_2$Cl$_2$): λ_{max} [nm] (ε [Lcm^{-1}mol^{-1}]): 269 (9600), 507 (46000). Emission (CH$_2$Cl$_2$): λ_{max} [nm]: 570, Stokes shift [cm^{-1}]: 2200. Anal. calcd. for $C_{25}H_{27}N_3O_2$ (401.2): $C_{25}H_{27}N_3O_2$ (401.2): C 74.79, H 6.78, N 10.47; Found: C 75.08, H 6.72, N 10.19.

4.8. (*E*)-2-{3-[2-(4-Methoxyphenyl)-2-(pyrrolidin-1-yl)vinyl]-5,5-dimethylcyclohex-2-en-1-ylidene}malononitrile (6g)

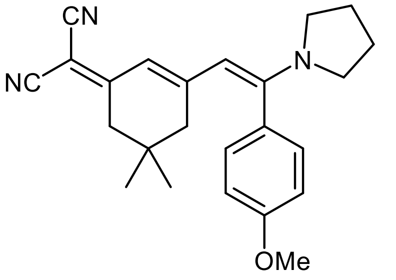

6g

$C_{24}H_{27}N_3O$ [373.50]

According to the GP and after flash chromatography on silica gel (*n*-hexane/ethyl acetate 4:1) compound **6g** (105 mg, 56%) was obtained as a red solid, Mp 190 °C. R_f (*n*-hexane/ethyl acetate 4:1): 0.11. ^{1}H NMR (600 MHz, CDCl$_3$): δ 0.78 (s, 6 H), 1.71 (s, 2 H), 1.86 (s, 2 H), 2.02 (s, 2 H), 2.27 (s, 2 H), 3.15 (s, 2 H), 3.43 (s, 2 H), 3.87 (s, 3 H), 5.25 (s, 1 H), 5.99 (s, 1 H), 6.98 (d, J = 8.7 Hz, 2 H), 7.12 (d, J = 8.6 Hz, 2 H). ^{13}C NMR (151 MHz, CDCl$_3$): δ 25.4 (CH$_2$), 27.9 (CH$_2$), 31.9 (C$_{quat}$), 42.4 (CH$_3$), 44.4 (CH$_2$), 49.2 (CH$_2$), 55.6 (CH$_3$), 64.2 (C$_{quat}$), 103.2 (CH), 114.1 (CH), 114.9 (CH), 116.2 (C$_{quat}$), 117.4 (C$_{quat}$), 129.0 (C$_{quat}$), 129.4 (CH), 156.1 (C$_{quat}$), 159.7 (C$_{quat}$), 161.0 (C$_{quat}$), 167.1 (C$_{quat}$). EI-MS (70 eV, m/z (%)): 374 (12), 373 (43, [M$^+$]), 358 (20, [$C_{23}H_{24}N_3O^+$]), 226 (33), 136 (10), 135 (100). IR: $\tilde{\nu}$ [cm^{-1}]: 2195 (m), 1609 (w), 1508 (m), 1483 (w), 1466 (m), 1460 (m), 1431 (s), 1425 (s), 1420 (m), 1396 (m), 1364 (m), 1340 (m), 1323 (m), 1281 (s), 1248 (m), 1209 (s), 1182 (m), 1173 (m), 1144 (s), 1105 (m),

1030 (w), 1024 (w), 1007 (w), 993 (w), 922 (w), 893 (w), 872 (m), 839 (m), 800 (m), 739 (w), 673 (w), 615 (w). UV/Vis (CH_2Cl_2): λ_{max} [nm] (ε [$Lcm^{-1}mol^{-1}$]): 300 (10800), 513 (56800). Emission (CH_2Cl_2): λ_{max} [nm]: 559, Stokes shift [cm^{-1}]: 1600. HR-MS calcd. for $[C_{24}H_{27}N_3O+H]^+$: 374.2227; Found: 374.2225.

4.9. (*E*)-2-(3-{2-[(4-Methoxyphenyl)amino]-2-phenylvinyl}-5,5-dimethylcyclohex-2-en-1-ylidene)malononitrile (6h)

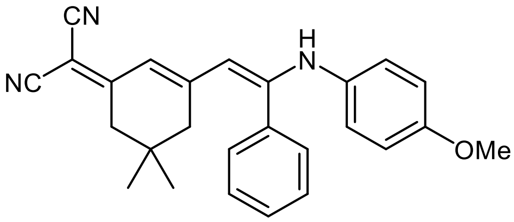

6h

$C_{26}H_{25}N_3O$ [395.51]

According to the GP and after flash chromatography on silica gel (*n*-hexane/ethyl acetate 5:1) compound **6h** (150 mg, 76%) was obtained as a red solid, R_f (*n*-hexane/ethyl acetate 5:1): 0.09. ^{1}H NMR (600 MHz, DMSO-d$_6$): δ 0.83 (s, 6 H), 2.00 (s, 2 H), 2.32 (s, 2 H), 3.76 (s, 3 H), 5.68 (s, 1 H), 6.04 (s, 1 H), 6.97-6.80 (m, 2 H), 7.10 (s, 2 H), 7.47 (d, *J* = 4.4 Hz, 4 H), 7.53 (dt, *J* = 8.2, 4.1 Hz, 1 H), 9.13 (s, 1 H). ^{13}C NMR (151 MHz, $CDCl_3$): δ 26.9 (CH_3), 31.1 (C_{quat}), 40.1 (C_{quat}), 41.6 (CH_2), 43.5 (CH_2), 55.1 (CH_3), 65.4 (C_{quat}), 104.2 (CH), 113.6 (C_{quat}), 114.2 (CH), 114.5 (CH), 123.8 (CH), 127.1 (C_{quat}), 127.9 (C_{quat}), 128.3 (CH), 128.7 (CH), 129.8 (CH), 137.5 (C_{quat}), 156.5 (C_{quat}), 159.7 (C_{quat}), 166.7 (C_{quat}). EI-MS (70 eV, *m*/*z* (%)): 395 (4, [M^+]), 380 (1), 288 (6), 227 (11), 211 (16), 210 (100), 123 (12), 108 (11), 105 (66), 77 (30). IR: $\tilde{\nu}$ [cm^{-1}]: 2959 (w), 2872 (w), 2837 (w), 2228 (w), 1713 (w), 1663 (m), 1605 (m), 1574 (w), 1553 (w), 1539 (w), 1501 (s), 1464 (w), 1447 (w), 1414 (w), 1387 (w), 1371 (w), 1317 (w), 1292 (m), 1244 (s), 1217 (m), 1194 (m), 1179 (m), 1153 (m), 1130 (w), 845 (m), 880 (w), 895 (w), 937 (w), 980 (w), 1003 (w), 1032 (m), 1074 (w), 1107 (w), 833 (m), 810 (w), 783 (w), 770 (w), 752 (w), 716 (m), 692 (m), 654 (w), 640 (w), 621 (w). UV/Vis (CH_2Cl_2): λ_{max} [nm] (ε [$Lcm^{-1}mol^{-1}$]): 493 (42100). Emission (CH_2Cl_2): λ_{max} [nm]: 623, Stokes shift [cm^{-1}]: 4200. Anal. calcd. for $C_{26}H_{25}N_3O$ (395.5): C 78.96, H 6.37, N 10.62; Found: C 78.69, H 6.44, N 10.39.

4.10. 2,2'-{[(1*E*,1'*E*)-Piperazin-1,4-diylbis(2-phenylethen-2,1-diyl)]bis(5,5-dimethylcyclohex-2-en-3-yl-1-ylidene)}dimalononitrile (6i)

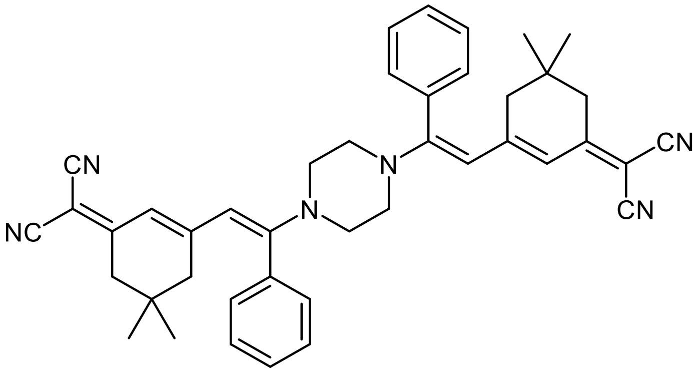

6i

$C_{42}H_{42}N_6$ [630.84]

According to the GP and after flash chromatography on silica gel (*n*-hexane/ethyl acetate 4:1) compound **6i** (77 mg, 24%) was obtained as a red solid, Mp 267 °C. R_f (*n*-hexane/ethyl acetate 4:1: 0.14. ^{1}H NMR (600 MHz, $CDCl_3$): δ 0.77 (s, 12 H), 1.66 (s, 4 H), 2.31 (s, 4 H), 3.23 (s, 8 H), 5.41 (s, 2 H), 6.17 (s, 2 H), 7.23 (dt, *J* = 6.9, 1.4 Hz, 4 H), 7.42 (t, *J* = 7.5 Hz, 4 H), 7.46-7.54 (m, 2 H). ^{13}C NMR (151 MHz, $CDCl_3$): δ 27.7 (CH_3), 32.2 (C_{quat}), 42.4 (CH_2), 44.1 (CH_2), 48.1 (CH_2), 70.9 (C_{quat}), 106.3 (CH), 114.3 (C_{quat}), 115.4 (C_{quat}), 118.6 (CH), 129.4 (CH), 129.5 (CH), 130.5 (CH), 136.0 (C_{quat}), 158.1 (C_{quat}), 158.9 (C_{quat}), 168.4 (C_{quat}). EI-MS (70 eV, *m*/*z* (%)): 632 (10), 631 (38), 630 (78, [M^+]), 357 (15, [$C_{23}H_{25}N_4^+$]), 329 (32, [$C_{22}H_{23}N_3^+$]), 328 (99), 300 (100), 273 (20, [$C_{19}H_{17}N_2^+$]), 244 (19), 231 (16), 209 (27, [$C_{16}H_{17}^+$]), 170 (23), 130 (78), 104 (28), 91 (44), 77 (11, [$C_6H_5^+$]). IR: $\tilde{\nu}$ [cm^{-1}]: 2961 (w), 2924 (w), 2866 (w), 2201 (m), 1506 (m), 1464 (m), 1418 (m), 1398 (m), 1375 (m), 1346 (m), 1279 (m), 1265 (w), 1233 (m), 1211 (s), 1184 (s), 1148 (s), 1119 (s), 1078 (m), 1028 (m), 1042 (m), 1003 (m), 988 (m), 980 (m), 932 (m), 922 (m), 893 (m), 874 (s), 866 (w), 837 (w), 812 (m), 768 (s), 729 (m), 702 (m), 681 (m), 654 (s), 602 (m). UV/Vis (CH_2Cl_2): λ_{max} [nm] (ε [$Lcm^{-1}mol^{-1}$]): 282 (14700), 498 (71100). Emission (CH_2Cl_2): λ_{max} [nm]: 576, Stokes shift [cm^{-1}]: 2700. HR-MS calcd. for [$C_{42}H_{42}N_6$+H]$^+$: 631.3544; Found: 631.3543.

4.11. 2-{5,5-Dimethyl-3-[3-(1,3,3-trimethylindolin-2-ylidene)-2-(trimethylsilyl)prop-1-en-1-yl]cyclohex-2-en-1-ylidene}malononitrile (8a)

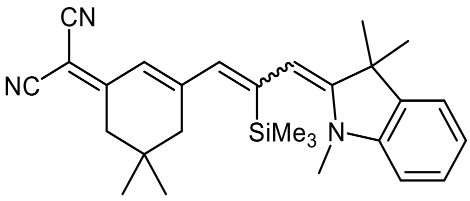

8a

$C_{28}H_{35}N_3Si$ [441.69]

According to the GP and after flash chromatography on silica gel (*n*-hexane/ethyl acetate 10:1) compound **8a** (140 mg, 63%) was obtained as a red solid, Mp 119 °C. R_f (*n*-hexane/ethyl acetate 10:1): 0.34. ^{1}H NMR (600 MHz, DMSO-d$_6$): δ 0.14 (s, 9 H), 0.96 (s, 6 H), 1.40 (s, 6 H), 2.22 (s, 2 H), 2.45 (s, 2 H), 3.30 (s, 3 H), 5.79 (d, *J* = 2.8 Hz, 1 H), 6.00 (d, *J* = 2.9 Hz, 1 H), 6.09 (s, 1 H), 7.12 (dt, *J* = 7.5, 3.4 Hz, 2 H), 7.28-7.36 (m, 1 H), 7.40 (d, *J* = 7.4 Hz, 1 H). ^{13}C NMR (151 MHz, DMSO-d$_6$): δ 0.2 (CH_3), 27.5 (CH_3), 28.8 (CH_3), 31.9 (C_{quat}), 38.1 (CH_3), 40.1 (C_{quat}), 42.1 (CH_2), 43.1 (CH_2), 49.6 (C_{quat}), 63.4 (C_{quat}), 110.5 (CH), 115.8 (CH), 115.9 (C_{quat}), 116.5 (C_{quat}), 121.8 (CH), 123.3 (CH), 128.0 (CH), 135.1 (2xCH), 140.2 (C_{quat}), 144.2 (C_{quat}), 150.9 (C_{quat}), 158.8 (C_{quat}), 165.8 (C_{quat}). EI-MS (70 eV, *m*/*z* (%)): 441 (7, [M^+]), 368 (2, [$C_{25}H_{26}N_3^+$]), 253 (13), 174 (13), 173 (100), 159 (22, [$C_{11}H_{13}N^+$]), 158 (91, [$C_{11}H_{12}N^+$]), 73 (9, [$C_3H_9Si^+$]). IR: $\tilde{\nu}$ [cm^{-1}]: 2965 (w), 2953 (w), 2932 (w), 2895 (w), 2220 (w), 1603 (w), 1578 (m), 1522 (m), 1489 (m), 1476 (m), 1460 (m), 1412 (w), 1383 (w), 1367 (w), 1319 (m), 1300 (m), 1283 (w), 1248 (m), 1229 (m), 1184 (w), 1155 (w), 1113 (m), 1076 (m), 1047 (m), 1020 (m), 986 (w), 957 (w), 945 (m), 918 (w), 894 (w), 853 (m), 835 (s), 802 (m), 756 (m), 735 (s), 716 (w), 683 (w), 667 (m), 625 (w). UV/Vis (CH_2Cl_2): λ_{max} [nm] (ε [Lcm^{-1}mol^{-1}]): 318 (25700), 549 (32600). Emission (CH_2Cl_2): λ_{max} [nm]: 695, Stokes shift [cm^{-1}]: 3800. Anal. calcd. for $C_{28}H_{35}N_3Si$ (441.3): C 76.14, H 7.99; N 9.51; Found: C 75.93, H 8.29, N 9.30.

4.12. 2-{5,5-Dimethyl-3-[2-phenyl-3-(1,3,3-trimethylindolin-2-ylidene)prop-1-en-1-yl]cyclohex-2-en-1-ylidene}malononitrile (8b)

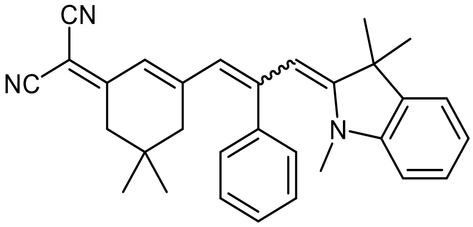

8b

$C_{31}H_{31}N_3$ [445.61]

According to the GP and after flash chromatography on silica gel (*n*-hexane/ethyl acetate 10:1) compound **8b** (178 mg, 80%) was obtained as a red solid, Mp 104 °C. R_f (*n*-hexane/ethyl acetate 10:1): 0.25. ^{1}H NMR (600 MHz, 353 K, DMSO-d$_6$): δ 0.77 (s, 6 H), 1.39 (s, 6 H), 2.24 (s, 2 H), 2.48 (s, 2 H), 3.14 (s, 3 H), 5.23 (d, *J* = 1.4 Hz, 1 H), 6.13 (d, *J* = 1.4 Hz, 1 H), 6.18 (s, 1 H), 6.96 (d, *J* = 7.9 Hz, 1 H), 7.07-7.01 (m, 1 H), 7.28-7.24 (m, 2 H), 7.29 (dd, *J* = 7.5, 1.1 Hz, 1 H), 7.33 (dd, *J* = 8.4, 6.9 Hz, 2 H), 7.55-7.46 (m, 2 H). ^{13}C NMR (151 MHz, 353 K, $CDCl_3$): δ 26.7 (CH_3), 28.6 (CH_3), 31.0 (C_{quat}), 36.8 (CH_3), 40.1 (CH), 41.8 (CH_2), 43.0 (CH_2), 48.8 (C_{quat}), 108.5 (CH), 111.0 (C_{quat}), 114.0 (C_{quat}), 114.8 (C_{quat}), 118.6 (CH), 119.5 (CH), 121.1 (CH), 121.9 (CH), 126.4 (CH), 127.4 (CH), 127.5 (CH), 128.0 (CH), 139.4 (C_{quat}), 140.2 (C_{quat}), 144.0 (C_{quat}), 144.3 (C_{quat}), 160.3 (C_{quat}), 165.8 (C_{quat}), 168.0 (C_{quat}). EI-MS (70 eV, *m*/*z* (%)): 445 (13, [M^+]), 260 (10, [$C_{19}H_{17}N^+$]), 174 (12), 173 (85), 159 (43, [$C_{11}H_{13}N^+$]), 158 (100,

$[C_{11}H_{12}N^+]$), 145 (10, $[C_{10}H_{11}N^+]$), 144 (16, $[C_{10}H_{10}N^+]$). IR: $\tilde{\nu}$ [cm^{-1}]: 3053 (w), 2959 (w), 2928 (w), 2899 (w), 2208 (m), 1602 (w), 1485 (s), 1456 (s), 1443 (s), 1410 (m), 1381 (w), 1362 (w), 1317 (m), 1300 (m), 1285 (m), 1209 (m), 1184 (m), 1152 (m), 1125 (s), 1072 (m), 1042 (w), 1020 (m), 984 (w), 955 (w), 918 (m), 878 (w), 845 (w), 824 (w), 804 (w), 781 (m), 745 (m), 700 (s), 656 (w). UV/Vis (CH_2Cl_2): λ_{max} [nm] (ε [Lcm^{-1}mol^{-1}]): 201 (17000), 296 (19900), 537 (21400). Emission (CH_2Cl_2): λ_{max} [nm]: 633, Stokes shift [cm^{-1}]: 2800. Anal. calcd. for $C_{31}H_{31}N_3$ (445.3): C 83.56, H 7.01, N 9.43; Found: C 83.28, H 7.00, N 9.14.

4.13. 2-{3-[2-(4-Methoxyphenyl)-3-(1,3,3-trimethylindolin-2-ylidene)prop-1-en-1-yl]-5,5-dimethylcyclohex-2-en-1-ylidene}malononitrile (8c)

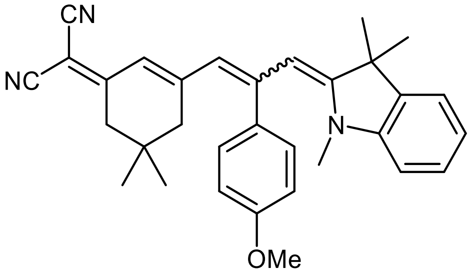

8c

$C_{32}H_{33}N_3O$ [475.64]

According to the GP and after flash chromatography on silica gel (*n*-hexane/ethyl acetate 5:1) compound **8c** (182 mg, 76%) was obtained as a red solid, Mp 114 °C. R_f (*n*-hexane/ethyl acetate 5:1): 0.20. ^{1}H NMR (600 MHz, 353 K, DMSO-d$_6$): δ 0.79 (s, 6 H), 1.38 (s, 6 H), 2.25 (s, 2 H), 2.48 (s, 2 H), 3.13 (s, 3 H), 3.74 (s, 3 H), 5.09 (d, J = 1.4 Hz, 1 H), 5.66 (s, 1 H), 5.99 (d, J = 1.4 Hz, 1 H), 6.19 (s, 1 H), 6.92-6.86 (m, 2 H), 6.94 (d, J = 7.9 Hz, 1 H), 7.02 (t, J = 7.4 Hz, 1 H), 7.30-7.22 (m, 2 H), 7.44 (d, J = 8.8 Hz, 2 H). ^{13}C NMR (151 MHz, 353 K, DMSO-d$_6$): δ 26.9 (CH_3), 28.6 (CH_3), 31.1 (C_{quat}), 36.9 (CH_3), 40.1 (CH), 42.0 (CH_2), 43.0 (CH_2), 48.9 (C_{quat}), 54.4 (C_{quat}), 54.9 (CH_3), 108.7 (CH), 111.4 (C_{quat}), 113.7 (CH), 114.2 (C_{quat}), 115.0 (C_{quat}), 117.6 (CH), 118.4 (CH), 121.2 (CH), 122.1 (CH), 127.6 (CH), 127.7 (CH), 132.9 (C_{quat}), 139.6 (C_{quat}), 143.4 (C_{quat}), 144.4 (C_{quat}), 159.0 (C_{quat}), 160.6 (C_{quat}), 168.0 (C_{quat}). EI-MS (70 eV, *m/z* (%)): 476 (11), 475 (31, [M$^+$]), 460 (15), 290 (17), 174 (14), 173 (100), 159 (30), 158 (93). IR: $\tilde{\nu}$ [cm^{-1}]: 2970 (w), 2928 (w), 2868 (w), 2839 (w), 2210 (w), 2200 (w), 1601 (w), 1557 (w), 1528 (m), 1508 (m), 1464 (m), 1443 (m), 1412 (m), 1344 (w), 1315 (m), 1300 (m), 1281 (m), 1242 (m), 1209 (m), 1182 (m), 1146 (m), 1130 (m), 1117 (m), 1099 (w), 1074 (m), 1043 (w), 1022 (m), 982 (w), 949 (w), 922 (m), 903 (m), 876 (m), 802 (w), 748 (s), 712 (m), 702 (m), 671 (w), 652 (m), 621 (m). UV/Vis (CH_2Cl_2): λ_{max} [nm] (ε [Lcm^{-1}mol^{-1}]): 275 (28200), 539 (27700). Emission (CH_2Cl_2): λ_{max} [nm]: 647, Stokes shift [cm^{-1}]: 3100. HR-MS calcd. for $[C_{32}H_{33}N_3O+H]^+$: 476.2696; Found: 476.2698.

5. NMR Spectra of Compound 3 and Merocyanines 4 and 6

5.1. 3-(Dicyanomethylene)-5,5-dimethylcyclohex-1-en-1-yl trifluoromethanesulfonate (3)

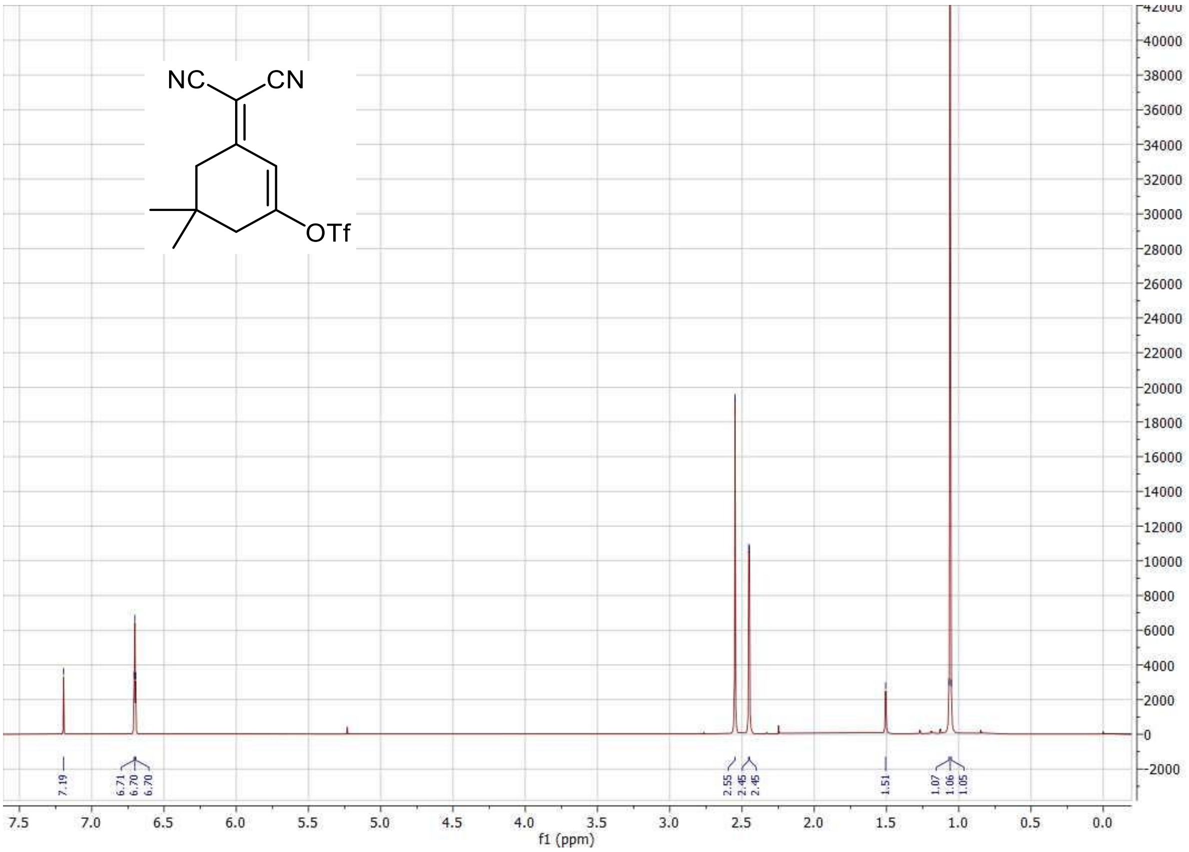

Figure S1. ^{1}H NMR spectrum of compound **3** ($CDCl_3$, 300 MHz, 293 K).

5.2. (*E*)-2-{5,5-Dimethyl-3-[2-(pyrrolidin-1-yl)vinyl]cyclohex-2-en-1-ylidene}malononitrile (6a)

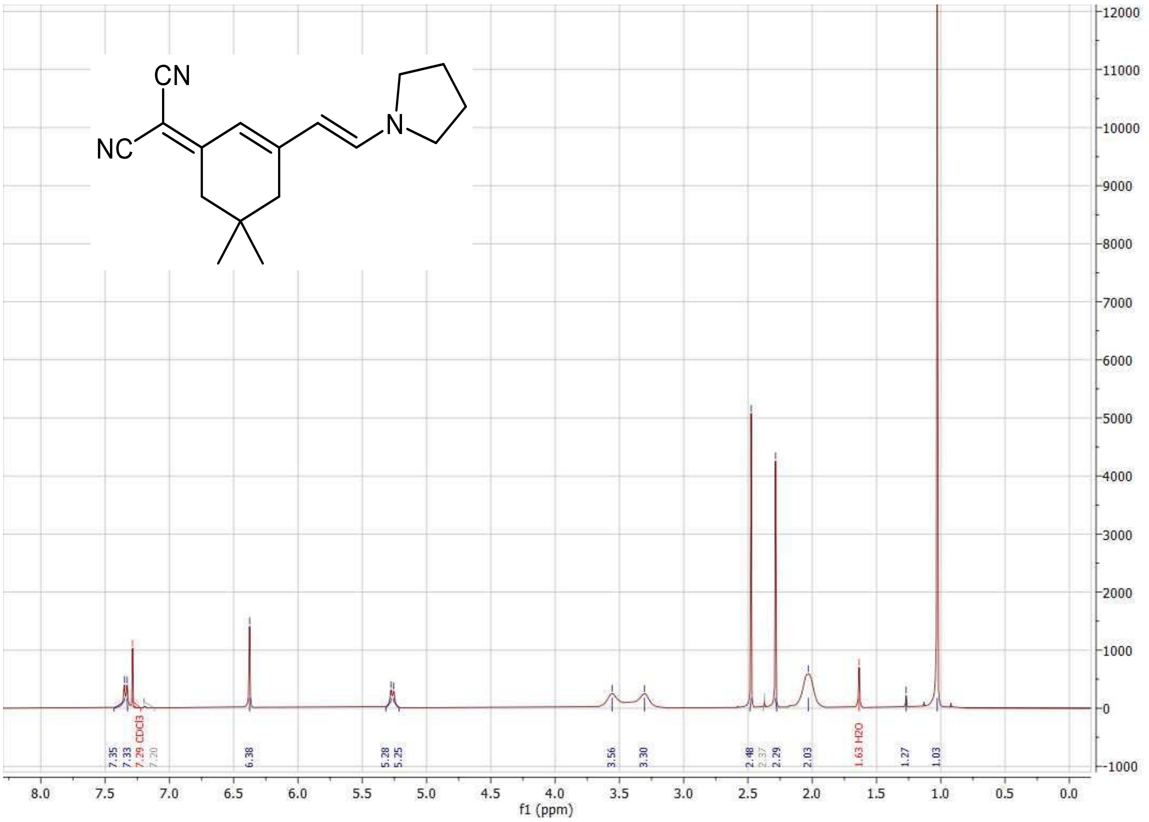

Figure S2. ^{1}H NMR spectrum of compound **6a** ($CDCl_3$, 600 MHz, 293 K).

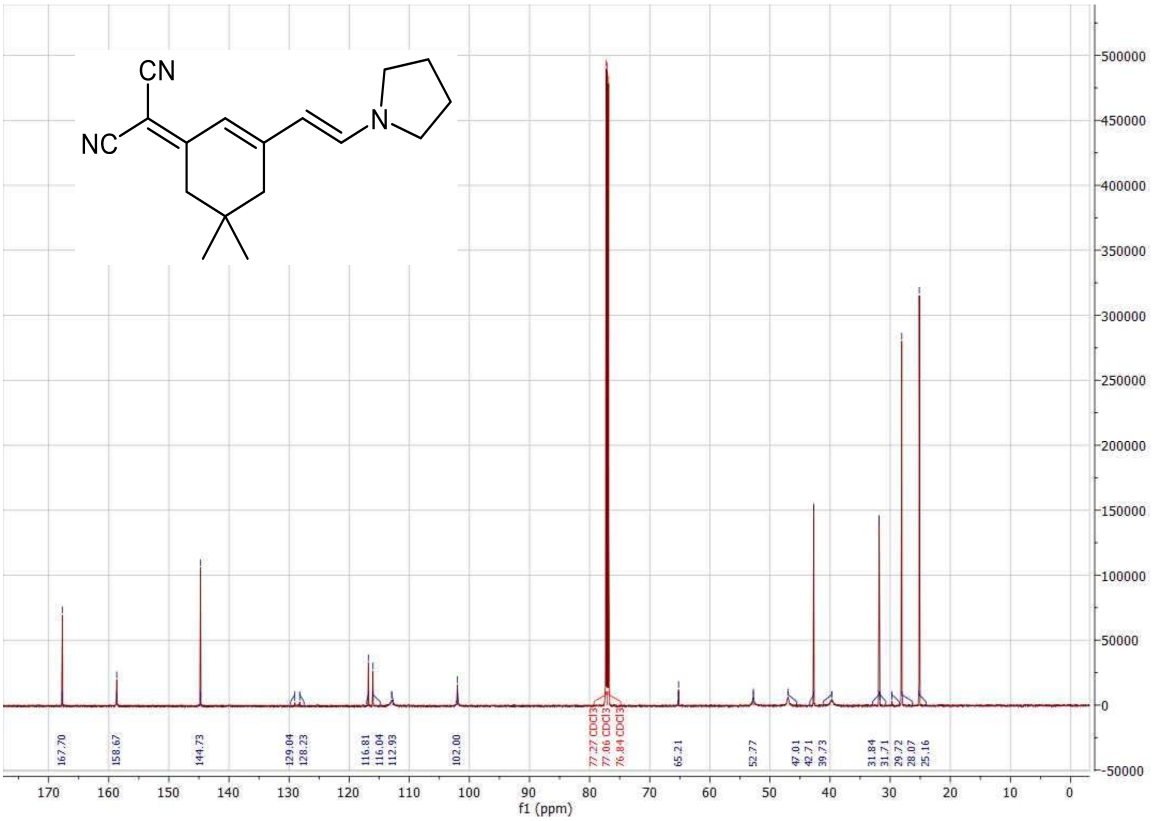

Figure S3. ^{13}C NMR spectrum of compound **6a** ($CDCl_3$, 151 MHz, 293 K).

5.3. (*E*)-2-{5,5-Dimethyl-3-[2-phenyl-2-(pyrrolidin-1-yl)vinyl]cyclohex-2-en-1-ylidene}malononitrile (6b)

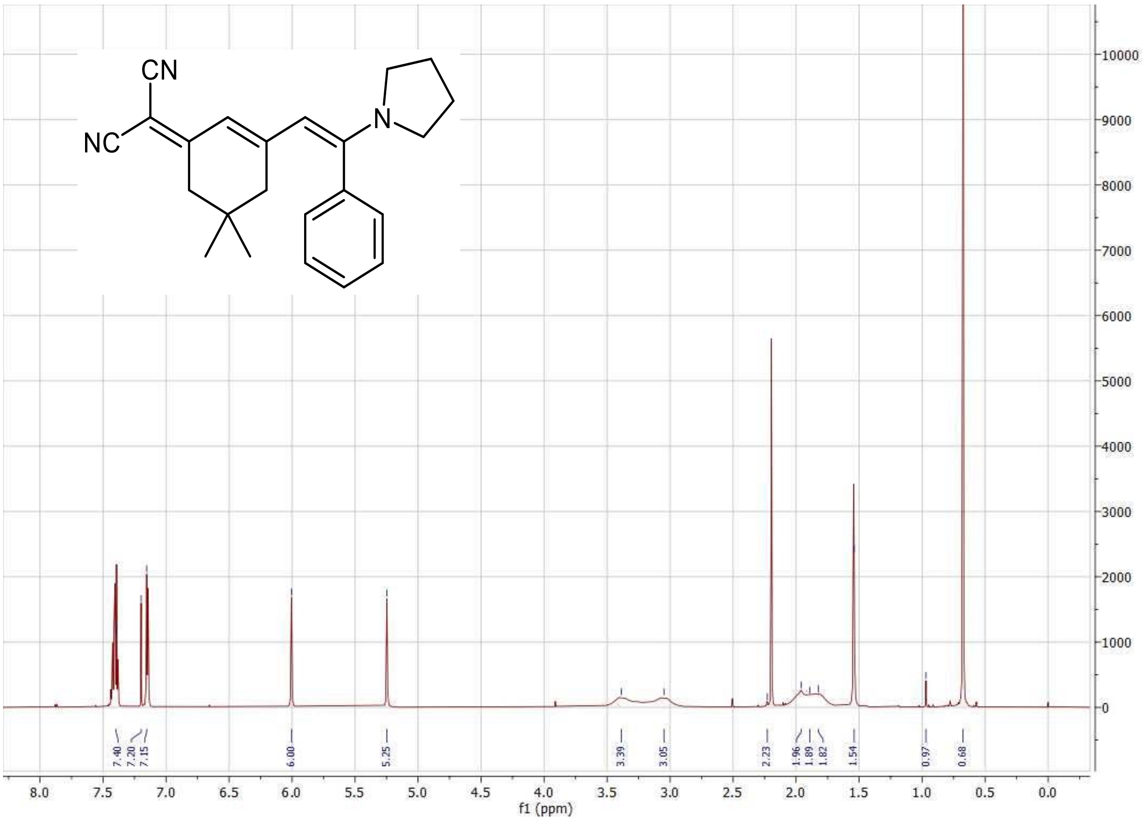

Figure S4. ^{1}H NMR spectrum of compound **6b** ($CDCl_3$, 600 MHz, 293 K).

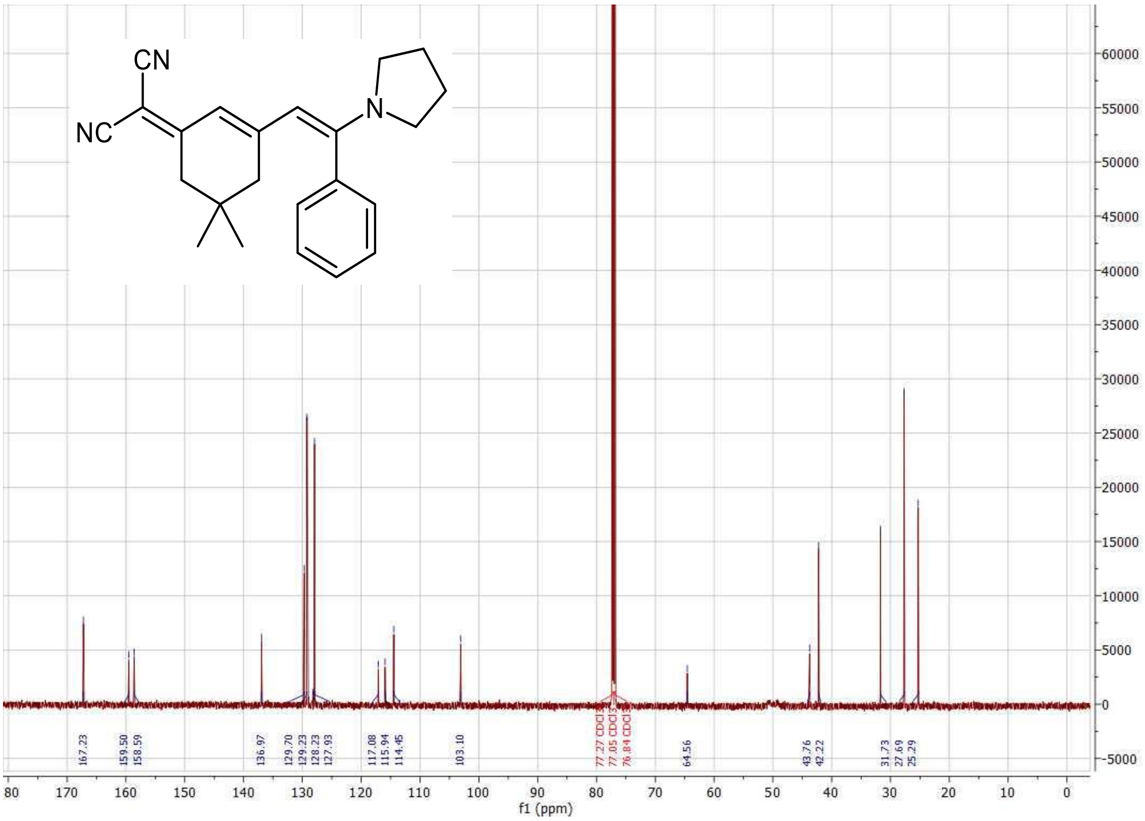

Figure S5. ^{13}C NMR spectrum of compound **6b** ($CDCl_3$, 151 MHz, 293 K).

5.4. (*E*)-2-{5,5-Dimethyl-3-[2-(piperidin-1-yl)vinyl]cyclohex-2-en-1-ylidene}malononitrile (6c)

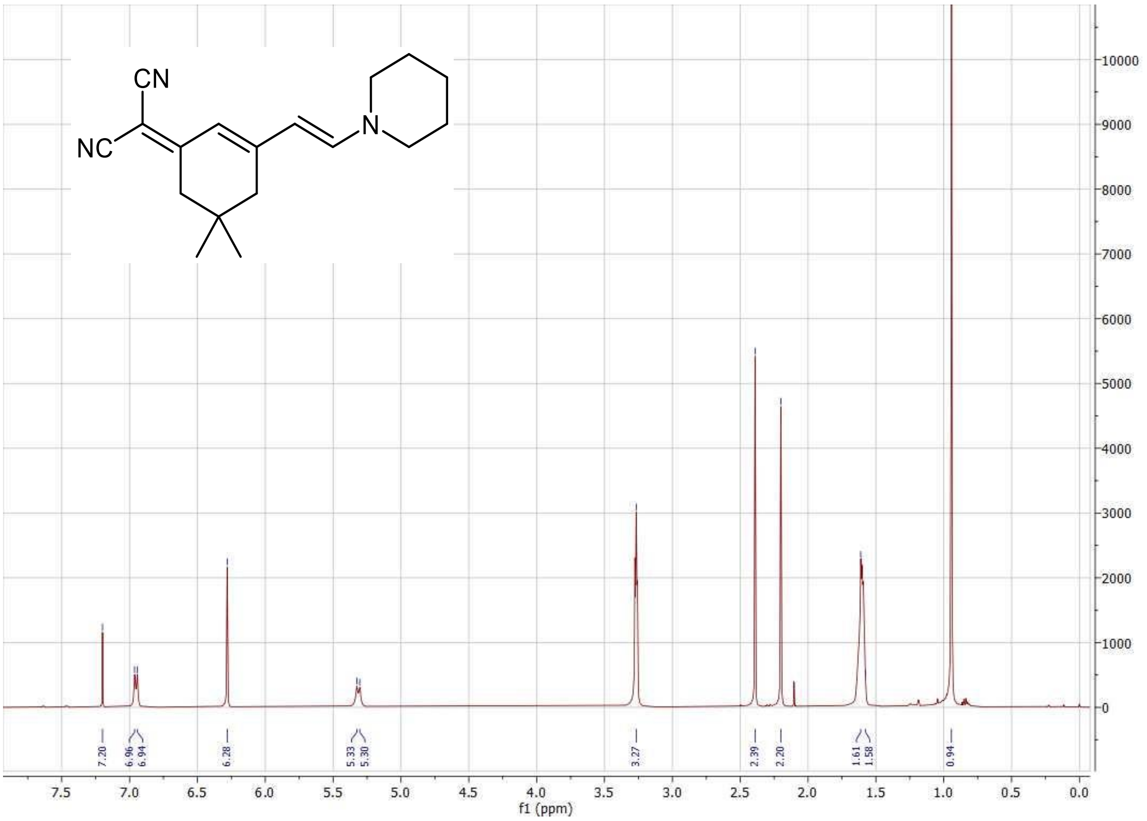

Figure S6. ^1H NMR spectrum of compound **6c** ($CDCl_3$, 600 MHz, 293 K).

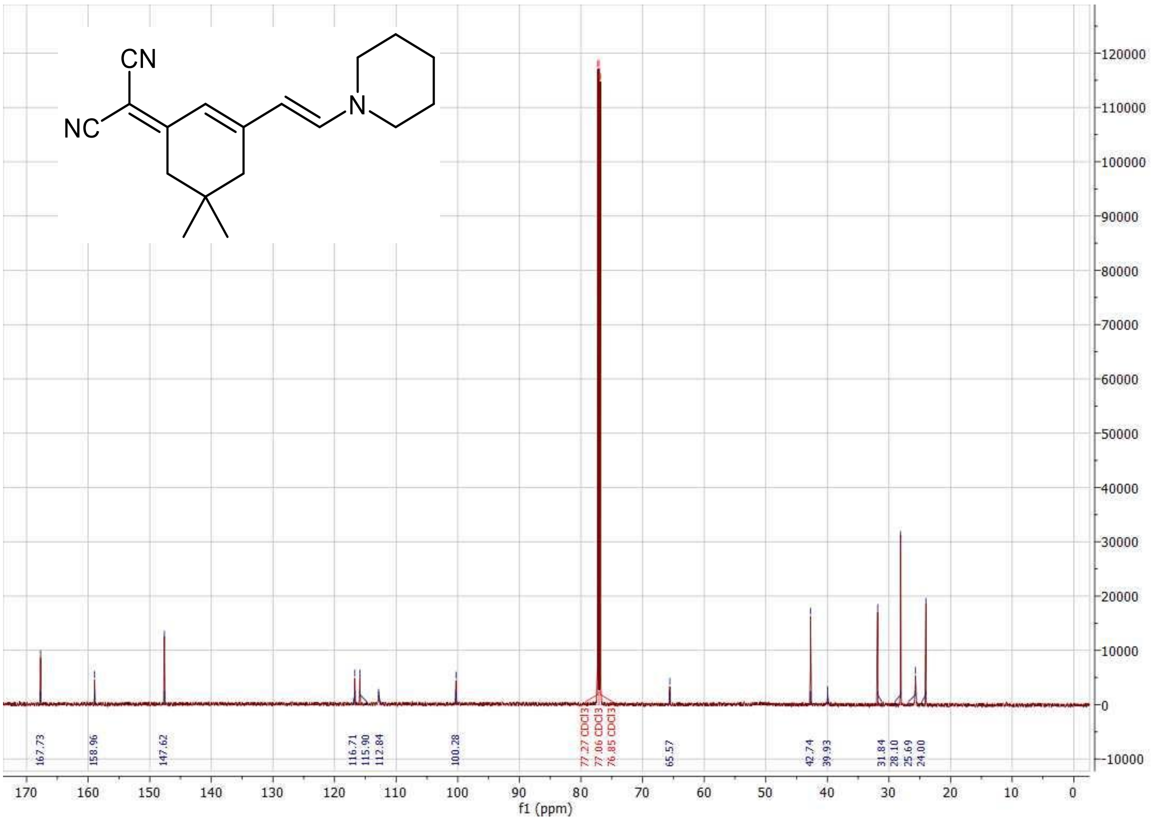

Figure S7. ^{13}C NMR spectrum of compound **6c** ($CDCl_3$, 151 MHz, 293 K).

5.5. (*E*)-2-[5,5-Dimethyl-3-(2-morpholino-2-phenylvinyl)cyclohex-2-en-1-ylidene]malononitrile (6d)

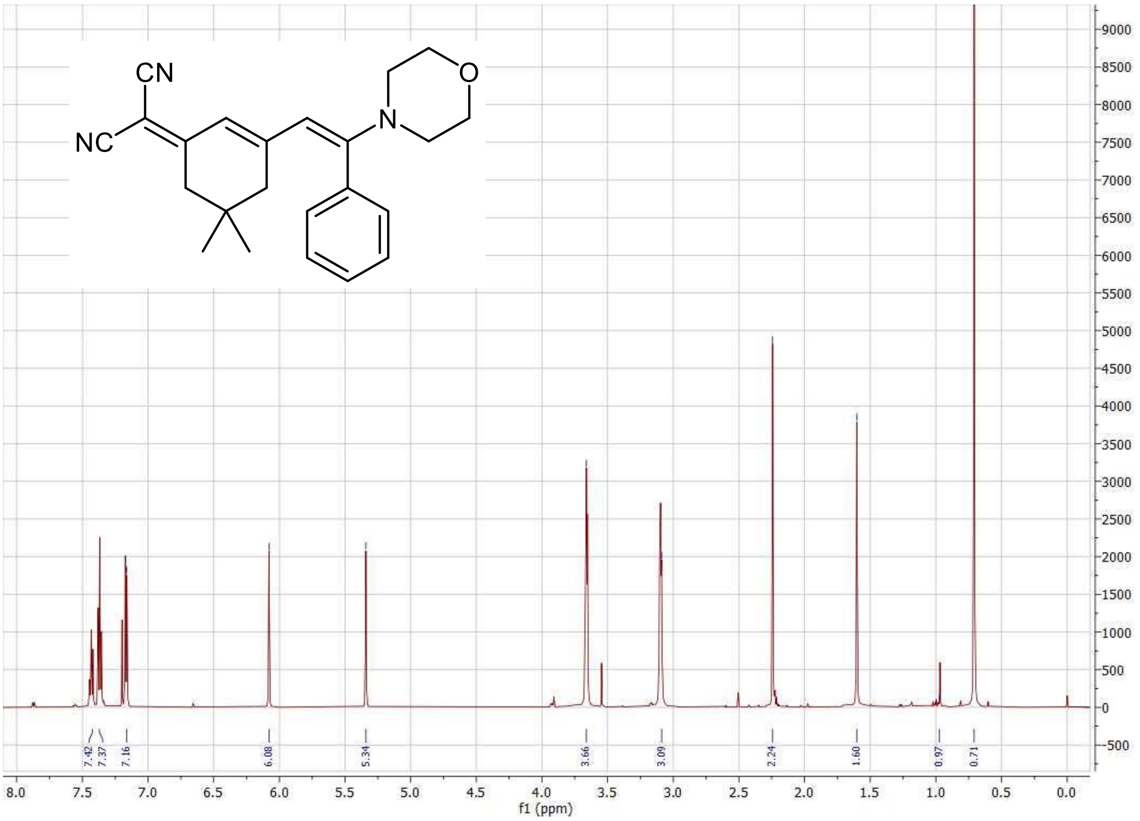

Figure S8. ^{1}H NMR spectrum of compound **6d** ($CDCl_3$, 600 MHz, 293 K).

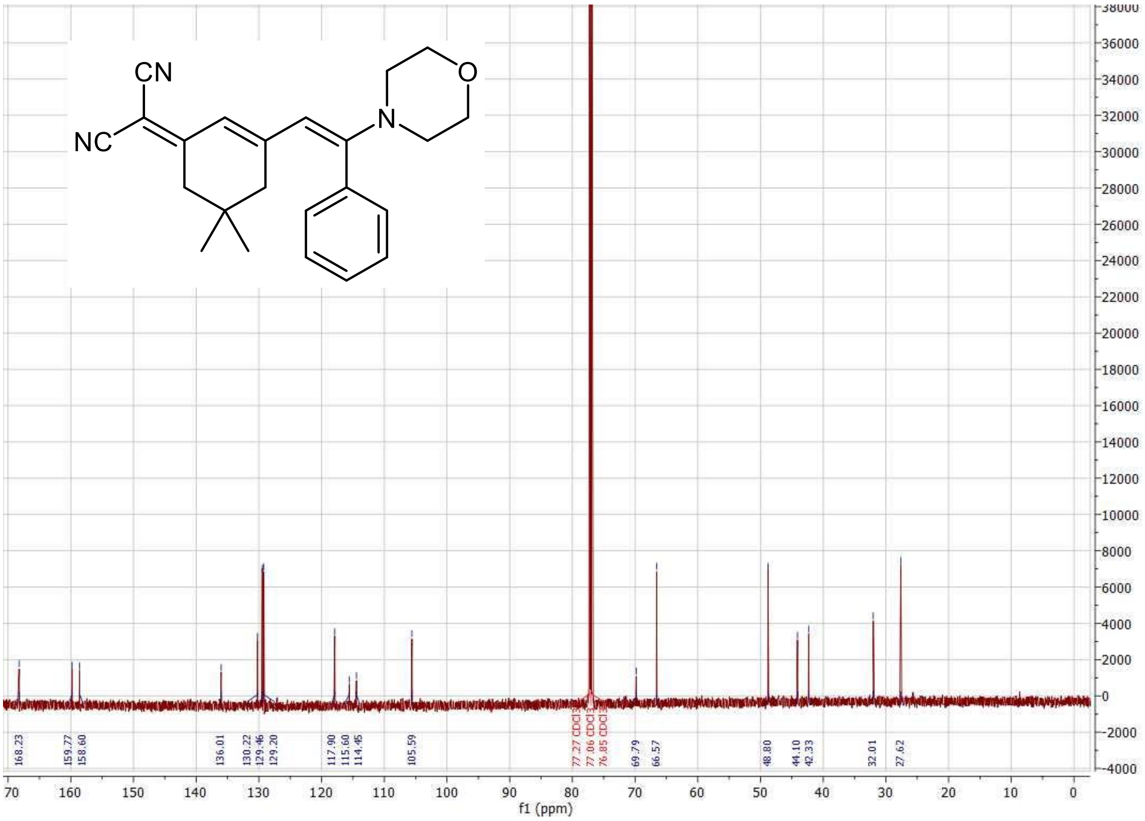

Figure S9. ^{13}C NMR spectrum of compound **6d** ($CDCl_3$, 151 MHz, 293 K).

5.6. (*E*)-2-{5,5-Dimethyl-3-[2-phenyl-2-(piperidin-1-yl)vinyl]cyclohex-2-en-1-ylidene}malononitrile (6e)

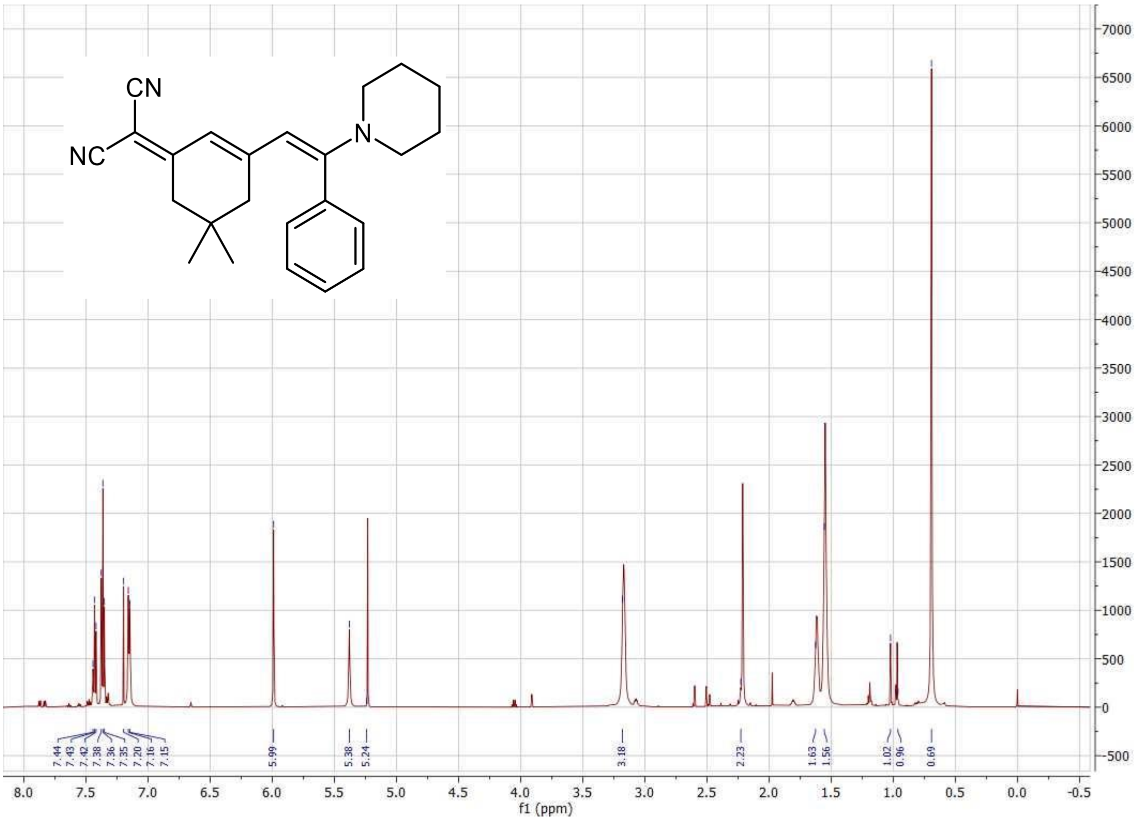

Figure S10. ^{1}H NMR spectrum of compound **6e** ($CDCl_3$, 600 MHz, 293 K).

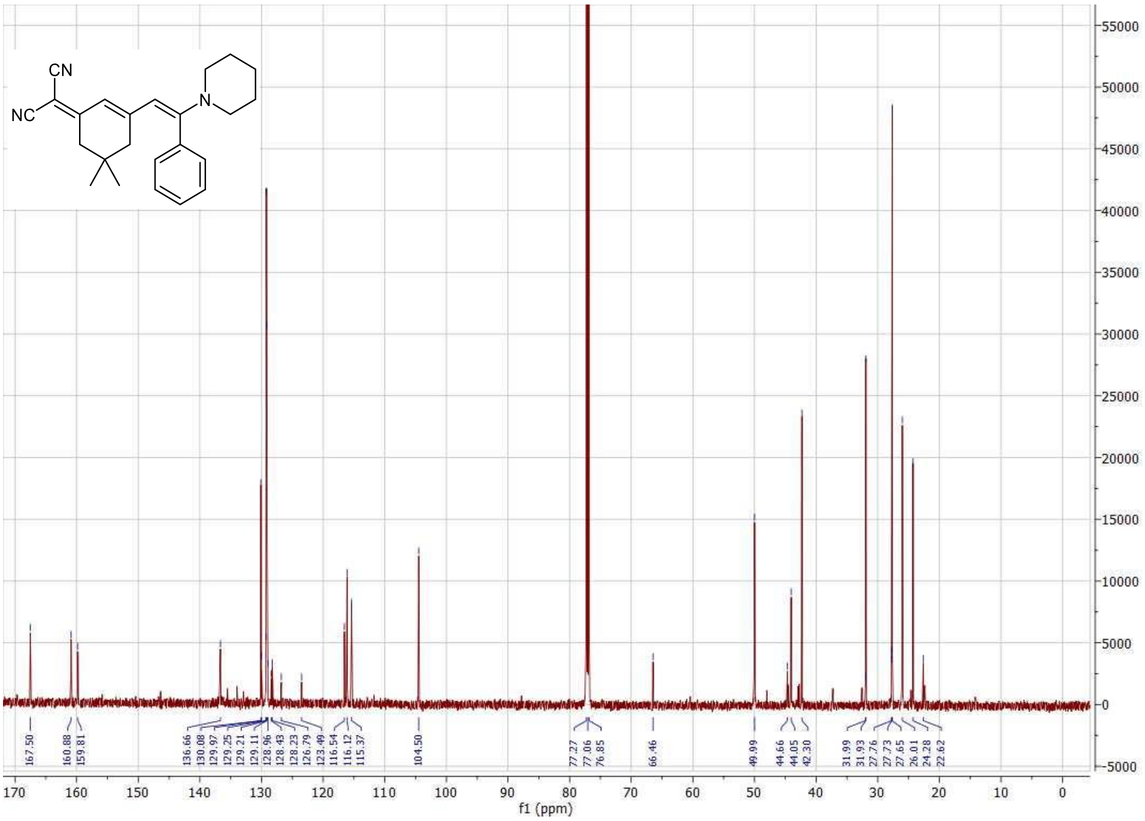

Figure S11. ^{13}C NMR spectrum of compound **6e** ($CDCl_3$, 151 MHz, 293 K).

5.7. Methyl-(*E*)-4-{2-[3-(dicyanomethylene)-5,5-dimethylcyclohex-1-en-1-yl]-1-(pyrrolidin-1-yl)vinyl}benzoate (6f)

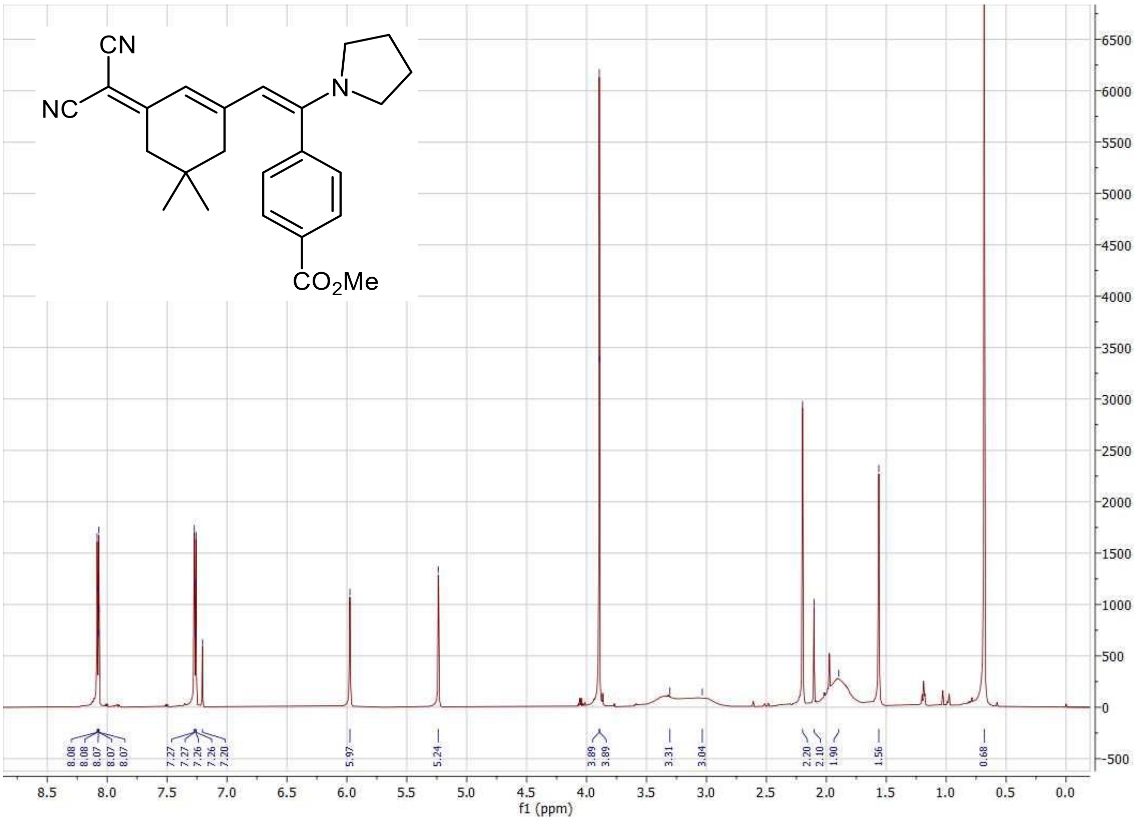

Figure S12. ^{1}H NMR spectrum of compound **6f** ($CDCl_3$, 600 MHz, 293 K).

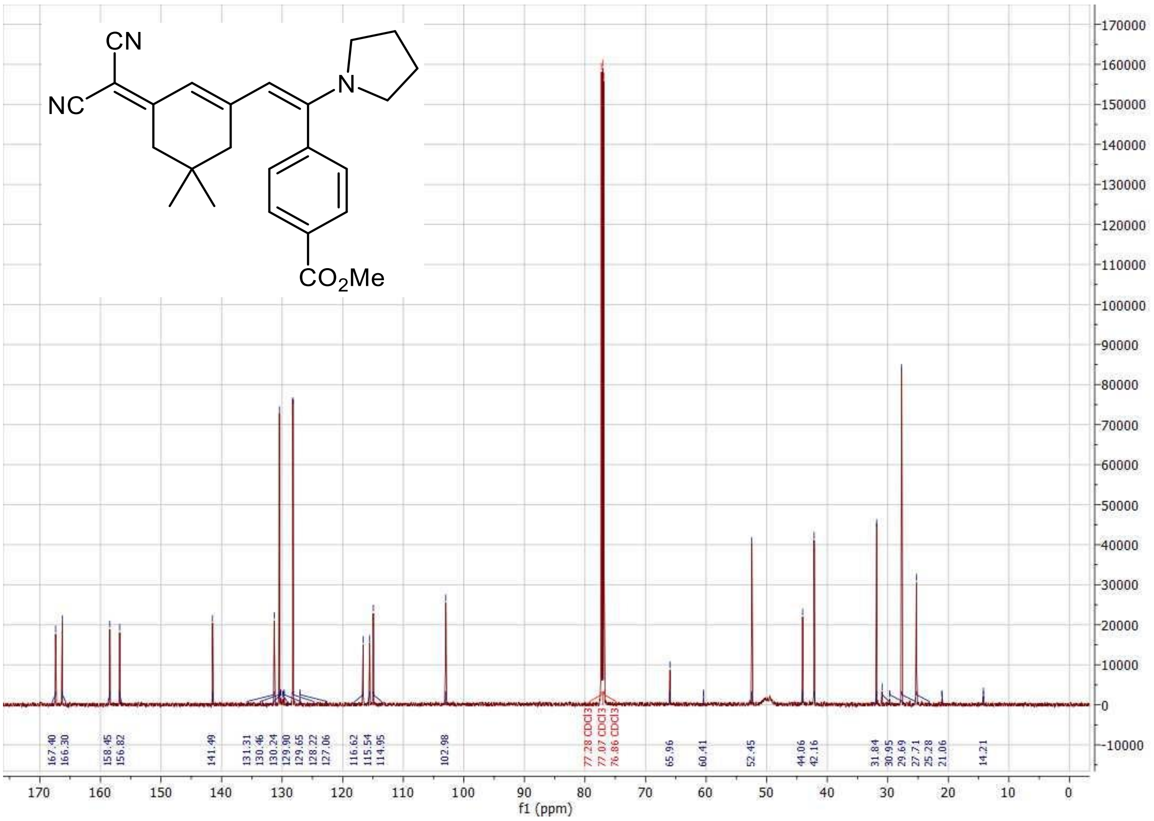

Figure S13. ^{13}C NMR spectrum of compound **6f** ($CDCl_3$, 151 MHz, 293 K).

5.8. (*E*)-2-{3-[2-(4-Methoxyphenyl)-2-(pyrrolidin-1-yl)vinyl]-5,5-dimethylcyclohex-2-en-1-ylidene}malononitrile (6g)

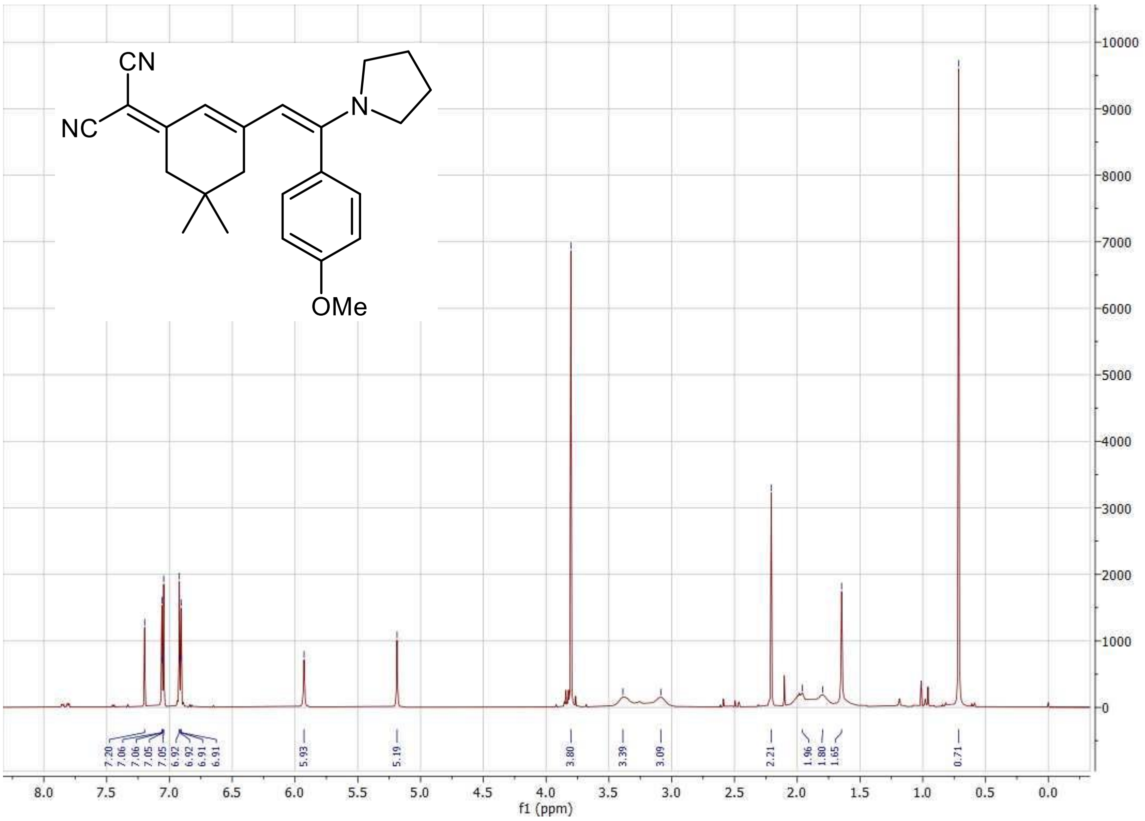

Figure S14. ^{1}H NMR spectrum of compound **6g** ($CDCl_3$, 600 MHz, 293 K).

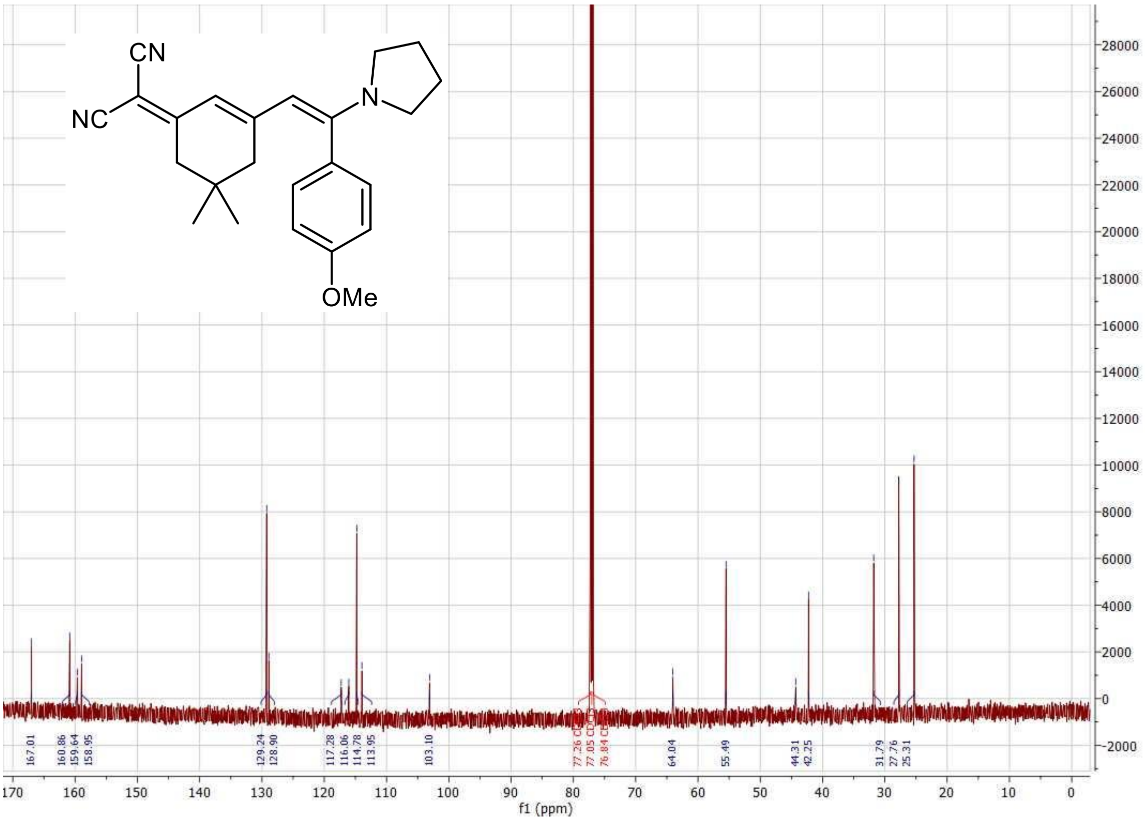

Figure S15. ^{13}C NMR spectrum of compound **6g** ($CDCl_3$, 151 MHz, 293 K).

5.9. (*E*)-2-(3-{2-[(4-Methoxyphenyl)amino]-2-phenylvinyl}-5,5-dimethylcyclohex-2-en-1-ylidene)malononitrile (6h)

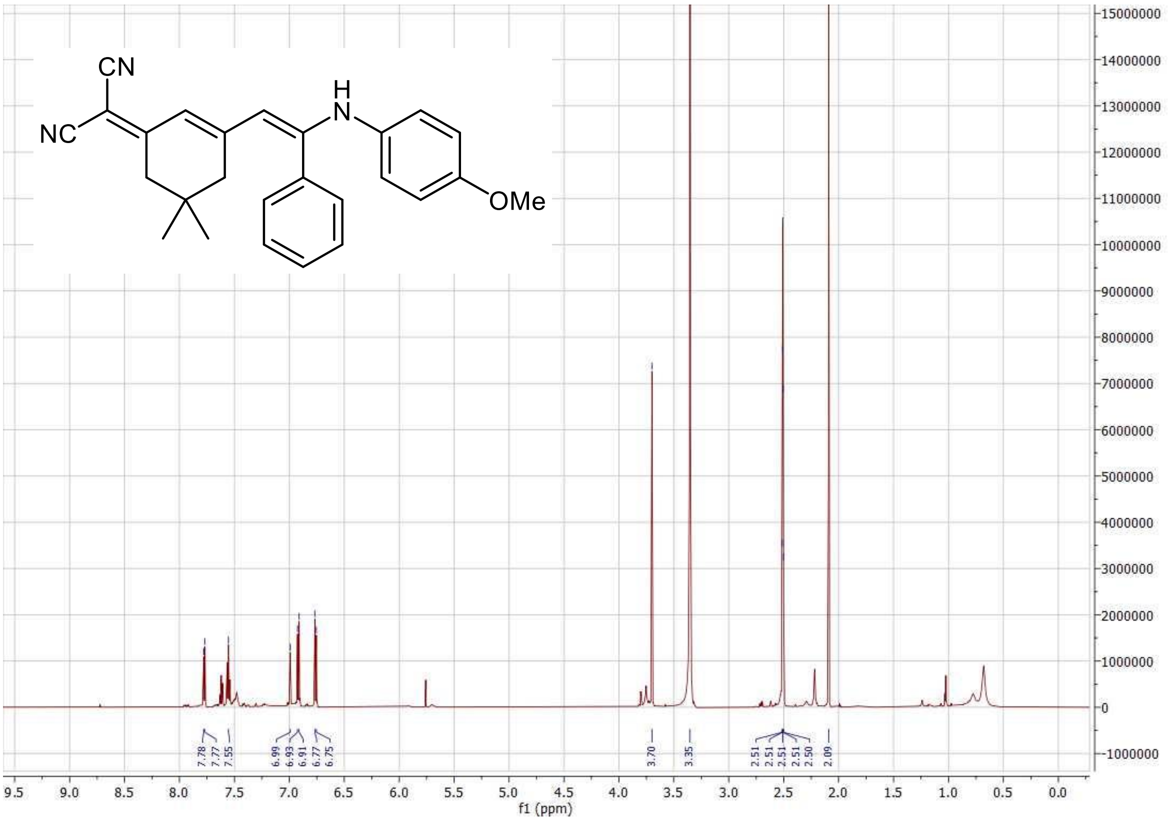

Figure S16. ^{1}H NMR spectrum of compound **6h** ($CDCl_3$, 600 MHz, 293 K).

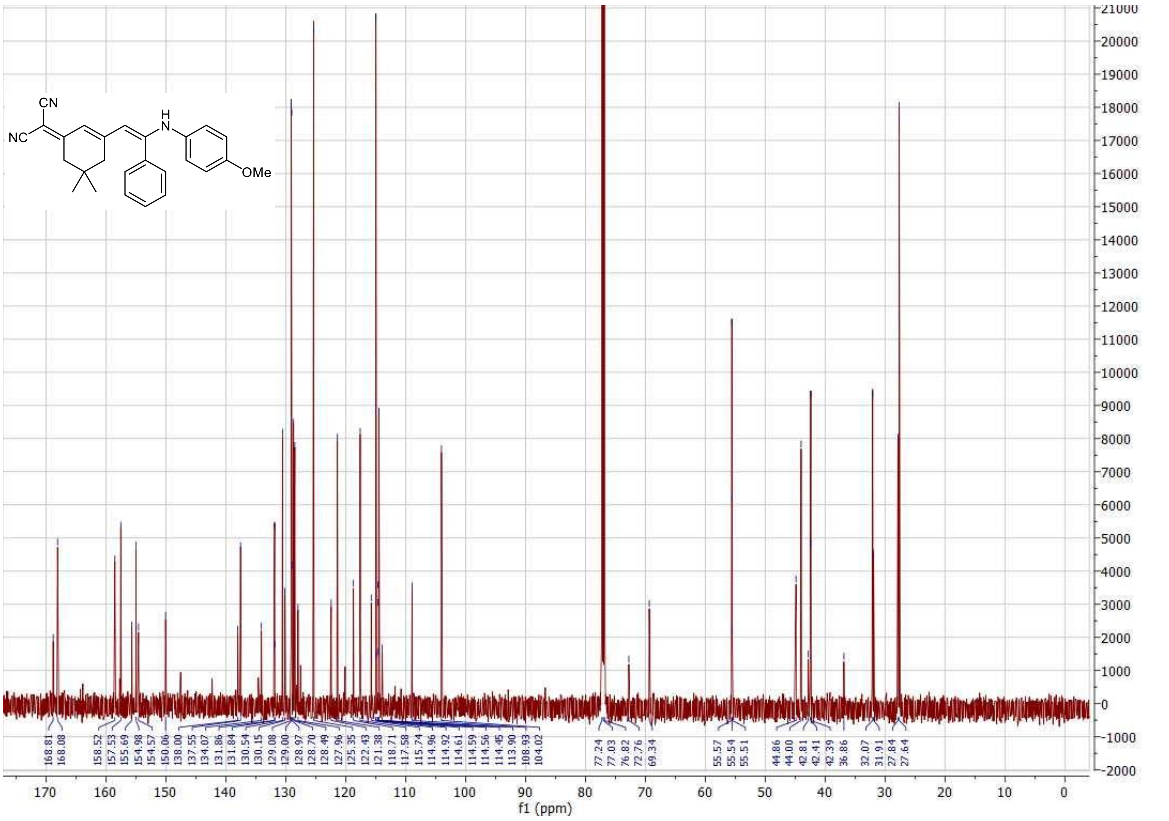

Figure S17. ^{13}C NMR spectrum of compound **6h** ($CDCl_3$, 151 MHz, 293 K).

5.10. 2,2'-{[(1*E*,1'*E*)-Piperazin-1,4-diylbis(2-phenylethen-2,1-diyl)]bis(5,5-dimethylcyclohex-2-en-3-yl-1-ylidene)}dimalononitrile (6i)

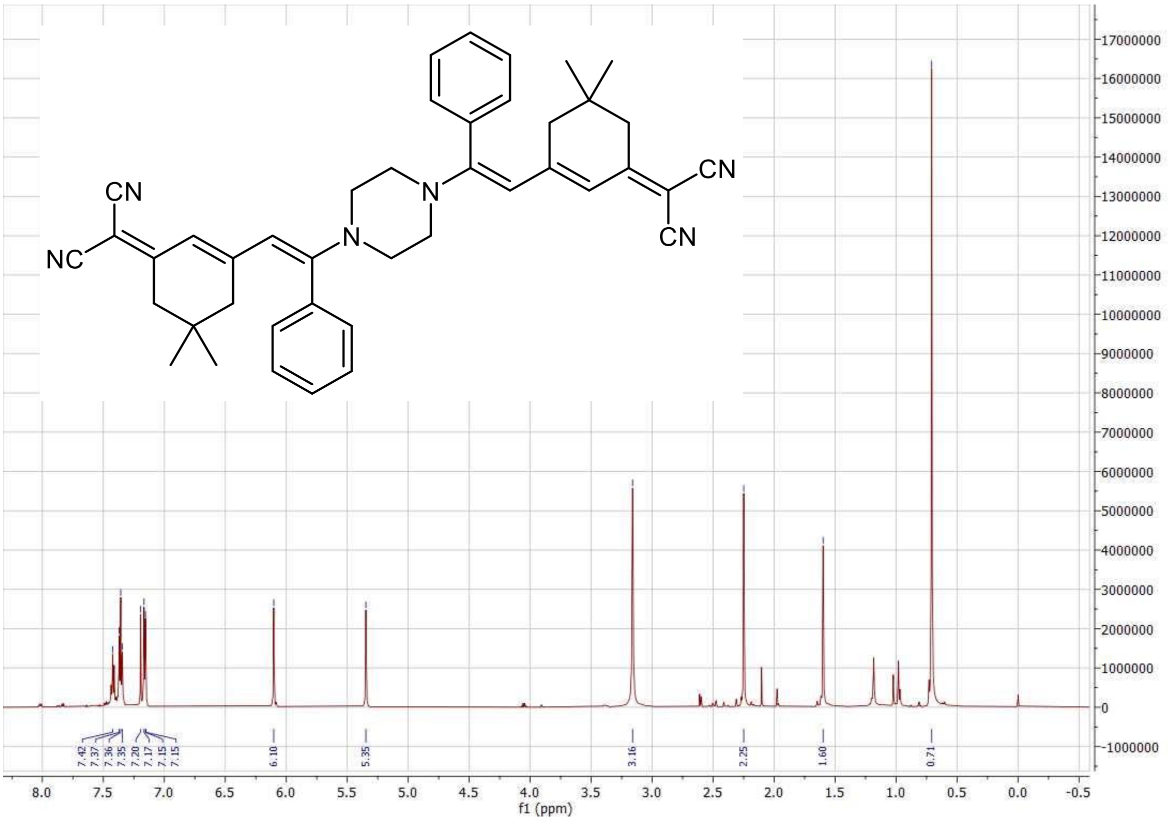

Figure S18. ^{1}H NMR spectrum of compound **6i** ($CDCl_3$, 600 MHz, 293 K).

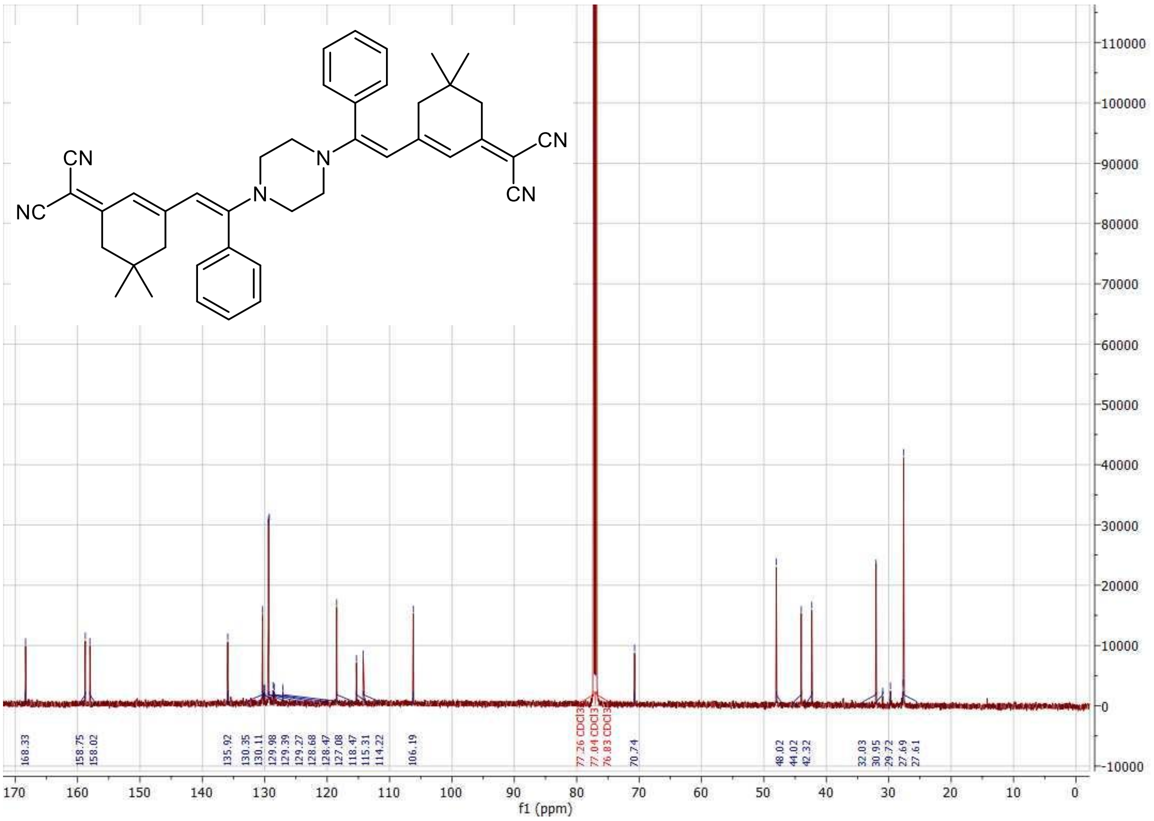

Figure S19. ^{13}C NMR spectrum of compound **6i** ($CDCl_3$, 151 MHz, 293 K).

5.11. 2-{5,5-Dimethyl-3-[3-(1,3,3-trimethylindolin-2-ylidene)-2-(trimethylsilyl)prop-1-en-1-yl]cyclohex-2-en-1-ylidene}malononitrile (8a)

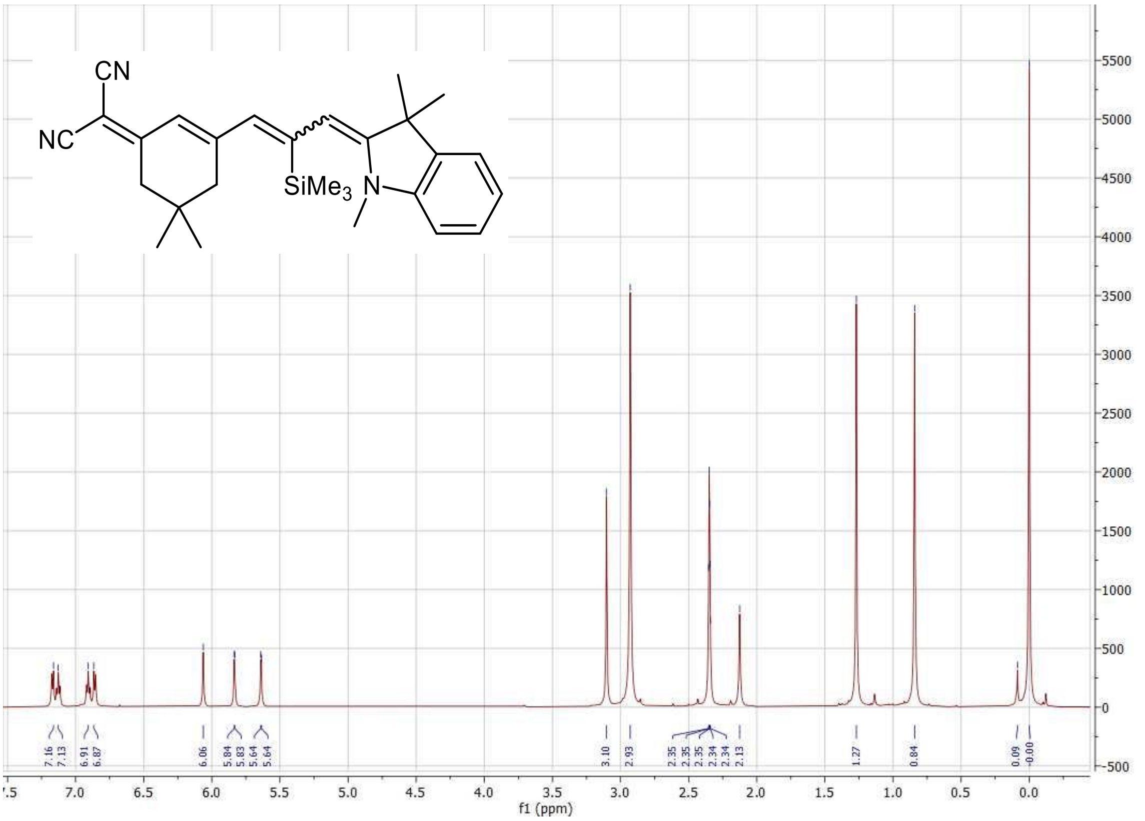

Figure S20. ^{1}H NMR spectrum of compound **8a** ($CDCl_3$, 600 MHz, 293 K).

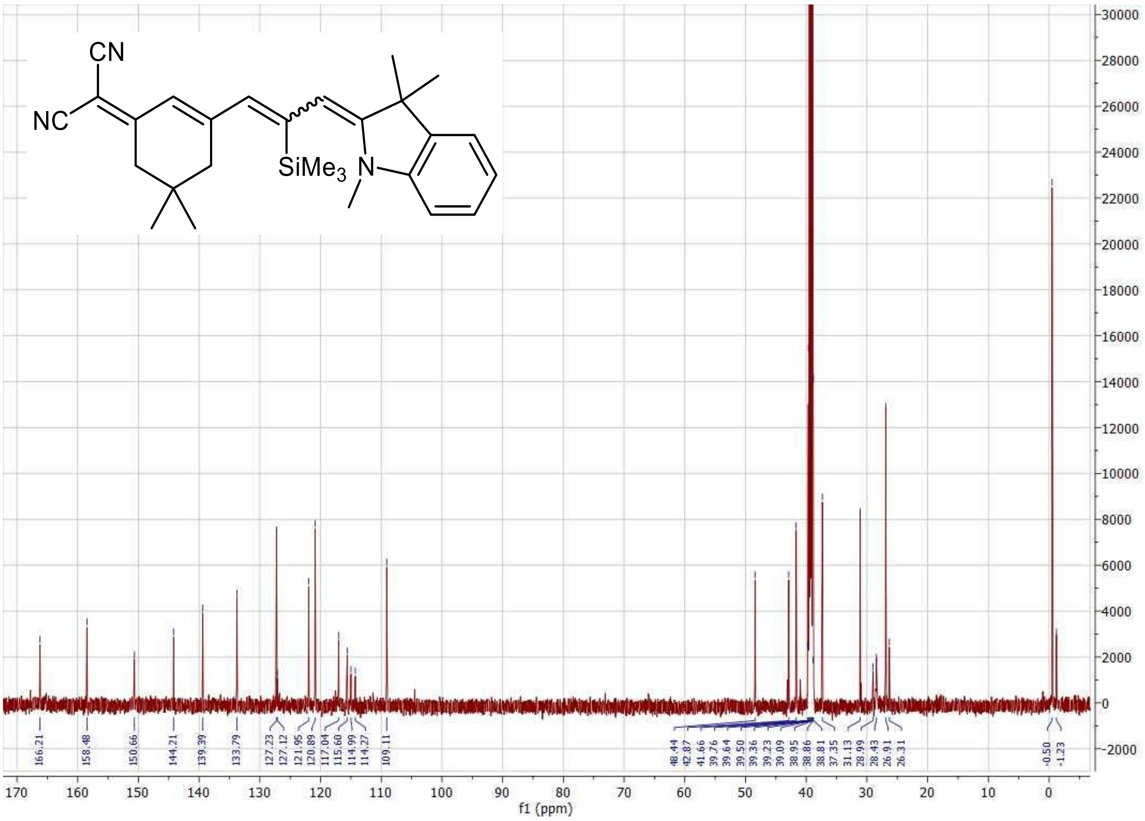

Figure S21. ^{13}C NMR spectrum of compound **8a** ($CDCl_3$, 151 MHz, 293 K).

5.12. 2-{5,5-Dimethyl-3-[2-phenyl-3-(1,3,3-trimethylindolin-2-ylidene)prop-1-en-1-yl]cyclohex-2-en-1-ylidene}malononitrile (8b)

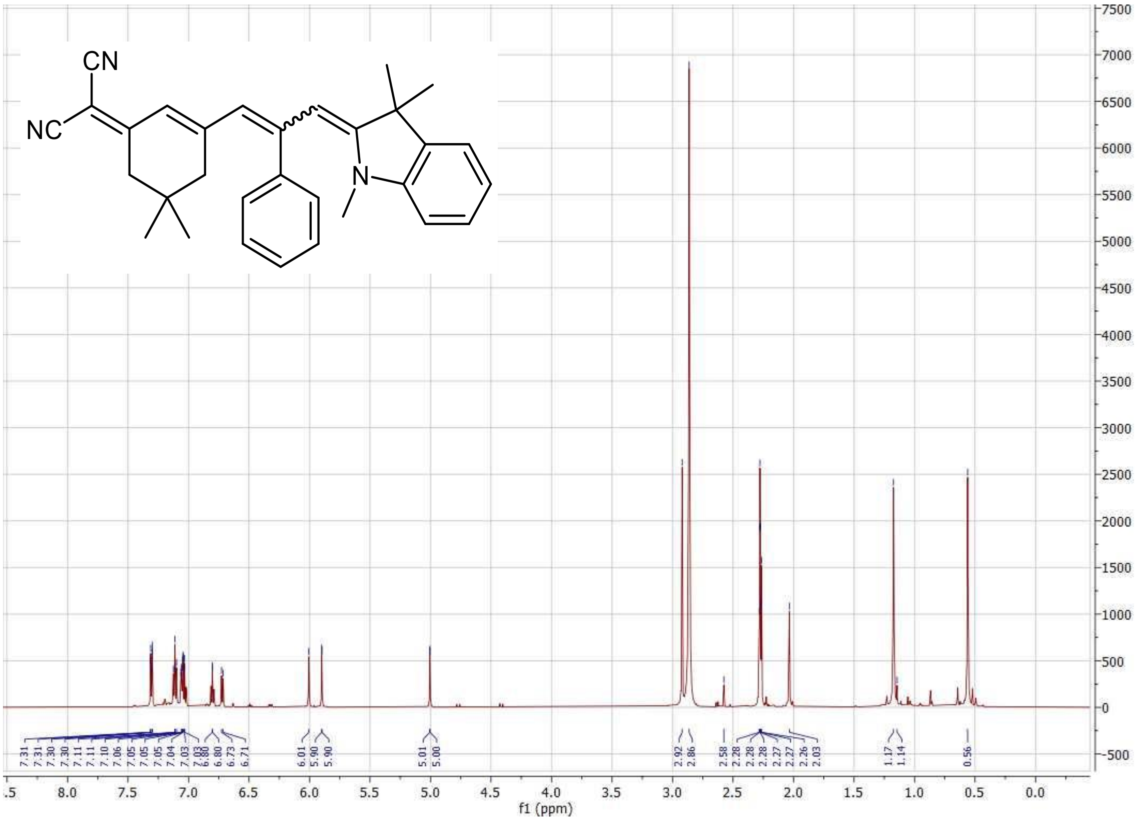

Figure S22. ^{1}H NMR spectrum of compound **8b** ($CDCl_3$, 600 MHz, 293 K).

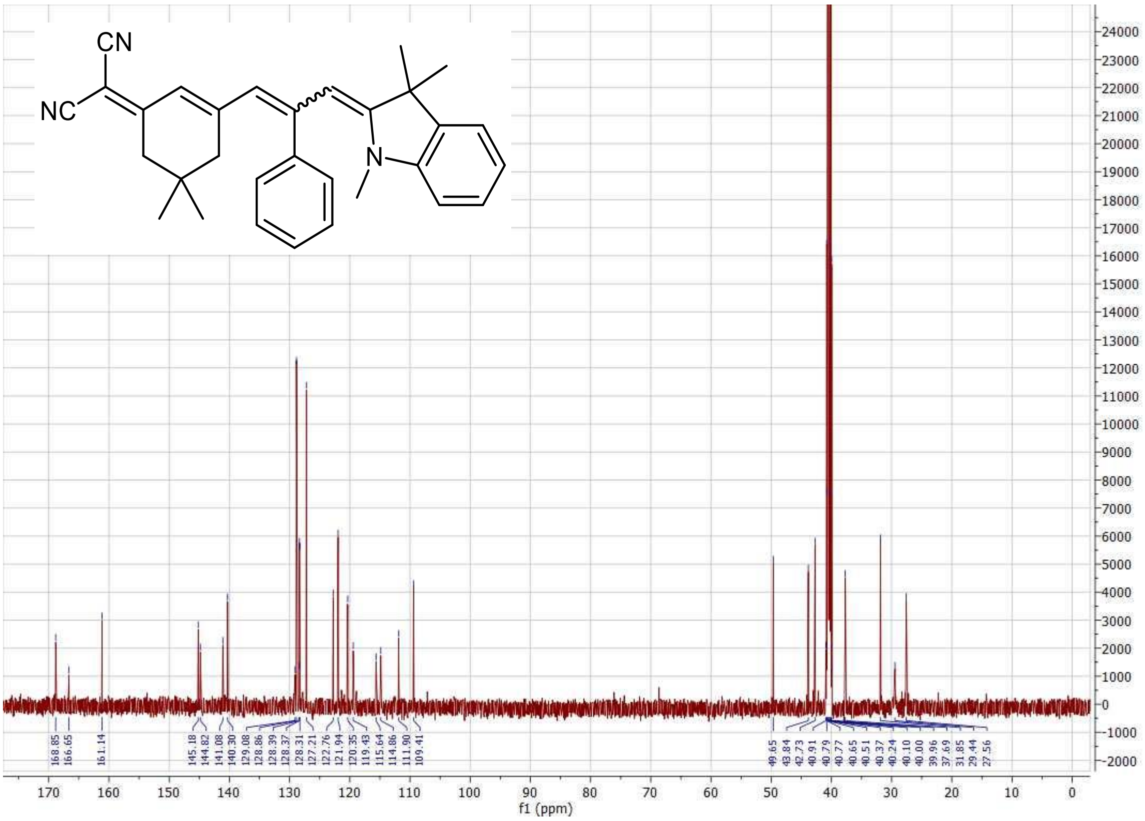

Figure S23. ^{13}C NMR spectrum of compound **8b** ($CDCl_3$, 151 MHz, 293 K).

5.13. 2-{3-[2-(4-Methoxyphenyl)-3-(1,3,3-trimethylindolin-2-ylidene)prop-1-en-1-yl]-5,5-dimethylcyclohex-2-en-1-ylidene}malononitrile (8c)

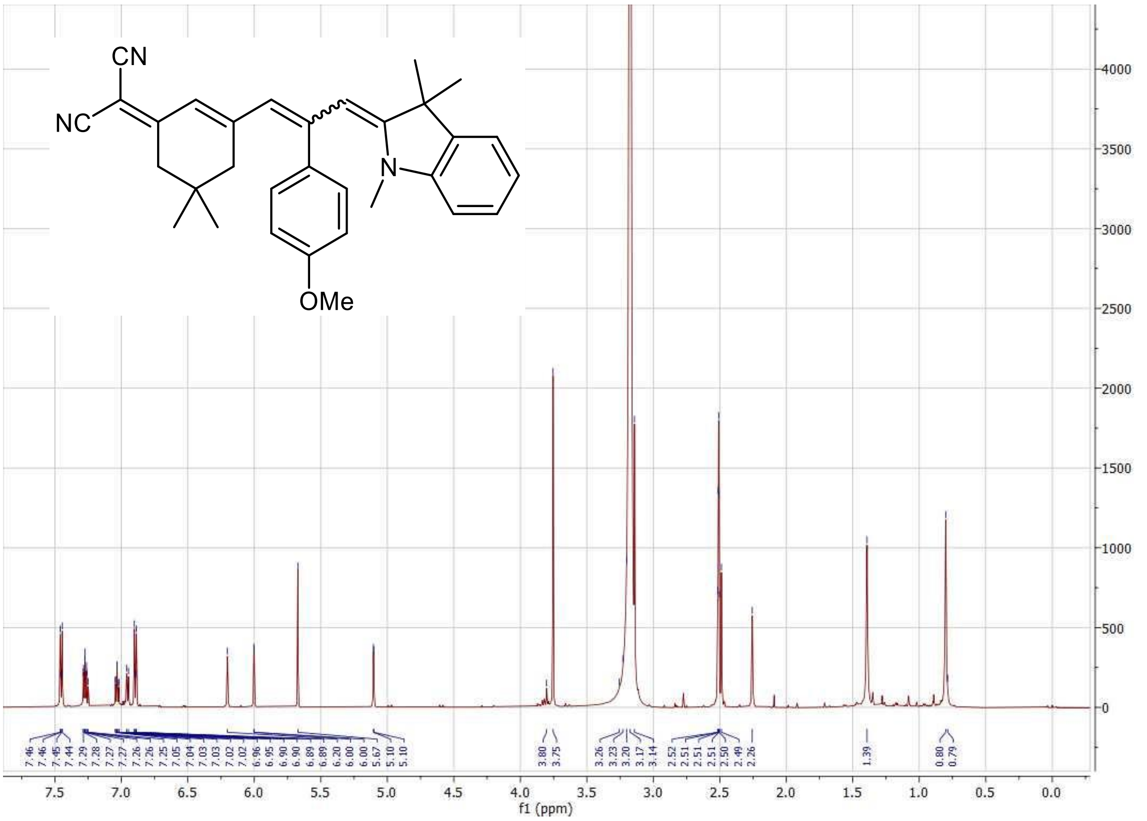

Figure S24. ^{1}H NMR spectrum of compound **8c** ($CDCl_3$, 600 MHz, 293 K).

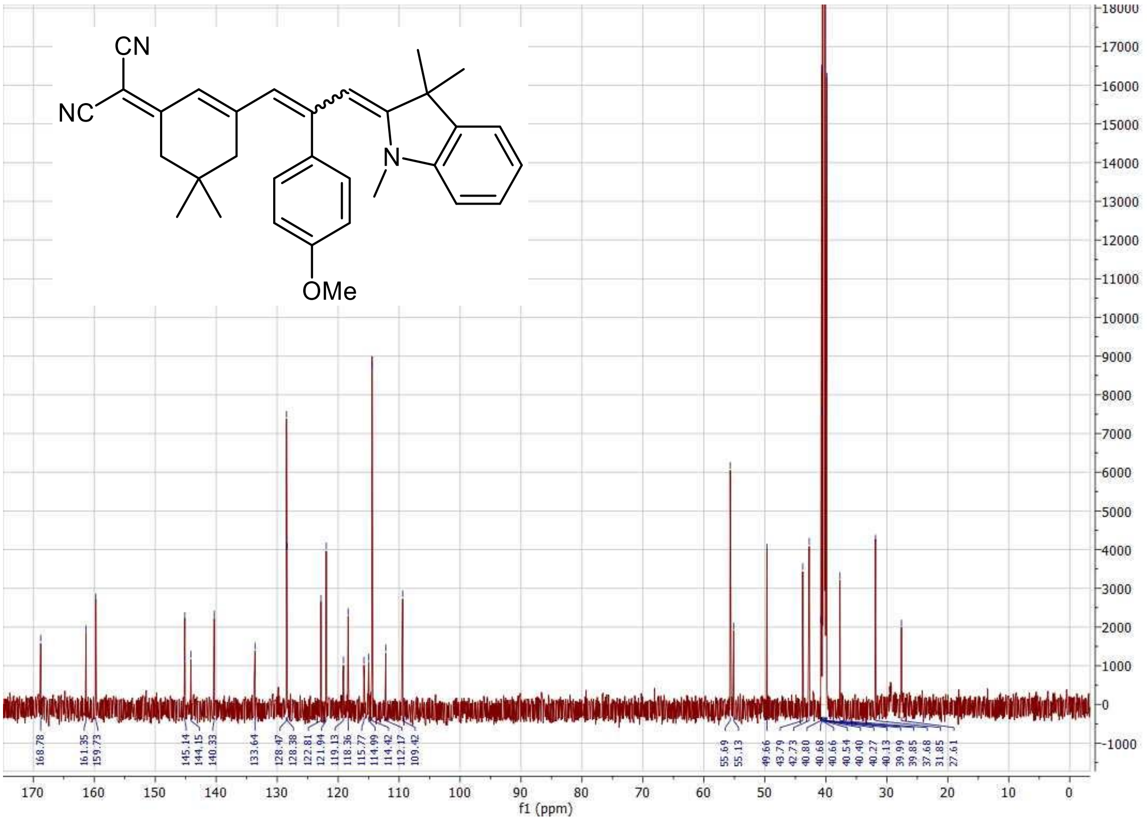

Figure S25. ^{13}C NMR spectrum of compound **8c** ($CDCl_3$, 151 MHz, 293 K).

6. UV/Vis and Emission Spectra of Merocyanines 4 and 6

6.1. (*E*)-2-{5,5-Dimethyl-3-[2-(pyrrolidin-1-yl)vinyl]cyclohex-2-en-1-ylidene}malononitrile (6a)

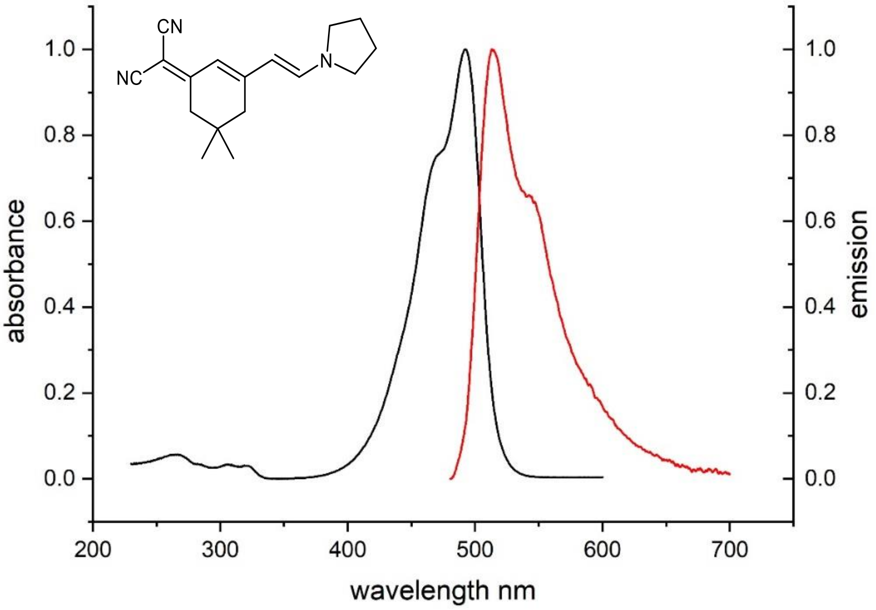

Figure S26. Normalized UV/Vis (black) and fluorescence (red) spectra of compound **6a** (recorded in CH_2Cl_2 at T = 293 K).

6.2. (*E*)-2-{5,5-Dimethyl-3-[2-phenyl-2-(pyrrolidin-1-yl)vinyl]cyclohex-2-en-1-ylidene}malononitrile (6b)

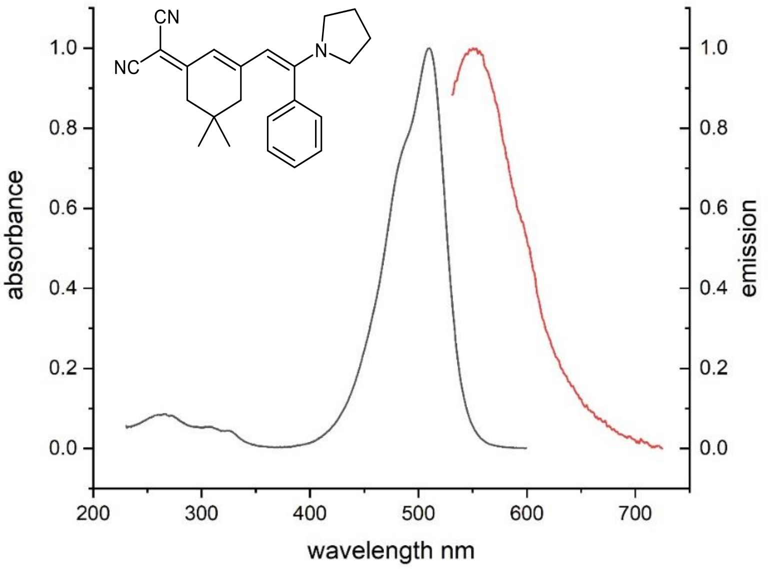

Figure S27. Normalized UV/Vis (black) and fluorescence (red) spectra of compound **6b** (recorded in CH_2Cl_2 at T = 293 K).

6.3. (*E*)-2-{5,5-Dimethyl-3-[2-(piperidin-1-yl)vinyl]cyclohex-2-en-1-ylidene}malononitrile (6c)

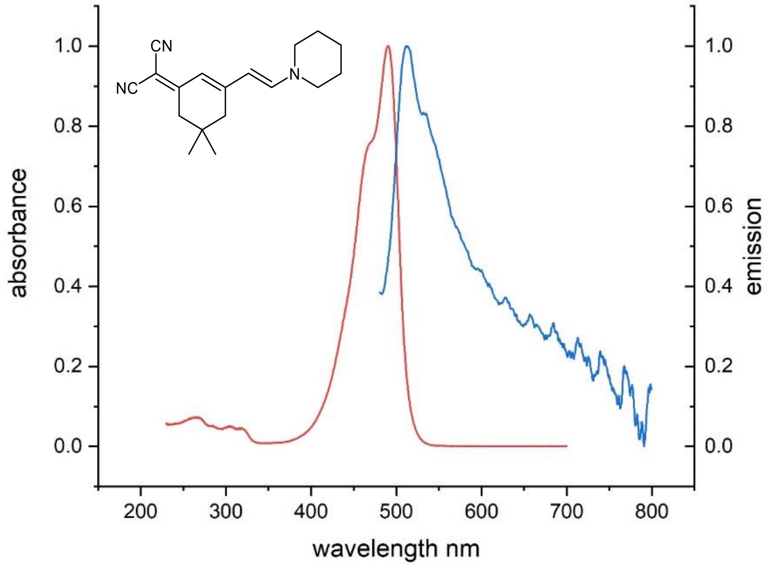

Figure S28. Normalized UV/Vis (black) and fluorescence (red) spectra of compound **6c** (recorded in CH_2Cl_2 at T = 293 K).

6.4. (*E*)-2-[5,5-Dimethyl-3-(2-morpholino-2-phenylvinyl)cyclohex-2-en-1-ylidene]malononitrile (6d)

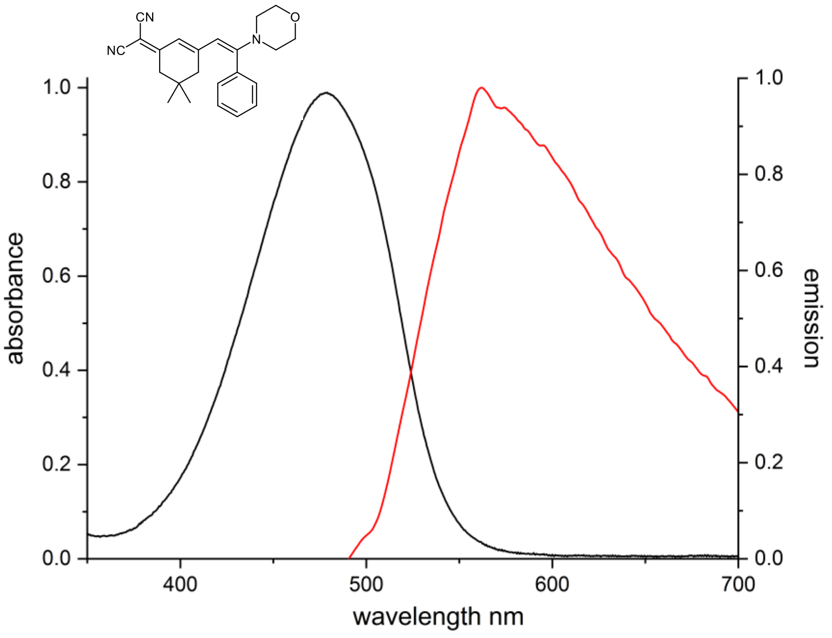

Figure S29. Normalized UV/Vis (black) and fluorescence (red) spectra of compound **6d** (recorded in CH_2Cl_2 at T = 293 K).

6.5. (*E*)-2-{5,5-Dimethyl-3-[2-phenyl-2-(piperidin-1-yl)vinyl]cyclohex-2-en-1-ylidene}malononitrile (6e)

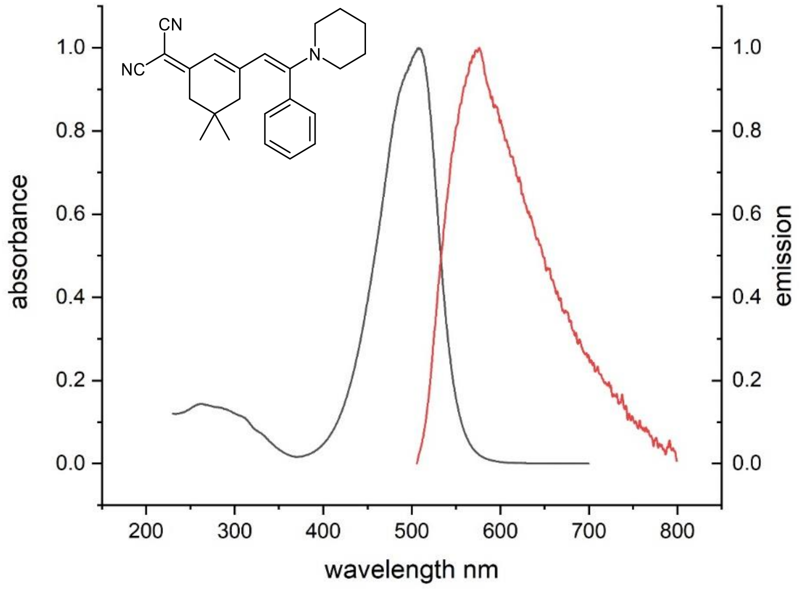

Figure S30. Normalized UV/Vis (black) and fluorescence (red) spectra of compound **6e** (recorded in CH_2Cl_2 at T = 293 K).

6.6. Methyl-(*E*)-4-{2-[3-(dicyanomethylene)-5,5-dimethylcyclohex-1-en-1-yl]-1-(pyrrolidin-1-yl)vinyl}benzoate (6f)

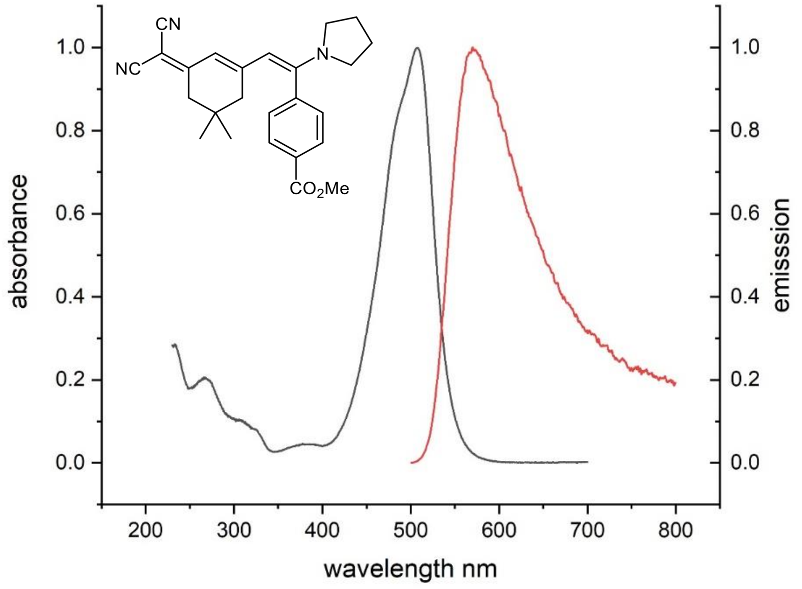

Figure S31. Normalized UV/Vis (black) and fluorescence (red) spectra of compound **6f** (recorded in CH_2Cl_2 at T = 293 K).

6.7. (*E*)-2-{3-[2-(4-Methoxyphenyl)-2-(pyrrolidin-1-yl)vinyl]-5,5-dimethylcyclohex-2-en-1-ylidene}malononitrile (6g)

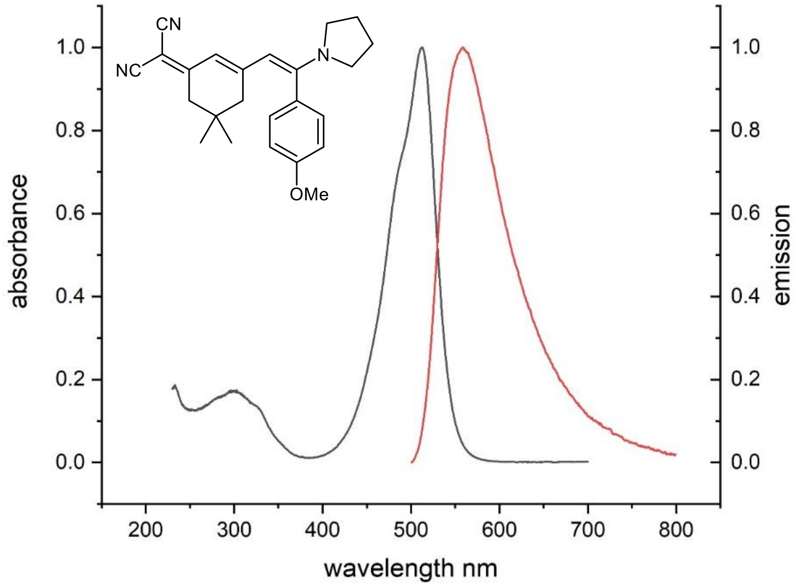

Figure S32. Normalized UV/Vis (black) and fluorescence (red) spectra of compound **6g** (recorded in CH_2Cl_2 at T = 293 K).

6.8. (*E*)-2-(3-{2-[(4-Methoxyphenyl)amino]-2-phenylvinyl}-5,5-dimethylcyclohex-2-en-1-ylidene)malononitrile (6h)

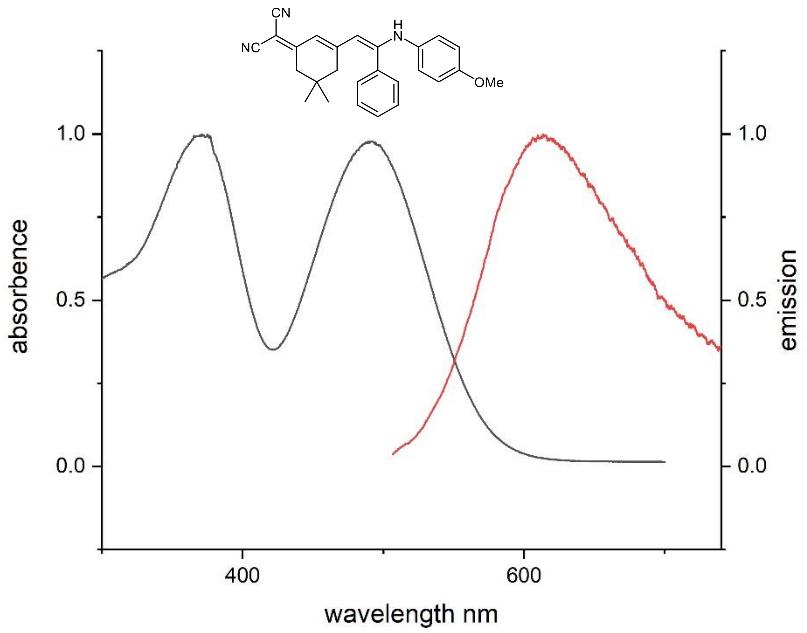

Figure S33. Normalized UV/Vis (black) and fluorescence (red) spectra of compound **6h** (recorded in CH_2Cl_2 at T = 293 K).

6.9. 2,2'-{[(1*E*,1'*E*)-Piperazin-1,4-diylbis(2-phenylethen-2,1-diyl)]bis(5,5-dimethylcyclohex-2-en-3-yl-1-ylidene)}dimalononitrile (6i)

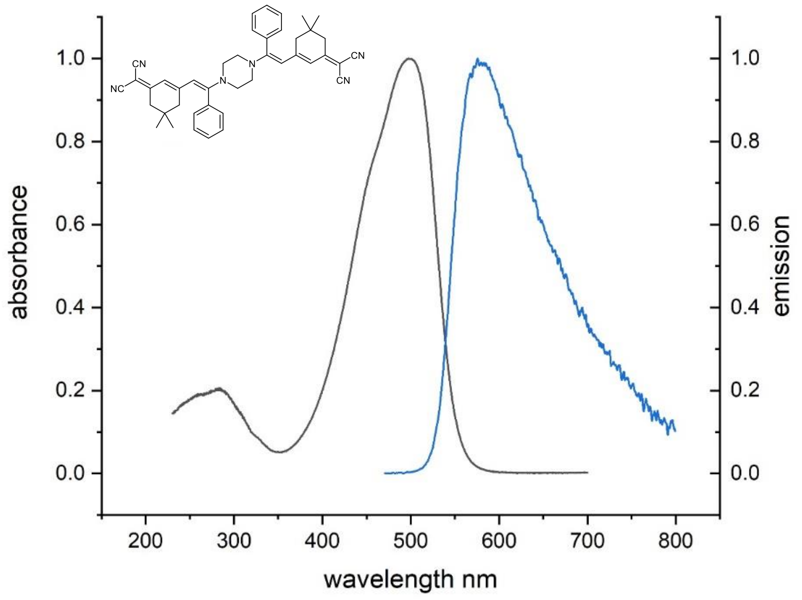

Figure S34. Normalized UV/Vis (black) and fluorescence (red) spectra of compound **6i** (recorded in CH_2Cl_2 at T = 293 K).

6.10. 2-{5,5-Dimethyl-3-[3-(1,3,3-trimethylindolin-2-ylidene)-2-(trimethylsilyl)prop-1-en-1-yl]cyclohex-2-en-1-ylidene}malononitrile (8a)

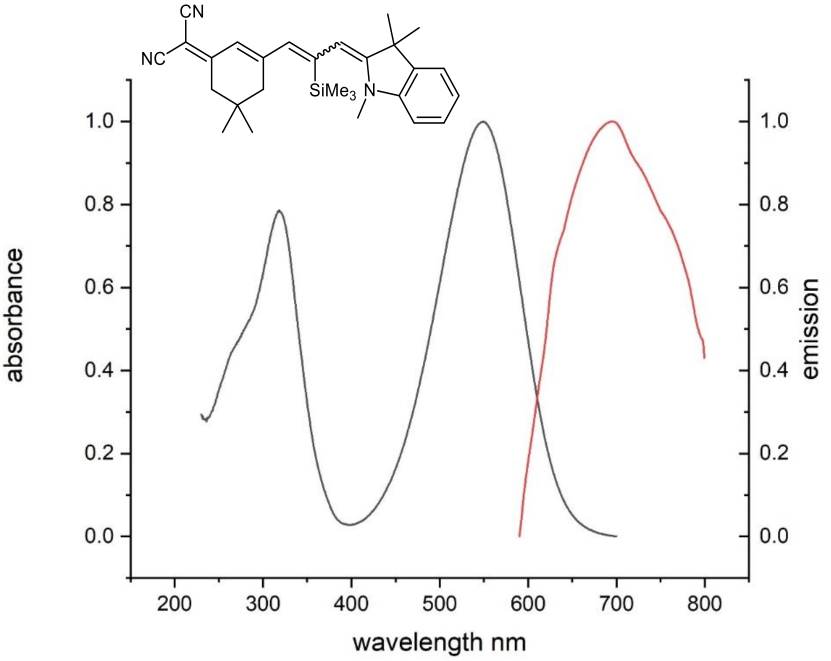

Figure S35. Normalized UV/Vis (black) and fluorescence (red) spectra of compound **8a** (recorded in CH_2Cl_2 at T = 293 K).

6.11. 2-{5,5-Dimethyl-3-[2-phenyl-3-(1,3,3-trimethylindolin-2-ylidene)prop-1-en-1-yl]cyclohex-2-en-1-ylidene}malononitrile (8b)

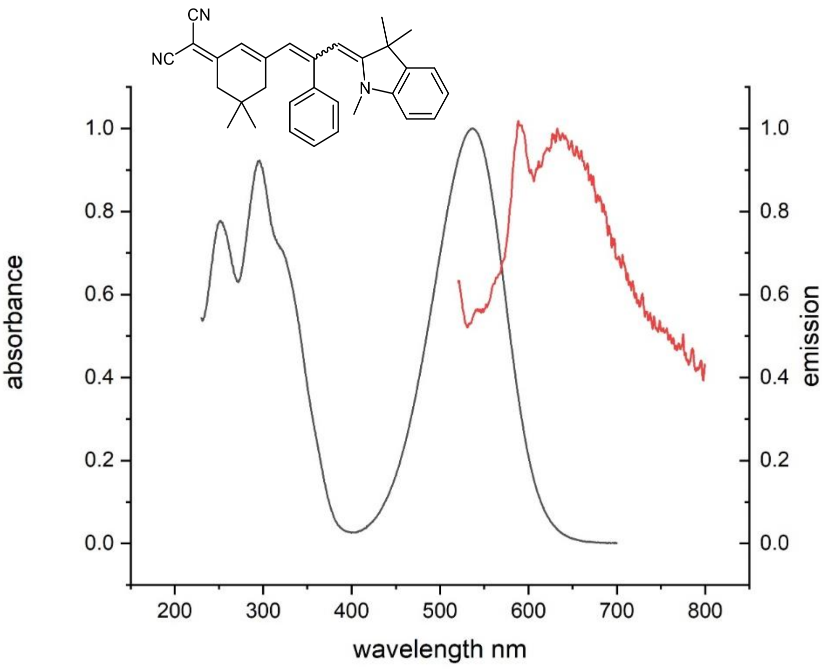

Figure S36. Normalized UV/Vis (black) and fluorescence (red) spectra of compound **8b** (recorded in CH_2Cl_2 at T = 293 K).

6.12. 2-{3-[2-(4-Methoxyphenyl)-3-(1,3,3-trimethylindolin-2-ylidene)prop-1-en-1-yl]-5,5-dimethylcyclohex-2-en-1-ylidene}malononitrile (8c)

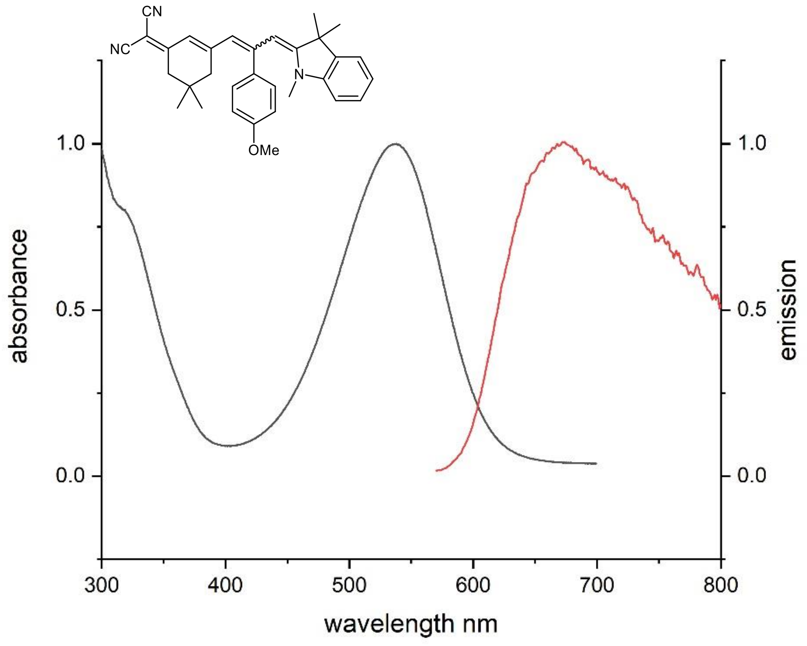

Figure S37. Normalized UV/Vis (black) and fluorescence (red) spectra of compound **8c** (recorded in CH_2Cl_2 at T = 293 K).

7. Crystal Structure of (*E*)-2-(3-{2-[(4-Methoxyphenyl)amino]-2-phenylvinyl}-5,5-dimethylcyclohex-2-en-1-ylidene)malononitrile (6h)

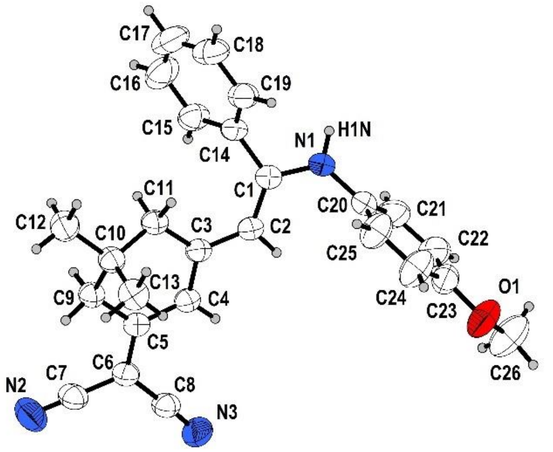

Figure S38. ORTEP plot (% probability) of merocyanine **6h**.

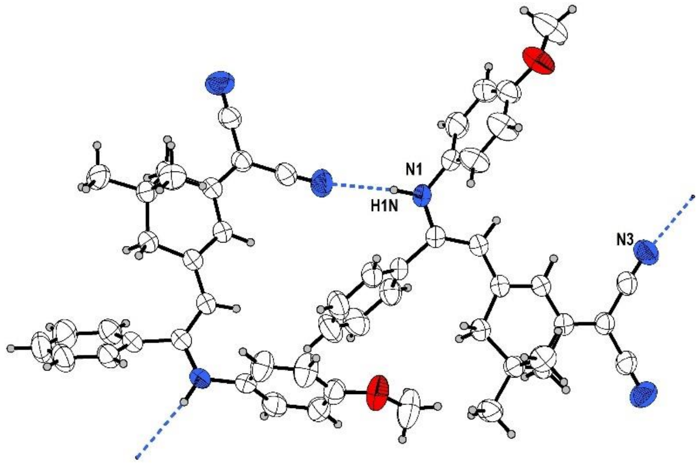

Figure S39. Intermolecular hydrogen bonding of merocyanine **6h** in the crystal.

Table S3. Crystal data and results of the structure refinement of merocyanine **6h**

Empirical formula, Formula weight	$C_{26}H_{25}N_3O$, 395.49
Diffractometer	Bruker, Three-circle Goniometer, APEX2 detector
X-ray source, Wavelength (Å)	Incoatec IμS, 1.54184
Temperature (K)	293
Crystal system	Monoclinic
Space group	$P2_1/c$

a (Å), *b* (Å), *c* (Å) , β (°)	12.3430(2), 12.0251(2), 15.3142(2),103.3560(10)
Volume (Å^3), Z	2211.55(6), 4
Z	4
δ_c(g cm^{-3})	1.188
Absorption coefficient (mm^{-1})	0.575
Crystal size (mm)	0.20 x 0.15 x 0.10
θ range for data collection (°)	4.16-67.48
Reflections collected	33628
Independent reflections	3915 [*R(int)* = 0.0255]
Reflections observed [I>2σI]	3732
Parameters / restraints	279 / 0
Goodness-of-fit	1.026
Final R indices [I > 2σ(I)]	*R1* = 0.0421, *wR2* = 0.0765
R indicies (all data)	*R1* = 0.0435, w*R2* = 0.0771
Largest diff. peak / hole, eA^{-3}	0.19 / -0.19

Table S4. Selected bond lengths of compound **6h**.

entry	bonding	bond length [Å]
1	N1-C1	1.3608
2	C1-C2	1.3734
3	C2-C3	1.4230
4	C3-C4	1.3810
5	C4-C5	1.4077
6	C5-C6	1.3834
7	C6-C8	1.4260
8	C8-N3	1.1432

8. Determining the Barriers from the VT ^{1}H NMR Spectra of Merocyanine 6f

Assuming a reversible dynamic process with first-order kinetics the rate constant *k* of the Eyring equation 1 is defined as:

$$k = \frac{RT}{N_A \cdot h} e^{\frac{-\Delta G^{\neq}}{RT}} \quad (1)$$

$\Delta G^{\neq}$ = Free activation enthalpy, N_A = Avogadro number, *h* = Planck constant.

For two separated signals A and B, the absorption of the exchanging nuclei is slow. In the case of fast conversions, these signals coincide. At the point where the signals merge is the coalescence temperature T_c. At this temperature, the rate constant k_{T_c} is approximated:

$$k_{T_c} = \frac{\pi}{\sqrt{2}} |\nu_A - \nu_B| \quad (2)$$

ν = absorption in Hz.

Substituting equation 2 into Eyring equation 1 gives:

$$\frac{\pi}{\sqrt{2}} |\nu_A - \nu_B| = \frac{RT_c}{N_A \cdot h} \cdot e^{-\frac{\Delta G^{\neq}}{RT_c}} \quad (3)$$

or transformed for the free activation enthalpy $\Delta G^{\neq}$:

$$\Delta G^{\neq} = RT_c \cdot ln \frac{RT_c\sqrt{2}}{\pi \cdot N_A \cdot h|\nu_A - \nu_B|} \quad (4)$$

R = gas constant, T_c = coalescence temperature in K.

For the free activation enthalpy $\Delta G^{\neq}$ results:

$$\Delta G^{\neq} = 19.1 \cdot 10^{-3} \cdot T_c(9.97 + \log T_c - \log|\nu_A - \nu_B|) \quad (5)$$

For protons $H^{9,9'}$, the coalescence temperature T_c is 318.15 K (45 °C). The difference in absorption $\Delta\nu$ is 279 Hz. For protons $H^{10,10'}$, the coalescence temperature T_c is 308.15 K (35 °C) and the difference in absorption $\Delta\nu$ is 96 Hz.

From this follows:

$H^{9,9'}$: $\Delta G^{\neq} = 19.1 \cdot 10^{-3} \cdot 318.15\, K \cdot (9.97 + \log 318.15 - \log|279|) = 61\, kJ/mol$ (6)

$H^{10,10'}$: $\Delta G^{\neq} = 19.1 \cdot 10^{-3} \cdot 308.15\, K \cdot (9.97 + \log 308.15 - \log|96|) = 61\, kJ/mol$ (7)

Thereby, $\Delta G_c^{\ddagger}$ for the rotation about the C-N bond lies at 61 kJ/mol.

At low temperatures, splitting into the triplet occurs. In the case of coupling nuclei, the following applies for the rate constant k_{Tc}:

$$k_{T_c} \approx 2.22\sqrt{\Delta\nu^2 + 6J_{AB}^2} \quad (8)$$

Applied to the free activation enthalpy:

$$\Delta G^{\neq} = RT_c \cdot ln \frac{RT_c\sqrt{2}}{\pi \cdot N_A \cdot h\sqrt{\Delta\nu^2 + 6J_{AB}^2}} = 19.1 \cdot 10^{-3} \cdot T_c\left(9.97 + \log T_c - \log\sqrt{\Delta\nu^2 + 6J_{AB}^2}\right) \quad (9)$$

For protons H[9,9'], the coalescence temperature T_c is 268.15 K (–5 °C). The difference in absorption $\Delta\nu$ is 253 Hz. For protons H[10,10'], the coalescence temperature T_c is 268.15 K (–5 °C) and the difference in absorption $\Delta\nu$ is 137 Hz.

From this follows:

H[9,9']: $\Delta G^{\neq} = 19.1 \cdot 10^{-3} \cdot 268.15\, K \cdot (9.97 + \log 268.15 - \log|254|) = 51\, kJ/mol$ (10)

H[10,10']: $\Delta G^{\neq} = 19.1 \cdot 10^{-3} \cdot 268.15\, K \cdot (9.97 + \log 268.15 - \log|137|) = 52\, kJ/mol$ (11)

Thereby, $\Delta G_c^{\ddagger}$ for the inversion of the pyrrolidine ring lies at approximately 51 kJ/mol.

9. Computational Data of Merocyanines 6a, 6b, 6f, and 6g

9.1. Computed xyz-coordinates (LC-ωB97XD/6-311++G** IEFPCM, dichloromethane) and excitation energies (LC-ωB97XD/6-311++G**) of merocyanine 6a

#p LC-ωB97XD 6-311++G** opt freq SCRF=(solvent=dichloromethane)

6a

C	2.390823	-0.375712	-0.173552
C	1.632884	2.026597	0.119312
C	-0.016390	0.101998	-0.169994
C	0.259160	1.568440	-0.392020
C	1.047631	-0.778935	-0.077155
C	2.698138	1.072922	-0.436520
C	3.442060	-1.283908	-0.066706
C	1.913490	3.443351	-0.387467
C	1.662634	2.026576	1.653914
C	3.209625	-2.668482	0.136777
N	3.005833	-3.794108	0.304260
C	4.797356	-0.880635	-0.169524
N	5.903906	-0.555237	-0.254216
C	-1.339723	-0.393827	-0.087407
C	-2.466320	0.391362	-0.196582
N	-3.718213	-0.042808	-0.140097
C	-4.092339	-1.443973	0.059932
C	-5.615771	-1.438765	-0.080281
C	-6.010955	-0.020847	0.344568
C	-4.891429	0.833429	-0.242603
H	0.195058	1.757145	-1.471661
H	-0.511995	2.183617	0.077826
H	0.831337	-1.830945	0.079494
H	2.761591	1.198116	-1.525785
H	3.681188	1.336127	-0.037311
H	2.890145	3.791940	-0.039061
H	1.156002	4.141664	-0.018986
H	1.909046	3.481512	-1.480936
H	2.643498	2.348358	2.015556
H	1.458041	1.036159	2.068227
H	0.913818	2.719672	2.048472
H	-1.457281	-1.463276	0.050799
H	-2.375508	1.462569	-0.345054
H	-3.772822	-1.767312	1.057105
H	-3.599485	-2.080880	-0.679015
H	-5.894466	-1.608686	-1.123692
H	-6.083731	-2.214101	0.525816
H	-6.010597	0.062834	1.434874
H	-6.992663	0.277897	-0.022642
H	-4.720352	1.763042	0.301974

Low frequencies --- -8.6574 -0.0004 0.0002 0.0006 0.9005 4.9914

Low frequencies --- 29.4266 38.4606 52.3194

Zero-point correction= 0.350024 (Hartree/Particle)
Sum of electronic and zero-point Energies= -824.273648a.u
Sum of electronic and thermal Energies= -824.254038a.u
Sum of electronic and thermal Enthalpies= -824.253094a.u
Sum of electronic and thermal Free Energies= -824.322582a.u

Excitation energy:

Excited State 1: Singlet-A 3.0303 eV 409.15 nm f=1.4112

(Corrected Linear Response: 384.35 nm)

HOMO -> LUMO 97.0%
Excited State 2: Singlet-A 5.0226 eV 246.85 nm f=0.0995
HOMO-1 -> LUMO 46.4%
HOMO-1 -> LUMO+8 5.5%
HOMO -> LUMO+3 4.9%
HOMO -> LUMO+8 26.1%
HOMO -> LUMO+11 2.1%
Excited State 3: Singlet-A 5.0426 eV 245.87 nm f=0.0725
HOMO-1 -> LUMO 34.7%
HOMO-1 -> LUMO+8 7.6%
HOMO -> LUMO+6 4.1%
HOMO -> LUMO+7 2.1%
HOMO -> LUMO+8 29.6%
HOMO -> LUMO+12 6.7%
Excited State 4: Singlet-A 5.1472 eV 240.88 nm f=0.0122
HOMO-1 -> LUMO 4.4%
HOMO -> LUMO+1 57.0%
HOMO -> LUMO+3 9.8%
HOMO -> LUMO+4 8.0%
HOMO -> LUMO+5 7.5%
HOMO -> LUMO+17 2.2%
Excited State 5: Singlet-A 5.5226 eV 224.50 nm f=0.0082
HOMO-1 -> LUMO 9.6%
HOMO -> LUMO+2 4.6%
HOMO -> LUMO+3 26.6%
HOMO -> LUMO+4 21.2%
HOMO -> LUMO+7 5.0%
HOMO -> LUMO+11 5.9%
HOMO -> LUMO+12 2.6%
HOMO -> LUMO+13 11.0%
Excited State 6: Singlet-A 5.6769 eV 218.40 nm f=0.0132
HOMO-1 -> LUMO+1 3.6%
HOMO -> LUMO+1 12.4%
HOMO -> LUMO+3 15.4%
HOMO -> LUMO+4 35.1%
HOMO -> LUMO+6 3.1%
HOMO -> LUMO+9 8.8%
HOMO -> LUMO+10 2.1%

HOMO -> LUMO+11 2.5%

9.2. Computed xyz-coordinates (LC-ωB97XD/6-311++G** IEFPCM, dichloromethane) S_1 and emission energies (LC-ωB97XD/6-311++G**) of merocyanine 6a

#p LC-ωB97XD 6-311++G** opt freq SCRF=(solvent=dichloromethane) TD=(singlet, n states=6,root=1)

6a

C	2.388801	-0.364728	-0.179833
C	1.676625	2.047948	0.127571
C	-0.026671	0.182974	-0.147817
C	0.279356	1.637022	-0.373672
C	1.045920	-0.734678	-0.060201
C	2.722866	1.076374	-0.446772
C	3.429153	-1.333458	-0.081444
C	1.989207	3.465211	-0.357102
C	1.714794	2.014956	1.661312
C	3.160409	-2.701582	0.128742
N	2.912367	-3.821600	0.303699
C	4.788190	-0.976720	-0.193287
N	5.904759	-0.674050	-0.287814
C	-1.338855	-0.315811	-0.076546
C	-2.499143	0.452892	-0.220614
N	-3.739232	-0.028225	-0.137116
C	-4.076313	-1.428338	0.108353
C	-5.595011	-1.475790	-0.066401
C	-6.042823	-0.058114	0.301122
C	-4.930360	0.810192	-0.278483
H	0.203738	1.856613	-1.448786
H	-0.466765	2.268636	0.120848
H	0.803178	-1.780121	0.109920
H	2.795238	1.226723	-1.533581
H	3.708280	1.323747	-0.039483
H	2.976755	3.784702	-0.010888
H	1.251494	4.177209	0.025684
H	1.978915	3.519315	-1.450048
H	2.705635	2.301746	2.025849
H	1.487350	1.019584	2.052548
H	0.984814	2.716196	2.076815
H	-1.452546	-1.382269	0.093047
H	-2.434044	1.519124	-0.408880
H	-3.769416	-1.704793	1.125477
H	-3.546019	-2.079272	-0.593143
H	-5.842647	-1.690799	-1.109209
H	-6.051634	-2.244475	0.556556
H	-6.080586	0.060792	1.387219
H	-7.019658	0.200750	-0.107012
H	-4.789951	1.757444	0.246842

Low frequencies --- -7.4709 -0.0009 -0.0003 0.0001 0.8893 8.2776
Low frequencies --- 24.5590 32.6583 47.2118
Zero-point correction 0.348121 (Hartree/Particle)
Sum of electronic and zero-point Energies= -824.174985a.u
Sum of electronic and thermal Energies= -824.155083a.u
Sum of electronic and thermal Enthalpies= -824.154139a.u
Sum of electronic and thermal Free Energies= -824.224671a.u

Emission energy

Excited State 1: Singlet-A 3.0804 eV 402.49 nm f=1.2400

corrected external emission excitation: 3.0991 eV 400.07 nm
HOMO -> LUMO 97.2%

9.3. Computed xyz-coordinates (LC-ωHPBE/6-311++G** IEFPCM, dichloromethane) and excitation energies (LC-ωHPBE/6-311++G**) of merocyanine 6a

#p LC-ωHPBE 6-311++G** opt freq SCRF=(solvent=dichloromethane)
6a
C 2.387392 -0.373142 -0.169168
C 1.630832 2.016501 0.122604
C -0.013686 0.097494 -0.148790
C 0.256111 1.560340 -0.366720
C 1.042626 -0.778206 -0.056903
C 2.683386 1.068810 -0.448359
C 3.432012 -1.272741 -0.070281
C 1.901792 3.428755 -0.385128
C 1.684301 2.014853 1.650045
C 3.203083 -2.658161 0.145733
N 3.011281 -3.779427 0.322161
C 4.787776 -0.869398 -0.194160
N 5.889452 -0.549971 -0.295504
C -1.340751 -0.398959 -0.069968
C -2.459280 0.384592 -0.184555
N -3.712905 -0.043372 -0.137347
C -4.091430 -1.438732 0.057016
C -5.607431 -1.427196 -0.099770
C -5.999013 -0.017154 0.330228
C -4.879440 0.832839 -0.248187
H 0.172799 1.754524 -1.442975
H -0.508455 2.169240 0.119927
H 0.829945 -1.830582 0.101384
H 2.718409 1.183458 -1.538920
H 3.674618 1.336861 -0.075624
H 2.881408 3.778895 -0.049975
H 1.149312 4.125437 -0.006579

H 1.882644 3.467842 -1.477450
H 2.669942 2.334747 1.997350
H 1.484546 1.024845 2.065487
H 0.943918 2.708052 2.057375
H -1.457536 -1.468626 0.066274
H -2.364056 1.456032 -0.331532
H -3.785501 -1.765809 1.056568
H -3.593722 -2.077527 -0.676451
H -5.874413 -1.586105 -1.147387
H -6.086720 -2.206485 0.491880
H -5.999133 0.060199 1.420415
H -6.980006 0.286335 -0.034333
H -4.712533 1.762536 0.297058
H -5.069939 1.072551 -1.299473
Low frequencies --- -6.2780 -0.0007 -0.0004 -0.0003 1.0770 4.1486
Low frequencies --- 30.4887 38.2623 53.5943
Zero-point correction 0.353361 (Hartree/Particle)
Sum of electronic and zero-point Energies= -823.957314a.u
Sum of electronic and thermal Energies= -823.937778a.u
Sum of electronic and thermal Enthalpies= -823.936833a.u
Sum of electronic and thermal Free Energies= -824.006121a.u

Excitation energy

Excited State 1: Singlet-A 3.0967 eV 400.37 nm f=1.4240

(Corrected Linear Response: 377.99 nm)

HOMO -> LUMO 94.9%
Excited State 2: Singlet-A 5.2564 eV 235.87 nm f=0.1637
HOMO-1 -> LUMO 68.1%
HOMO -> LUMO+3 2.9%
HOMO -> LUMO+4 3.1%
HOMO -> LUMO+17 8.4%
HOMO -> LUMO+18 4.8%
Excited State 3: Singlet-A 5.4712 eV 226.61 nm f=0.0013
HOMO-1 -> LUMO+15 15.2%
HOMO-1 -> LUMO+18 2.0%
HOMO -> LUMO+9 2.5%
HOMO -> LUMO+11 2.2%
HOMO -> LUMO+13 4.8%
HOMO -> LUMO+15 36.5%
HOMO -> LUMO+17 2.0%
HOMO -> LUMO+18 5.6%
HOMO -> LUMO+21 3.9%
Excited State 4: Singlet-A 5.5252 eV 224.40 nm f=0.0104
HOMO -> LUMO+1 50.9%
HOMO -> LUMO+3 15.9%
HOMO -> LUMO+4 2.3%
HOMO -> LUMO+5 10.2%

HOMO -> LUMO+17 2.2%
HOMO -> LUMO+24 2.1%
Excited State 5: Singlet-A 5.9648 eV 207.86 nm f=0.0161
HOMO-1 -> LUMO 14.1%
HOMO -> LUMO+2 2.5%
HOMO -> LUMO+3 7.1%
HOMO -> LUMO+4 36.4%
HOMO -> LUMO+7 2.1%
HOMO -> LUMO+9 5.2%
HOMO -> LUMO+12 6.6%
HOMO -> LUMO+17 3.2%
HOMO -> LUMO+28 2.8%
Excited State 6: Singlet-A 6.0608 eV 204.57 nm f=0.0144
HOMO-1 -> LUMO+1 3.1%
HOMO -> LUMO+1 13.0%
HOMO -> LUMO+3 33.2%
HOMO -> LUMO+4 11.6%
HOMO -> LUMO+7 2.8%
HOMO -> LUMO+8 10.6%
HOMO -> LUMO+10 3.0%
HOMO -> LUMO+23 2.2%
HOMO -> LUMO+27 2.8%

9.4. Computed xyz-coordinates (LC-ωHPBE6-311++G** IEFPCM, dichloromethane) S_1 and emission energies (LC-ωHPBE/6-311++G**) of merocyanine 6a

#p LC-ωHPBE 6-311++G** opt freq SCRF=(solvent=dichloromethane) TD=(singlet, n states=6,root=1)
6a
C 2.383311 -0.361222 -0.173318
C 1.672381 2.042054 0.128091
C -0.027450 0.190137 -0.142030
C 0.282986 1.637714 -0.378894
C 1.042885 -0.726630 -0.049440
C 2.715949 1.076122 -0.441836
C 3.417361 -1.326878 -0.081040
C 1.988052 3.455680 -0.347901
C 1.702453 2.002733 1.655253
C 3.146114 -2.695255 0.128888
N 2.903819 -3.811851 0.302497
C 4.777696 -0.974928 -0.200906
N 5.891979 -0.683817 -0.301169
C -1.333918 -0.308541 -0.068827
C -2.495525 0.455404 -0.218410
N -3.729275 -0.026823 -0.136279
C -4.064091 -1.423611 0.115169
C -5.574916 -1.473492 -0.073610
C -6.025548 -0.064465 0.294859
C -4.921774 0.803649 -0.285847

H 0.217701 1.848317 -1.454991
H -0.462888 2.275258 0.105771
H 0.798771 -1.771130 0.125239
H 2.790604 1.224850 -1.527242
H 3.699582 1.322798 -0.031893
H 2.973985 3.772206 0.002407
H 1.251421 4.167404 0.034530
H 1.981779 3.514193 -1.439654
H 2.690170 2.287522 2.026982
H 1.473160 1.005856 2.039068
H 0.971234 2.701160 2.070827
H -1.446022 -1.374028 0.107403
H -2.430851 1.521136 -0.412029
H -3.768647 -1.691496 1.136616
H -3.526259 -2.077520 -0.576192
H -5.811944 -1.682719 -1.119455
H -6.036691 -2.247053 0.538952
H -6.059456 0.053155 1.380637
H -7.005052 0.191797 -0.107669
H -4.787893 1.754609 0.232998
H -5.094162 1.010289 -1.348868
Low frequencies --- -10.3466 -7.5243 -0.0003 0.0003 0.0004 6.1678
Low frequencies --- 25.8153 33.5916 44.9027
Zero-point correction 0.351932 (Hartree/Particle)
Sum of electronic and zero-point Energies= -823.857321a.u
Sum of electronic and thermal Energies= -823.837394a.u
Sum of electronic and thermal Enthalpies= -823.836449a.u
Sum of electronic and thermal Free Energies= -823.907116a.u

Emission energy

Excited State 1: Singlet-A 5.2081 eV 238.06 nm f=0.1512

HOMO-1 -> LUMO 61.4%

corrected external emission excitation: 3.1000 eV 399.95 nm

9.5. Computed xyz-coordinates (LC-ωB97XD/6-311++G** IEFPCM, dichloromethane) and excitation energies (LC-ωB97XD/6-311++G**) of merocyanine 6b

#p LC-ωB97XD 6-311++G** opt freq SCRF=(solvent=dichloromethane)
6b
C -3.080160 0.489283 -0.171185
C -1.974646 -1.795284 -0.233300
C -0.624907 0.364138 -0.285561
C -0.732564 -1.064176 -0.757281
C -1.804439 1.065133 -0.068162
C -3.208951 -0.953905 -0.577467

C	-4.240737	1.225875	0.073395
C	-2.082247	-3.156777	-0.924057
C	-1.867574	-2.009333	1.282874
C	-4.189445	2.601455	0.412751
N	-4.132039	3.722538	0.690742
C	-5.530587	0.646653	-0.018865
N	-6.584709	0.176351	-0.095473
C	0.597193	1.069407	-0.150633
C	1.901878	0.594334	-0.094103
N	2.944230	1.433749	-0.129081
C	2.811649	2.876428	-0.353500
C	4.253399	3.361067	-0.481406
C	5.021325	2.394037	0.420864
C	4.342812	1.056238	0.143301
C	2.243988	-0.845292	0.078203
C	2.815951	-3.548625	0.404204
C	1.961747	-1.477277	1.287505
C	2.832167	-1.563571	-0.960936
C	3.108491	-2.915254	-0.800084
C	2.249672	-2.826941	1.449751
H	-0.778170	-1.021915	-1.854419
H	0.153094	-1.643362	-0.514669
H	-1.733777	2.107700	0.225491
H	-3.363213	-0.970223	-1.664964
H	-4.104416	-1.397365	-0.133224
H	-2.971654	-3.695238	-0.583339
H	-1.206618	-3.772471	-0.696664
H	-2.147341	-3.045707	-2.010687
H	-2.746564	-2.543134	1.655857
H	-1.790402	-1.063961	1.826109
H	-0.981722	-2.606553	1.518481
H	0.480975	2.143486	-0.057033
H	2.310784	3.341903	0.503735
H	2.209594	3.065635	-1.245105
H	4.591717	3.261318	-1.516433
H	4.360153	4.404821	-0.186701
H	4.888213	2.670319	1.470584
H	6.089808	2.360743	0.208162
H	4.396595	0.360558	0.980700
H	4.777135	0.569994	-0.735713
H	3.030682	-4.603950	0.527456
H	1.498812	-0.914233	2.090034
H	3.047947	-1.069784	-1.902102
H	3.548565	-3.476040	-1.616548
H	2.023340	-3.316689	2.389820

Low frequencies --- -8.6522 -4.8386 -0.6476 -0.0004 0.0005 0.0005

Low frequencies --- 14.1761 31.1263 48.0156

Zero-point correction 0.431349 (Hartree/Particle)

Sum of electronic and zero-point Energies= -1055.217942a.u

Sum of electronic and thermal Energies= -1055.193706a.u
Sum of electronic and thermal Enthalpies= -1055.192762a.u
Sum of electronic and thermal Free Energies= -1055.273313a.u

Excitation energy

Excited State 1: Singlet-A 2.9249 eV 423.90 nm f=1.3527

(Corrected Linear Response: 399.64 nm)

HOMO -> LUMO 96.6%
Excited State 2: Singlet-A 4.7649 eV 260.20 nm f=0.0274
HOMO-1 -> LUMO 14.8%
HOMO-1 -> LUMO+1 3.5%
HOMO -> LUMO+1 71.7%
Excited State 3: Singlet-A 4.9647 eV 249.73 nm f=0.0017
HOMO-1 -> LUMO+12 11.7%
HOMO -> LUMO+11 8.7%
HOMO -> LUMO+12 54.7%
HOMO -> LUMO+13 4.4%
HOMO -> LUMO+17 2.6%
Excited State 4: Singlet-A 4.9901 eV 248.46 nm f=0.1306
HOMO-2 -> LUMO 7.3%
HOMO-1 -> LUMO 59.1%
HOMO-1 -> LUMO+1 2.2%
HOMO -> LUMO+1 13.6%
HOMO -> LUMO+3 4.7%
Excited State 5: Singlet-A 5.0854 eV 243.80 nm f=0.0026
HOMO -> LUMO+2 51.4%
HOMO -> LUMO+4 4.5%
HOMO -> LUMO+5 5.0%
HOMO -> LUMO+6 15.9%
HOMO -> LUMO+7 5.8%
Excited State 6: Singlet-A 5.1555 eV 240.49 nm f=0.1069
HOMO-2 -> LUMO 74.8%
HOMO-2 -> LUMO+1 2.4%
HOMO-1 -> LUMO 9.0%

9.6. Computed xyz-coordinates (LC-ωB97XD/6-311++G** IEFPCM, dichloromethane) S_1 and emission energies (LC-ωB97XD/6-311++G**) of merocyanine 6b

#p LC-ωB97XD 6-311++G** opt freq SCRF=(solvent=dichloromethane) TD=(singlet, n states=6,root=1)

6b

C -3.084956 0.457817 -0.130895
C -1.960939 -1.810737 -0.355364
C -0.607544 0.331479 -0.263218
C -0.734367 -1.037779 -0.874344

C	-1.813724	1.021976	0.014340
C	-3.220485	-0.964096	-0.604147
C	-4.254956	1.225430	0.119833
C	-2.082842	-3.129091	-1.122427
C	-1.809721	-2.109621	1.141899
C	-4.193789	2.568870	0.547065
N	-4.117897	3.671783	0.897914
C	-5.546278	0.687652	-0.062180
N	-6.604259	0.236533	-0.214616
C	0.587556	1.026486	-0.010534
C	1.925558	0.566583	-0.007680
N	2.935618	1.451281	-0.139801
C	2.740065	2.864591	-0.469469
C	4.158853	3.397787	-0.646330
C	4.961035	2.548208	0.340488
C	4.341861	1.163536	0.174568
C	2.275321	-0.857403	0.166506
C	2.871208	-3.566641	0.499075
C	1.795701	-1.556983	1.278018
C	3.063010	-1.529560	-0.774061
C	3.352119	-2.877250	-0.610502
C	2.095106	-2.901722	1.443974
H	-0.843156	-0.905765	-1.961071
H	0.160245	-1.640401	-0.728547
H	-1.739281	2.042593	0.379975
H	-3.440517	-0.953552	-1.681147
H	-4.082189	-1.441362	-0.125141
H	-2.965265	-3.688495	-0.796976
H	-1.202661	-3.756703	-0.951712
H	-2.171070	-2.952561	-2.198877
H	-2.681107	-2.658722	1.511064
H	-1.712949	-1.194803	1.733247
H	-0.921133	-2.721422	1.320549
H	0.458773	2.085524	0.196752
H	2.235768	3.378834	0.359796
H	2.115107	2.963263	-1.360545
H	4.503144	3.220119	-1.668853
H	4.219595	4.467253	-0.445215
H	4.808094	2.908740	1.361443
H	6.031587	2.542066	0.134901
H	4.407657	0.537382	1.066880
H	4.817257	0.624149	-0.651737
H	3.098613	-4.618930	0.625407
H	1.176718	-1.042180	2.004223
H	3.419547	-1.002792	-1.652103
H	3.947877	-3.393178	-1.354828
H	1.718356	-3.433432	2.310336

Low frequencies --- -4.9495 -3.6200 -0.0006 -0.0006 0.0003 4.6965

Low frequencies --- 19.1080 34.3906 42.6465

Zero-point correction 0.429709 (Hartree/Particle)
Sum of electronic and zero-point Energies= -1055.122610a.u
Sum of electronic and thermal Energies= -1055.098270a.u
Sum of electronic and thermal Enthalpies= -1055.097326a.u
Sum of electronic and thermal Free Energies= -1055.177504a.u

Emission energy

Excited State 1: Singlet-A 2.8966 eV 428.03 nm f=1.1160

HOMO -> LUMO 95.8%

corrected external emission excitation: 2.9542 eV 419.69 nm

9.7. Computed xyz-coordinates (LC-ωHPBE/6-311++G** IEFPCM, dichloromethane) and excitation energies (LC-ωHPBE/6-311++G**) of merocyanine 6b

#p LC-ωHPBE 6-311++G** opt freq SCRF=(solvent=dichloromethane)

6b

C	-3.097985	0.411193	-0.166360
C	-2.042580	-1.824356	0.298018
C	-0.649600	0.279697	-0.045930
C	-0.718336	-1.216509	-0.165637
C	-1.826053	0.998949	-0.034807
C	-3.187682	-1.069454	-0.369973
C	-4.256608	1.166962	-0.156825
C	-2.092630	-3.288427	-0.126493
C	-2.166255	-1.741341	1.819410
C	-4.222168	2.579713	-0.013831
N	-4.187274	3.724452	0.103917
C	-5.540052	0.579111	-0.306233
N	-6.584602	0.109884	-0.428985
C	0.564522	1.017697	0.023826
C	1.878017	0.595748	-0.021468
N	2.886585	1.476176	0.003158
C	2.688396	2.923890	0.040921
C	4.087032	3.495981	-0.145771
C	4.989079	2.411688	0.427064
C	4.312871	1.131716	-0.033524
C	2.301077	-0.831585	-0.111538
C	3.118977	-3.477826	-0.282350
C	2.560508	-1.556466	1.045564
C	2.466876	-1.428779	-1.352718
C	2.872015	-2.751887	-1.436413
C	2.965690	-2.877607	0.958844
H	-0.551656	-1.469039	-1.220059
H	0.095397	-1.682522	0.386855
H	-1.768584	2.078438	0.060889

H -3.156161 -1.247657 -1.452087
H -4.149850 -1.449769 -0.019195
H -3.028410 -3.753045 0.194911
H -1.267876 -3.848160 0.322231
H -2.019060 -3.388503 -1.212631
H -3.113925 -2.174542 2.149563
H -2.123104 -0.710631 2.177844
H -1.357253 -2.299451 2.297526
H 0.415695 2.088896 0.091997
H 2.254664 3.212453 1.004401
H 2.000695 3.236869 -0.747982
H 4.294474 3.631917 -1.209990
H 4.202763 4.461288 0.346261
H 4.991630 2.457891 1.519175
H 6.020055 2.479055 0.080431
H 4.526512 0.279866 0.609547
H 4.609209 0.873046 -1.055423
H 3.434013 -4.513510 -0.349176
H 2.433579 -1.086059 2.015369
H 2.268839 -0.860179 -2.255487
H 2.993837 -3.217082 -2.408559
H 3.160892 -3.441794 1.864300
Low frequencies --- -4.2368 -2.4854 -0.0007 -0.0005 0.0003 1.5423
Low frequencies --- 10.4094 32.5822 46.1911
Zero-point correction 0.435226 (Hartree/Particle)
Sum of electronic and zero-point Energies= -1054.796123a.u
Sum of electronic and thermal Energies= -1054.771872a.u
Sum of electronic and thermal Enthalpies= -1054.770928a.u
Sum of electronic and thermal Free Energies= -1054.851901a.u

Excitation energy

Excited State 1: Singlet-A 3.0225 eV 410.20 nm f=1.4075

(Corrected Linear Response: 388.35 nm)

HOMO -> LUMO 94.9%
Excited State 2: Singlet-A 5.2266 eV 237.22 nm f=0.2165
HOMO-1 -> LUMO 66.9%
HOMO -> LUMO+20 11.3%
Excited State 3: Singlet-A 5.4354 eV 228.11 nm f=0.0029
HOMO-1 -> LUMO+16 2.5%
HOMO-1 -> LUMO+19 4.1%
HOMO-1 -> LUMO+21 10.5%
HOMO -> LUMO+12 2.8%
HOMO -> LUMO+16 8.2%
HOMO -> LUMO+18 2.8%
HOMO -> LUMO+19 12.5%
HOMO -> LUMO+21 25.9%
Excited State 4: Singlet-A 5.4740 eV 226.50 nm f=0.0077

HOMO-2 -> LUMO+6 2.6%
HOMO -> LUMO+1 30.1%
HOMO -> LUMO+2 10.0%
HOMO -> LUMO+4 6.9%
HOMO -> LUMO+5 12.3%
HOMO -> LUMO+7 8.1%
HOMO -> LUMO+21 4.0%
Excited State 5: Singlet-A 5.5108 eV 224.98 nm f=0.0021
HOMO-3 -> LUMO+2 5.2%
HOMO-3 -> LUMO+4 6.6%
HOMO-3 -> LUMO+5 13.3%
HOMO-2 -> LUMO+6 20.9%
HOMO -> LUMO+1 4.4%
HOMO -> LUMO+2 3.9%
HOMO -> LUMO+4 21.2%
HOMO -> LUMO+5 6.4%
Excited State 6: Singlet-A 5.5991 eV 221.44 nm f=0.0015
HOMO-3 -> LUMO+2 3.6%
HOMO-3 -> LUMO+4 4.6%
HOMO-3 -> LUMO+5 9.1%
HOMO-2 -> LUMO+6 16.5%
HOMO-1 -> LUMO+5 2.4%
HOMO -> LUMO+2 13.0%
HOMO -> LUMO+4 17.1%

9.8. Computed xyz-coordinates (LC-ωHPBE6-311++G** IEFPCM, dichloromethane) S_1 and emission energies (LC-ωHPBE/6-311++G**) of merocyanine 6b

#p LC-ωHPBE 6-311++G** opt freq SCRF=(solvent=dichloromethane) TD=(singlet, n states=6,root=1)
6b

C	-3.103509	0.416815	-0.133872
C	-1.998586	-1.853619	-0.242367
C	-0.627326	0.268328	-0.212899
C	-0.747857	-1.127537	-0.751610
C	-1.831357	0.971716	0.019888
C	-3.234315	-1.011859	-0.570974
C	-4.265995	1.196327	0.073857
C	-2.113889	-3.201551	-0.945635
C	-1.900220	-2.075986	1.265908
C	-4.200661	2.552368	0.460300
N	-4.128072	3.661147	0.776787
C	-5.560039	0.664209	-0.109585
N	-6.618487	0.225719	-0.261364
C	0.559809	0.987544	-0.001517
C	1.906563	0.571823	-0.017899
N	2.884917	1.486161	-0.115736
C	2.651841	2.905671	-0.370102
C	4.048702	3.471385	-0.572354

C	4.899498	2.594705	0.336469
C	4.305295	1.212390	0.120297
C	2.319520	-0.844359	0.109003
C	3.078555	-3.506388	0.373187
C	2.007142	-1.551856	1.265988
C	3.019016	-1.479384	-0.913593
C	3.389756	-2.806720	-0.783276
C	2.389592	-2.876015	1.398221
H	-0.803556	-1.061751	-1.846991
H	0.131111	-1.729336	-0.526386
H	-1.756551	2.005080	0.348033
H	-3.407652	-1.032529	-1.654980
H	-4.118302	-1.467149	-0.113840
H	-3.011805	-3.734016	-0.620806
H	-1.248923	-3.831072	-0.720346
H	-2.168840	-3.075990	-2.030434
H	-2.782269	-2.607098	1.633378
H	-1.825250	-1.131290	1.810001
H	-1.018835	-2.675603	1.507502
H	0.410615	2.046931	0.187348
H	2.167152	3.367490	0.499181
H	1.996111	3.033918	-1.234315
H	4.356085	3.348687	-1.613836
H	4.099275	4.531243	-0.324597
H	4.774781	2.897654	1.379103
H	5.962125	2.624073	0.096556
H	4.425210	0.541221	0.972372
H	4.750031	0.729850	-0.756033
H	3.372684	-4.545401	0.474786
H	1.459618	-1.058243	2.062051
H	3.251630	-0.938231	-1.824863
H	3.921784	-3.298681	-1.590279
H	2.146050	-3.418994	2.304979

Low frequencies --- -9.4002 -4.8716 -3.5745 -0.0003 0.0003 0.0005

Low frequencies --- 15.2342 29.4135 40.6031

Zero-point correction 0.434284 (Hartree/Particle)

Sum of electronic and zero-point Energies= -1054.698267a.u

Sum of electronic and thermal Energies= -1054.673820a.u

Sum of electronic and thermal Enthalpies= -1054.672876a.u

Sum of electronic and thermal Free Energies= -1054.754106a.u

Emission energy

Excited State 1: Singlet-A 2.9564 eV 419.37 nm f=1.1904

HOMO -> LUMO 93.5%

corrected external emission excitation: 2.9858 eV 415.25 nm

9.9. Computed xyz-coordinates (LC-ωB97XD/6-311++G** IEFPCM, dichloromethane) and excitation energies (LC-ωB97XD/6-311++G**) of merocyanine 6f

#p LC-ωB97XD 6-311++G** opt freq SCRF=(solvent=dichloromethane)

6f

C	-3.735662	-0.724239	-0.158299
C	-1.672167	-2.143551	0.174113
C	-1.547840	0.401371	-0.062531
C	-0.853265	-0.922052	-0.264256
C	-2.933529	0.420952	-0.011625
C	-3.073523	-2.044407	-0.436900
C	-5.125788	-0.660589	-0.091234
C	-0.983314	-3.410723	-0.338772
C	-1.758468	-2.204522	1.705706
C	-5.803995	0.566298	0.126771
N	-6.343465	1.572807	0.306979
C	-5.939379	-1.810865	-0.250363
N	-6.606502	-2.746450	-0.380699
C	-0.875272	1.649422	0.033843
C	0.477871	1.947431	-0.011873
N	0.916280	3.213357	0.011063
C	0.025469	4.378261	0.023699
C	0.964348	5.566347	-0.179121
C	2.283172	5.080588	0.423002
C	2.333219	3.616086	0.002153
C	1.550067	0.912592	-0.091667
C	3.506185	-1.057516	-0.238905
C	2.081637	0.369667	1.076561
C	2.017329	0.489390	-1.332379
C	2.989205	-0.496787	-1.405037
C	3.054771	-0.614944	1.004865
C	4.540000	-2.125281	-0.369608
O	4.969339	-2.524504	-1.427001
O	4.940173	-2.595988	0.810289
C	5.932352	-3.629295	0.783482
H	-0.613717	-1.006509	-1.332634
H	0.099593	-0.944066	0.259662
H	-3.427002	1.375676	0.139791
H	-3.001304	-2.146809	-1.528050
H	-3.697582	-2.871428	-0.088125
H	-1.537724	-4.303512	-0.034796
H	0.030228	-3.488000	0.066700
H	-0.912521	-3.408769	-1.430640
H	-2.339056	-3.076438	2.020769
H	-2.232040	-1.313482	2.125700
H	-0.757253	-2.290726	2.138395
H	-1.541243	2.499235	0.126775
H	-0.497840	4.431904	0.985080
H	-0.722607	4.293583	-0.768122

H 1.089167 5.764180 -1.247130
H 0.582236 6.471885 0.291660
H 2.252096 5.153101 1.513768
H 3.150250 5.636968 0.067254
H 2.918461 2.996653 0.680339
H 2.744688 3.505234 -1.006929
H 1.715847 0.704662 2.040465
H 1.604647 0.916398 -2.239153
H 3.350369 -0.844470 -2.365124
H 3.459241 -1.045796 1.911375
H 5.557479 -4.500187 0.244872
H 6.121360 -3.878081 1.824542
H 6.843411 -3.267080 0.306114
Low frequencies --- -5.2128 -0.0005 -0.0002 0.0007 2.6886 5.1373
Low frequencies --- 8.8536 19.7480 27.8162
Zero-point correction 0.474371 (Hartree/Particle)
Sum of electronic and zero-point Energies= -1283.050665a.u
Sum of electronic and thermal Energies= -1283.021741a.u
Sum of electronic and thermal Enthalpies= -1283.020797a.u
Sum of electronic and thermal Free Energies= -1283.113561a.u

Excitation energy

Excited State 1: Singlet-A 2.9622 eV 418.55 nm f=1.4015
(Corrected Linear Response: 394.98 nm)
HOMO -> LUMO 96.7%
Excited State 2: Singlet-A 4.2309 eV 293.04 nm f=0.0005
HOMO-1 -> LUMO+1 5.1%
HOMO -> LUMO+1 89.9%
Excited State 3: Singlet-A 4.9882 eV 248.55 nm f=0.1837
HOMO-1 -> LUMO 67.4%
HOMO -> LUMO+2 3.7%
HOMO -> LUMO+3 3.0%
HOMO -> LUMO+11 3.0%
HOMO -> LUMO+12 6.8%
HOMO -> LUMO+15 2.6%
Excited State 4: Singlet-A 5.0154 eV 247.21 nm f=0.0231
HOMO-1 -> LUMO 8.3%
HOMO-1 -> LUMO+12 11.0%
HOMO -> LUMO+10 3.6%
HOMO -> LUMO+12 47.3%
HOMO -> LUMO+13 4.8%
HOMO -> LUMO+16 5.7%
Excited State 5: Singlet-A 5.0970 eV 243.25 nm f=0.0058
HOMO-3 -> LUMO+1 6.9%
HOMO-1 -> LUMO 8.0%
HOMO-1 -> LUMO+2 4.7%
HOMO -> LUMO+2 69.2%

HOMO -> LUMO+3 3.4%

Excited State 6: Singlet-A 5.1179 eV 242.26 nm f=0.0129

HOMO-1 -> LUMO 3.3%

HOMO -> LUMO+2 8.8%

HOMO -> LUMO+3 37.5%

HOMO -> LUMO+4 11.7%

HOMO -> LUMO+5 2.5%

HOMO -> LUMO+6 11.4%

HOMO -> LUMO+8 7.8%

9.10. Computed xyz-coordinates (LC-ωB97XD/6-311++G** IEFPCM, dichloromethane) S_1 and emission energies (LC-ωB97XD/6-311++G**) of merocyanine 6f

#p LC-ωB97XD 6-311++G** opt freq SCRF=(solvent=dichloromethane) TD=(singlet, n states=6,root=1)

6f

C	-3.686298	-0.712219	-0.090697
C	-1.587691	-1.995944	-0.730788
C	-1.523168	0.494583	-0.247373
C	-0.981216	-0.627974	-1.091961
C	-2.876913	0.401614	0.156953
C	-3.119001	-1.895937	-0.826669
C	-5.046997	-0.723692	0.303680
C	-1.089787	-3.042911	-1.729520
C	-1.171601	-2.412500	0.686107
C	-5.644314	0.367669	0.972292
N	-6.113395	1.274948	1.520244
C	-5.887219	-1.825561	0.032698
N	-6.570192	-2.734535	-0.194516
C	-0.837999	1.662752	0.110235
C	0.544750	1.974581	-0.000120
N	0.921390	3.266763	-0.099538
C	-0.019623	4.369134	-0.319519
C	0.881541	5.590072	-0.475991
C	2.055770	5.255460	0.445261
C	2.274167	3.768389	0.182321
C	1.583640	0.937921	0.023189
C	3.514527	-1.090224	0.077970
C	1.543811	-0.061433	1.005981
C	2.612970	0.907302	-0.929754
C	3.560347	-0.099630	-0.904848
C	2.498403	-1.060948	1.036770
C	4.553880	-2.153255	0.058429
O	5.451457	-2.211648	-0.751784
O	4.391706	-3.046625	1.035737
C	5.347721	-4.111048	1.093677
H	-1.234990	-0.401520	-2.137697
H	0.104677	-0.690780	-1.048990

H -3.299620 1.238173 0.706455
H -3.409922 -1.816020 -1.883584
H -3.571123 -2.822486 -0.457820
H -1.528386 -4.022525 -1.516803
H -0.001261 -3.140780 -1.674134
H -1.354786 -2.767686 -2.754914
H -1.611100 -3.380998 0.942431
H -1.494839 -1.687471 1.438263
H -0.084242 -2.505235 0.752472
H -1.466091 2.459117 0.501627
H -0.672752 4.489052 0.555044
H -0.648101 4.163154 -1.189109
H 1.222775 5.677519 -1.511105
H 0.367675 6.512028 -0.204477
H 1.775397 5.416789 1.489836
H 2.951141 5.840548 0.234787
H 2.706753 3.224322 1.024893
H 2.928265 3.626414 -0.683977
H 0.750177 -0.050374 1.743902
H 2.641344 1.649094 -1.718647
H 4.340053 -0.136196 -1.655969
H 2.457559 -1.824250 1.803102
H 5.316625 -4.699458 0.175977
H 5.054432 -4.719882 1.945131
H 6.352213 -3.711812 1.238406
Low frequencies --- -7.5190 -2.6067 -0.0006 -0.0004 0.0006 4.6154
Low frequencies --- 14.6202 23.3848 30.7865
Zero-point correction 0.472228 (Hartree/Particle)
Sum of electronic and zero-point Energies= -1282.956922a.u
Sum of electronic and thermal Energies= -1282.927926a.u
Sum of electronic and thermal Enthalpies= -1282.926982a.u
Sum of electronic and thermal Free Energies= -1283.018819a.u

Emission energy

Excited State 1: Singlet-A 2.7436 eV 451.90 nm f=0.9774

HOMO -> LUMO 91.7%

corrected external emission excitation: 2.8074eV 441.63 nm

9.11. Computed xyz-coordinates (LC-ωHPBE/6-311++G** IEFPCM, dichloromethane) and excitation energies (LC-ωHPBE/6-311++G**) of merocyanine 6f

#p LC-ωHPBE 6-311++G** opt freq SCRF=(solvent=dichloromethane)
6f
C -3.775740 -0.714330 -0.161180
C -1.756999 -2.153655 0.256746

C	-1.577820	0.370923	-0.037172
C	-0.902388	-0.963519	-0.180978
C	-2.953282	0.421181	-0.015208
C	-3.127682	-2.041654	-0.403867
C	-5.153894	-0.624165	-0.131517
C	-1.084134	-3.443425	-0.201375
C	-1.900823	-2.178269	1.778431
C	-5.815294	0.620340	0.050295
N	-6.345461	1.631131	0.199404
C	-5.987013	-1.762577	-0.295440
N	-6.669575	-2.680193	-0.429060
C	-0.879771	1.610703	0.042513
C	0.469050	1.889287	-0.004784
N	0.919486	3.151143	0.017480
C	0.037330	4.316070	0.041870
C	0.977406	5.496917	-0.157197
C	2.291749	5.001287	0.429573
C	2.332319	3.547965	-0.011257
C	1.539786	0.854150	-0.093519
C	3.554872	-1.042515	-0.258817
C	2.111274	0.342589	1.065484
C	1.989942	0.427051	-1.334711
C	2.992487	-0.522131	-1.415676
C	3.114453	-0.604953	0.984549
C	4.630408	-2.064562	-0.399962
O	5.042770	-2.467238	-1.460716
O	5.092425	-2.490061	0.772242
C	6.128997	-3.473521	0.719789
H	-0.628264	-1.081038	-1.236561
H	0.032224	-0.981000	0.376704
H	-3.432402	1.387983	0.100577
H	-3.016481	-2.150816	-1.489902
H	-3.776918	-2.855467	-0.072920
H	-1.670493	-4.314652	0.101740
H	-0.089156	-3.537444	0.241373
H	-0.975243	-3.468652	-1.288840
H	-2.515095	-3.027003	2.089851
H	-2.365547	-1.266788	2.160567
H	-0.920862	-2.281626	2.251253
H	-1.534335	2.470800	0.119331
H	-0.483055	4.366853	1.004408
H	-0.714627	4.242739	-0.747166
H	1.096981	5.701536	-1.223930
H	0.603464	6.402132	0.320282
H	2.265022	5.057461	1.520858
H	3.159568	5.560239	0.080315
H	2.929676	2.920125	0.647817
H	2.728206	3.453444	-1.027776
H	1.760838	0.681520	2.034610

H 1.547853 0.830839 -2.239106
H 3.348300 -0.869888 -2.378527
H 3.556613 -1.008870 1.886769
H 5.774751 -4.368371 0.208712
H 6.374869 -3.698962 1.753955
H 7.000020 -3.076382 0.199085
Low frequencies --- -3.3448 -0.0002 0.0006 0.0009 1.4061 5.8064
Low frequencies --- 9.3689 20.9176 29.7790
Zero-point correction 0.478742 (Hartree/Particle)
Sum of electronic and zero-point Energies= -1282.558079a.u
Sum of electronic and thermal Energies= -1282.529277a.u
Sum of electronic and thermal Enthalpies= -1282.528333a.u
Sum of electronic and thermal Free Energies= -1282.620599a.u

Excitation energy

Excited State 1: Singlet-A 3.0402 eV 407.82 nm f=1.4231

(Corrected Linear Response: 387.02 nm)
HOMO -> LUMO 94.8%
Excited State 2: Singlet-A 4.9033 eV 252.86 nm f=0.0018
HOMO-1 -> LUMO+1 5.1%
HOMO -> LUMO+1 86.3%
Excited State 3: Singlet-A 5.2352 eV 236.83 nm f=0.2158
HOMO-1 -> LUMO 68.9%
HOMO -> LUMO+21 5.9%
HOMO -> LUMO+22 4.0%
HOMO -> LUMO+23 4.0%
Excited State 4: Singlet-A 5.2946 eV 234.17 nm f=0.0195
HOMO-3 -> LUMO+1 62.8%
HOMO-2 -> LUMO+1 3.7%
HOMO-2 -> LUMO+4 4.3%
HOMO-2 -> LUMO+6 19.8%
Excited State 5: Singlet-A 5.4683 eV 226.73 nm f=0.0003
HOMO-5 -> LUMO+1 2.2%
HOMO-4 -> LUMO+1 72.2%
HOMO-4 -> LUMO+31 4.0%
HOMO-4 -> LUMO+32 3.3%
HOMO-4 -> LUMO+35 2.9%
HOMO-4 -> LUMO+36 2.5%
Excited State 6: Singlet-A 5.4819 eV 226.17 nm f=0.0021
HOMO-1 -> LUMO+19 6.3%
HOMO-1 -> LUMO+21 2.3%
HOMO-1 -> LUMO+22 6.2%
HOMO-1 -> LUMO+24 2.8%
HOMO -> LUMO+13 2.3%
HOMO -> LUMO+15 2.8%
HOMO -> LUMO+17 3.0%

HOMO -> LUMO+19 16.4%
HOMO -> LUMO+21 6.8%
HOMO -> LUMO+22 13.4%
HOMO -> LUMO+23 2.2%
HOMO -> LUMO+24 5.2%

9.12. Computed xyz-coordinates (LC-ωHPBE6-311++G** IEFPCM, dichloromethane) S_1 and emission energies (LC-ωHPBE/6-311++G**) merocyanine 6f

#p LC-ωHPBE 6-311++G** opt freq SCRF=(solvent=dichloromethane) TD=(singlet, n states=6,root=1)

6f

C	-3.746157	-0.738603	-0.098971
C	-1.661958	-2.115844	-0.485731
C	-1.542568	0.394599	-0.223566
C	-0.984048	-0.811538	-0.921245
C	-2.917547	0.366280	0.104636
C	-3.172445	-1.987890	-0.698791
C	-5.124756	-0.679057	0.210093
C	-1.134515	-3.262164	-1.341662
C	-1.360893	-2.401446	0.984622
C	-5.722356	0.479580	0.752016
N	-6.193654	1.436300	1.195807
C	-5.985582	-1.773366	-0.021225
N	-6.688171	-2.670546	-0.212599
C	-0.865642	1.591398	0.052448
C	0.504149	1.918075	-0.033139
N	0.880510	3.206044	-0.078038
C	-0.053883	4.321800	-0.211284
C	0.850715	5.531240	-0.384919
C	2.071834	5.156230	0.443786
C	2.252709	3.681132	0.124248
C	1.576963	0.900362	-0.039225
C	3.586893	-1.019535	-0.017924
C	1.700958	0.024390	1.036059
C	2.470448	0.807955	-1.104301
C	3.463699	-0.151097	-1.095253
C	2.702838	-0.927231	1.050247
C	4.678404	-2.032344	-0.053786
O	5.467295	-2.141984	-0.961428
O	4.694295	-2.809440	1.025537
C	5.715696	-3.809069	1.066034
H	-1.143896	-0.677957	-1.999933
H	0.091728	-0.904611	-0.782003
H	-3.348567	1.258771	0.550337
H	-3.387009	-1.989011	-1.775526
H	-3.675234	-2.868924	-0.288372
H	-1.619614	-4.203829	-1.071278

H -0.057335 -3.386107 -1.202025
H -1.320063 -3.078562 -2.403425
H -1.831621 -3.337316 1.296992
H -1.730287 -1.605441 1.635827
H -0.283599 -2.495657 1.143581
H -1.511217 2.410240 0.357749
H -0.656830 4.416262 0.700463
H -0.730346 4.152443 -1.051950
H 1.125992 5.646255 -1.436203
H 0.368751 6.451268 -0.055677
H 1.861456 5.286731 1.508210
H 2.959251 5.738321 0.196431
H 2.736377 3.112069 0.920128
H 2.840169 3.551877 -0.790226
H 1.005530 0.093671 1.865376
H 2.368156 1.472186 -1.955443
H 4.153224 -0.240150 -1.926691
H 2.800193 -1.602616 1.891192
H 5.608349 -4.492760 0.224278
H 5.574535 -4.337694 2.004866
H 6.700641 -3.343798 1.035263
Low frequencies --- -5.2804 -4.9332 -0.0006 -0.0004 0.0005 3.4607
Low frequencies --- 14.2572 22.5481 29.1845
Zero-point correction 0.477773 (Hartree/Particle)
Sum of electronic and zero-point Energies= -1282.460415a.u
Sum of electronic and thermal Energies= -1282.431447a.u
Sum of electronic and thermal Enthalpies= -1282.430503a.u
Sum of electronic and thermal Free Energies= -1282.522777a.u

Emission energy

Excited State 1: Singlet-A 2.9181 eV 424.89 nm f=1.1851

HOMO -> LUMO 88.9%

HOMO -> LUMO+1 5.1%

corrected external emission excitation: 2.9460eV 420.86 nm

9.13. Computed xyz-coordinates (LC-ωB97XD/6-311++G** IEFPCM, dichloromethane) and excitation energies (LC-ωB97XD/6-311++G**) of merocyanine 6g

#p LC-ωB97XD 6-311++G** opt freq SCRF=(solvent=dichloromethane)
6g
C -3.449357 -0.016567 -0.195866
C -1.886915 -1.884281 0.479412
C -1.041624 0.475400 -0.031059
C -0.736839 -1.001793 -0.024172
C -2.370354 0.875766 -0.103015

C	-3.170464	-1.492381	-0.259312
C	-4.769867	0.428722	-0.269716
C	-1.554686	-3.347985	0.178216
C	-2.067184	-1.713105	1.994370
C	-5.089097	1.810241	-0.258874
N	-5.334444	2.940261	-0.247569
C	-5.860493	-0.469796	-0.376614
N	-6.752984	-1.200338	-0.465378
C	-0.056523	1.495453	0.001515
C	1.329521	1.403457	0.010818
N	2.095491	2.502518	0.005296
C	1.559184	3.867056	-0.043504
C	2.791122	4.747429	-0.250197
C	3.918273	3.935563	0.388385
C	3.568170	2.502519	0.004451
C	2.068429	0.109420	0.015674
C	3.384164	-2.354273	0.026745
C	2.476659	-0.467955	1.220438
C	2.346048	-0.545137	-1.174775
C	2.998820	-1.774952	-1.181002
C	3.124692	-1.687960	1.229588
O	4.013201	-3.546507	0.136738
C	4.288051	-4.275862	-1.048730
H	-0.480270	-1.289898	-1.052136
H	0.147775	-1.210590	0.574039
H	-2.584371	1.939853	-0.099799
H	-3.074044	-1.759453	-1.320323
H	-4.018932	-2.062712	0.128117
H	-2.352279	-4.007788	0.532484
H	-0.626229	-3.641609	0.677595
H	-1.428658	-3.512410	-0.896270
H	-2.883800	-2.345141	2.355514
H	-2.294904	-0.679125	2.265701
H	-1.154046	-2.007347	2.520278
H	-0.466577	2.498410	-0.010586
H	1.046907	4.093885	0.898184
H	0.836136	3.964614	-0.856782
H	2.980693	4.876760	-1.319168
H	2.664681	5.734871	0.193365
H	3.895996	4.044594	1.476391
H	4.908995	4.222404	0.035802
H	3.958450	1.764921	0.703276
H	3.937622	2.255847	-0.997066
H	2.265372	0.035334	2.157780
H	2.033686	-0.106424	-2.116164
H	3.191058	-2.264746	-2.126019
H	3.434224	-2.148260	2.160270
H	3.365346	-4.527117	-1.580789
H	4.781409	-5.191169	-0.728952

H 4.955329 -3.716625 -1.711741

Low frequencies --- -2.9867 -0.0004 -0.0002 0.0007 2.0672 4.6302

Low frequencies --- 7.8096 26.7823 31.8608

Zero-point correction 0.464212 (Hartree/Particle)

Sum of electronic and zero-point Energies= -1169.708077a.u

Sum of electronic and thermal Energies= -1169.681218a.u

Sum of electronic and thermal Enthalpies= -1169.680274a.u

Sum of electronic and thermal Free Energies= -1169.767461a.u

Excitation energy

Excited State 1: Singlet-A 2.9490 eV 420.43 nm f=1.3891

(Corrected Linear Response: 396.22 nm)

HOMO -> LUMO 96.8%

Excited State 2: Singlet-A 4.6559 eV 266.29 nm f=0.0102

HOMO-5 -> LUMO 2.6%

HOMO-1 -> LUMO 89.4%

HOMO-1 -> LUMO+16 2.1%

Excited State 3: Singlet-A 4.9606 eV 249.94 nm f=0.0178

HOMO-2 -> LUMO 6.5%

HOMO-2 -> LUMO+12 11.0%

HOMO -> LUMO+10 2.9%

HOMO -> LUMO+11 3.0%

HOMO -> LUMO+12 52.0%

HOMO -> LUMO+13 3.8%

HOMO -> LUMO+15 2.7%

HOMO -> LUMO+16 2.4%

Excited State 4: Singlet-A 4.9890 eV 248.51 nm f=0.1697

HOMO-2 -> LUMO 63.8%

HOMO -> LUMO+1 5.3%

HOMO -> LUMO+4 2.1%

HOMO -> LUMO+5 2.5%

HOMO -> LUMO+11 3.5%

HOMO -> LUMO+12 3.7%

HOMO -> LUMO+16 6.4%

Excited State 5: Singlet-A 5.0696 eV 244.56 nm f=0.0215

HOMO-2 -> LUMO 11.2%

HOMO-1 -> LUMO+2 8.9%

HOMO -> LUMO+1 34.7%

HOMO -> LUMO+4 13.2%

HOMO -> LUMO+6 7.5%

HOMO -> LUMO+8 4.6%

Excited State 6: Singlet-A 5.0957 eV 243.31 nm f=0.0314

HOMO-3 -> LUMO+3 6.5%

HOMO-3 -> LUMO+6 4.3%

HOMO-2 -> LUMO 2.5%

HOMO-1 -> LUMO+1 3.2%

HOMO-1 -> LUMO+2 64.0%
HOMO -> LUMO+1 4.0%
HOMO -> LUMO+4 3.6%

9.14. Computed xyz-coordinates (LC-ωB97XD/6-311++G** IEFPCM, dichloromethane) S_1 and emission energies (LC-ωB97XD/6-311++G**) of merocyanine 6g

#p LC-ωB97XD 6-311++G** opt freq SCRF=(solvent=dichloromethane) TD=(singlet, n states=6,root=1)

6g

C -3.396761 -0.049867 -0.216712
C -1.920241 -1.574339 1.162814
C -0.967825 0.444974 -0.047582
C -0.716055 -0.960612 0.426208
C -2.303745 0.811735 -0.347727
C -3.166348 -1.453644 0.269428
C -4.711493 0.380556 -0.546671
C -1.635286 -3.054332 1.428972
C -2.151448 -0.855692 2.498430
C -4.974438 1.677505 -1.035827
N -5.164421 2.749672 -1.435931
C -5.826948 -0.472114 -0.413022
N -6.738765 -1.181362 -0.303386
C 0.010716 1.441372 -0.218836
C 1.419466 1.343534 -0.141513
N 2.161638 2.462052 0.011348
C 1.589827 3.783735 0.287211
C 2.790683 4.736882 0.302934
C 3.993892 3.821120 0.534893
C 3.606010 2.561067 -0.225420
C 2.115287 0.043691 -0.204236
C 3.350324 -2.471288 -0.297713
C 2.940055 -0.390143 0.844987
C 1.935344 -0.801080 -1.296277
C 2.546619 -2.048448 -1.355796
C 3.542735 -1.629907 0.805396
O 3.980233 -3.665222 -0.245408
C 3.803751 -4.569289 -1.325051
H -0.472893 -1.598665 -0.434336
H 0.159862 -0.993766 1.078485
H -2.482447 1.825310 -0.696054
H -3.051631 -2.115599 -0.600719
H -4.043402 -1.817403 0.814435
H -2.476525 -3.525579 1.946397
H -0.745413 -3.170029 2.055383
H -1.464352 -3.597016 0.494082
H -2.999340 -1.298024 3.030019

H -2.359872 0.208402 2.359307
H -1.267179 -0.945351 3.136725
H -0.384350 2.431430 -0.422620
H 1.059461 3.750647 1.245144
H 0.868557 4.070600 -0.484536
H 2.882478 5.233226 -0.666239
H 2.683771 5.507760 1.065868
H 4.106524 3.589895 1.597862
H 4.930341 4.247238 0.174494
H 4.132419 1.671561 0.109965
H 3.785618 2.676520 -1.304031
H 3.080384 0.242754 1.714925
H 1.301477 -0.483903 -2.117141
H 2.384802 -2.674689 -2.222877
H 4.162536 -1.975214 1.624348
H 2.750886 -4.844073 -1.440860
H 4.383009 -5.454400 -1.071340
H 4.179537 -4.144893 -2.260974
Low frequencies --- -3.9526 -2.1083 0.0004 0.0006 0.0006 7.7307
Low frequencies --- 16.6291 26.3100 31.4611
Zero-point correction 0.462393 (Hartree/Particle)
Sum of electronic and zero-point Energies= -1169.611870a.u
Sum of electronic and thermal Energies= -1169.584771a.u
Sum of electronic and thermal Enthalpies= -1169.583827a.u
Sum of electronic and thermal Free Energies= -1169.671046a.u

Emission energy

Excited State 1: Singlet-A 2.9279 eV 423.45 nm f=1.1625

HOMO -> LUMO 96.1%

corrected external emission excitation: 2.9676 eV 417.80 nm

9.15. Computed xyz-coordinates (LC-ωHPBE/6-311++G** IEFPCM, dichloromethane) and excitation energies (LC-ωHPBE/6-311++G**) of merocyanine 6g

#p LC-ωHPBE 6-311++G** opt freq SCRF=(solvent=dichloromethane)
6g
C -3.469663 -0.057336 -0.199932
C -1.914626 -1.899716 0.517429
C -1.073617 0.444354 -0.014362
C -0.763095 -1.025521 0.019596
C -2.392317 0.841356 -0.101152
C -3.179222 -1.525440 -0.249433
C -4.779697 0.378952 -0.296114
C -1.579438 -3.362997 0.247622
C -2.125105 -1.704977 2.018977

C	-5.104728	1.761360	-0.301141
N	-5.361385	2.883798	-0.303463
C	-5.867369	-0.525337	-0.412720
N	-6.754778	-1.253123	-0.509685
C	-0.086858	1.467417	0.012916
C	1.291983	1.384727	0.020037
N	2.046618	2.490854	0.012956
C	1.492508	3.842949	-0.028413
C	2.707949	4.738765	-0.226453
C	3.839768	3.941350	0.406446
C	3.513890	2.513720	0.002568
C	2.054174	0.104598	0.018583
C	3.491328	-2.282323	0.008894
C	2.495776	-0.458870	1.214687
C	2.355046	-0.530407	-1.171628
C	3.068733	-1.722113	-1.188233
C	3.202934	-1.640029	1.213224
O	4.186710	-3.438224	0.106052
C	4.496455	-4.132426	-1.088879
H	-0.491877	-1.328421	-0.999025
H	0.116000	-1.215837	0.632746
H	-2.609808	1.904525	-0.114661
H	-3.054639	-1.793281	-1.306114
H	-4.031978	-2.100105	0.119483
H	-2.384441	-4.015320	0.595933
H	-0.663489	-3.651092	0.770054
H	-1.432605	-3.545338	-0.820290
H	-2.949550	-2.329254	2.373040
H	-2.356071	-0.667180	2.268611
H	-1.225657	-1.992712	2.569254
H	-0.501513	2.468401	-0.005544
H	0.974208	4.058161	0.912105
H	0.770236	3.936705	-0.842536
H	2.898400	4.876402	-1.293625
H	2.567710	5.722669	0.220312
H	3.808045	4.035389	1.495020
H	4.828333	4.249018	0.066234
H	3.922369	1.772227	0.686397
H	3.884030	2.292458	-1.004071
H	2.270827	0.030289	2.157178
H	2.021976	-0.101049	-2.111027
H	3.282745	-2.196738	-2.136939
H	3.543573	-2.089422	2.138964
H	3.587587	-4.430592	-1.618504
H	5.045667	-5.021760	-0.788413
H	5.124206	-3.525384	-1.746785

Low frequencies --- 0.0005 0.0006 0.0009 2.7543 3.7779 4.2244

Low frequencies --- 9.7372 29.1725 33.1082

Zero-point correction 0.468288 (Hartree/Particle)

Sum of electronic and zero-point Energies= -1169.253051a.u
Sum of electronic and thermal Energies= -1169.226271a.u
Sum of electronic and thermal Enthalpies= -1169.225327a.u
Sum of electronic and thermal Free Energies= -1169.312008a.u

Excitation energy

Excited State 1: Singlet-A 3.0099 eV 411.92 nm f=1.4125

(Corrected Linear Response: 389.79 nm)

HOMO -> LUMO 94.9%
Excited State 2: Singlet-A 5.2118 eV 237.89 nm f=0.2131
HOMO-2 -> LUMO 61.3%
HOMO-1 -> LUMO 6.9%
HOMO -> LUMO+21 2.6%
HOMO -> LUMO+22 8.2%
Excited State 3: Singlet-A 5.2295 eV 237.08 nm f=0.0329
HOMO-3 -> LUMO+9 6.3%
HOMO-3 -> LUMO+10 3.0%
HOMO-3 -> LUMO+14 2.3%
HOMO-1 -> LUMO+3 2.2%
HOMO-1 -> LUMO+5 65.9%
HOMO-1 -> LUMO+6 4.1%
Excited State 4: Singlet-A 5.3696 eV 230.90 nm f=0.0012
HOMO-5 -> LUMO 3.3%
HOMO-2 -> LUMO 4.1%
HOMO-1 -> LUMO 74.6%
HOMO-1 -> LUMO+22 2.8%
Excited State 5: Singlet-A 5.4194 eV 228.78 nm f=0.0020
HOMO-2 -> LUMO+18 2.7%
HOMO-2 -> LUMO+20 3.2%
HOMO-2 -> LUMO+21 7.0%
HOMO-2 -> LUMO+22 2.7%
HOMO -> LUMO+14 3.2%
HOMO -> LUMO+16 3.4%
HOMO -> LUMO+18 7.7%
HOMO -> LUMO+19 5.3%
HOMO -> LUMO+20 8.4%
HOMO -> LUMO+21 15.9%
HOMO -> LUMO+22 8.5%
HOMO -> LUMO+24 3.7%
HOMO -> LUMO+28 2.6%
Excited State 6: Singlet-A 5.4648 eV 226.88 nm f=0.0088
HOMO-2 -> LUMO 2.9%
HOMO -> LUMO+1 32.5%
HOMO -> LUMO+2 9.8%
HOMO -> LUMO+4 19.3%
HOMO -> LUMO+7 7.8%

HOMO -> LUMO+13 2.2%
HOMO -> LUMO+24 2.8%

9.16. Computed xyz-coordinates (LC-ωHPBE6-311++G** IEFPCM, dichloromethane) S_1 and emission energies (LC-ωHPBE/6-311++G**) of merocyanine 6g

#p LC-ωHPBE 6-311++G** opt freq SCRF=(solvent=dichloromethane) TD=(singlet, n states=6,root=1)

6g

C	-3.410304	-0.079504	-0.219438
C	-1.946058	-1.637654	1.114013
C	-0.987818	0.414042	-0.007404
C	-0.737682	-1.004984	0.412977
C	-2.319648	0.787535	-0.302111
C	-3.177023	-1.495283	0.214567
C	-4.715844	0.356426	-0.549540
C	-1.666005	-3.118837	1.344042
C	-2.194006	-0.956372	2.458828
C	-4.975062	1.668016	-1.001928
N	-5.167839	2.746004	-1.370495
C	-5.832841	-0.500665	-0.456963
N	-6.745849	-1.205554	-0.383423
C	-0.018123	1.420534	-0.142678
C	1.388213	1.341384	-0.075148
N	2.117265	2.464305	0.042839
C	1.536461	3.792509	0.240040
C	2.725483	4.746462	0.186202
C	3.916790	3.867864	0.541406
C	3.568896	2.551397	-0.127458
C	2.107428	0.050244	-0.142295
C	3.422766	-2.409224	-0.281499
C	2.876811	-0.409132	0.930813
C	2.015395	-0.741598	-1.275607
C	2.669462	-1.963129	-1.358469
C	3.520246	-1.622471	0.867723
O	4.092153	-3.582292	-0.252882
C	4.015973	-4.422901	-1.390678
H	-0.487511	-1.610357	-0.467347
H	0.129367	-1.061283	1.074110
H	-2.498625	1.813946	-0.611002
H	-3.048257	-2.123564	-0.676597
H	-4.057282	-1.882426	0.736239
H	-2.513647	-3.602593	1.836791
H	-0.786190	-3.252926	1.979022
H	-1.483285	-3.636605	0.398504
H	-3.048976	-1.409888	2.967254
H	-2.399139	0.110208	2.341615
H	-1.320430	-1.062755	3.107540

H -0.424842 2.410923 -0.319158
H 1.017868 3.818502 1.204261
H 0.806300 4.021654 -0.540602
H 2.840606 5.137961 -0.827077
H 2.596184 5.593119 0.859708
H 3.983517 3.726766 1.623146
H 4.866690 4.269616 0.189401
H 4.075699 1.695004 0.309370
H 3.804899 2.576101 -1.199402
H 2.950089 0.186505 1.835403
H 1.422254 -0.402339 -2.118657
H 2.579032 -2.550832 -2.262740
H 4.105796 -1.989540 1.702820
H 2.984368 -4.726389 -1.587482
H 4.611671 -5.302298 -1.157196
H 4.430534 -3.929417 -2.273691
Low frequencies --- -0.0008 -0.0004 0.0004 2.9174 3.6521 8.3968
Low frequencies --- 14.6263 30.4035 34.1888
Zero-point correction 0.467400 (Hartree/Particle)
Sum of electronic and zero-point Energies= -1169.155035a.u
Sum of electronic and thermal Energies= -1169.128002a.u
Sum of electronic and thermal Enthalpies= -1169.127058a.u
Sum of electronic and thermal Free Energies= -1169.214071a.u

Emission energy

Excited State 1: Singlet-A 2.9659 eV 418.04 nm f=1.2068

HOMO -> LUMO 93.8%

corrected external emission excitation: 2.9861 eV 415.20 nm

9.17. Calculated S1 Excitation Energies and Deviations from the Experimental Longest Wavelength Absorption Bands of Merocyanines 6a, 6b, 6f, and 6g

Table S5. Calculated vs experimental longest wavelength absorption bands (all energies and differences in eV)

	E_{exp}	E_{B3LYP}		$E_{LC\text{-}\omega B97XD}$		$E_{LC\text{-}\omega HPBE}$		$E_{B3LYP} - E_{exp}$		$E_{LC\text{-}\omega B97XD} - E_{exp}$		$E_{LC\text{-}\omega HPBE} - E_{exp}$	
		cLR	LR	cLR	LR	cLR	LR	cLR	LR	cLR	LR	cLR	LR
**6a**	2.515	3.134	2.937	3.226	3.030	3.280	3.097	0.619	0.422	0.711	0.515	0.765	0.582
**6b**	2.431	3.057	2.875	3.102	2.925	3.193	3.023	0.626	0.444	0.671	0.494	0.762	0.592
**6f**	2.445	2.921	2.873	3.139	2.962	3.204	3.040	0.476	0.428	0.694	0.517	0.759	0.595
**6g**	2.417	3.050	2.868	3.129	2.949	3.181	3.010	0.633	0.451	0.712	0.532	0.764	0.593

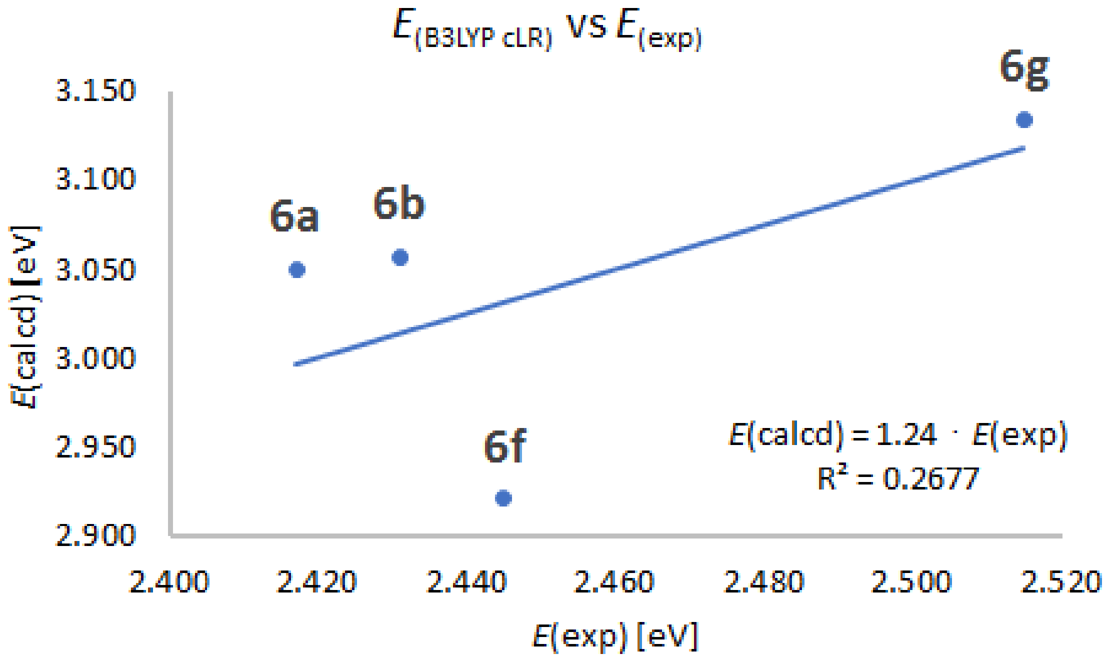

Figure S40. Plot of the calculated excitation energies (B3LYP cLR) of merocyanines **6a**, **6b**, **6f**, and **6g** $E_{\text{(B3LYP cLR)}}$ vs $E_{\text{(exp)}}$, linear regression.

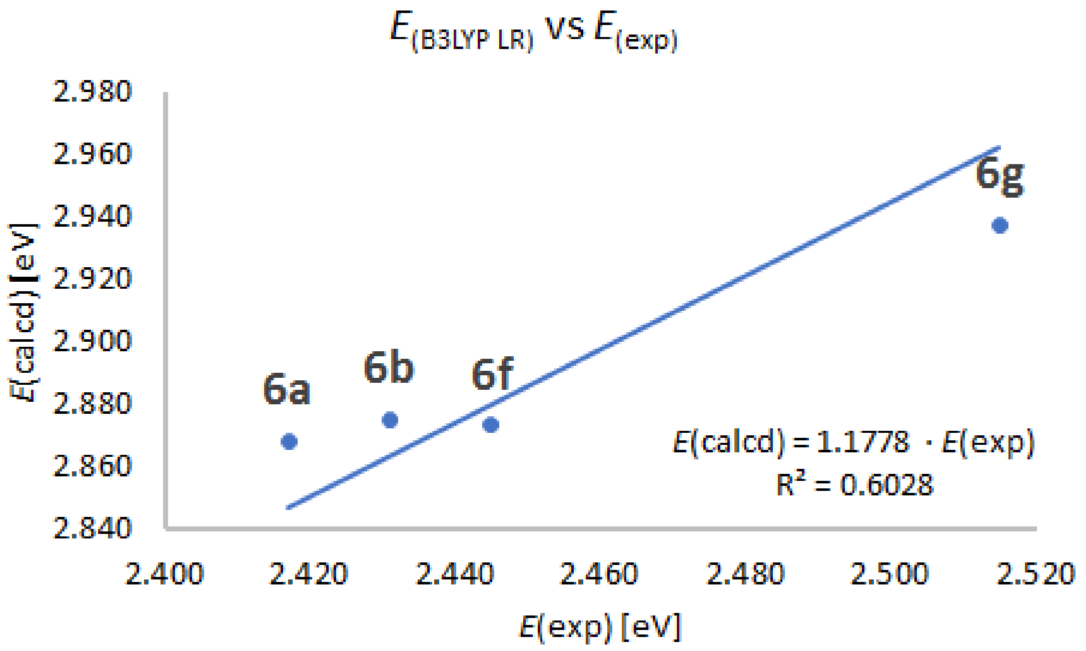

Figure S41. Plot of the calculated excitation energies (B3LYP LR) of merocyanines **6a**, **6b**, **6f**, and **6g** $E_{\text{(B3LYP LR)}}$ vs $E_{\text{(exp)}}$, linear regression.

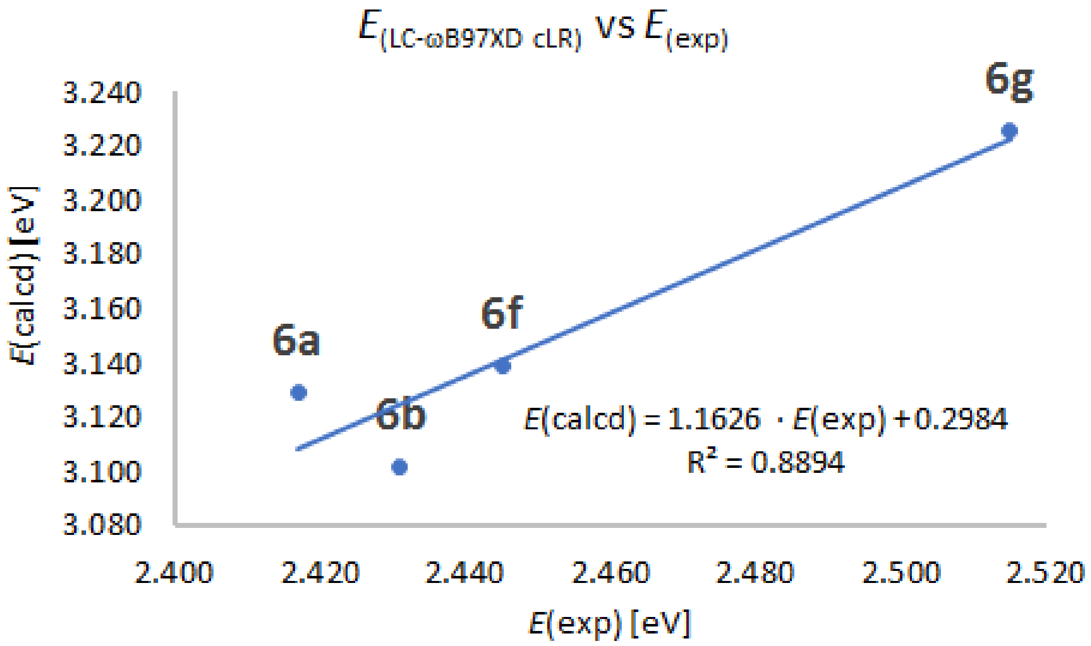

Figure S42. Plot of the calculated excitation energies (LC-ωB97XD cLR) of merocyanines **6a**, **6b**, **6f**, and **6g** $E_{(LC\text{-}\omega B97XD\ cLR)}$ vs $E_{(exp)}$, linear regression.

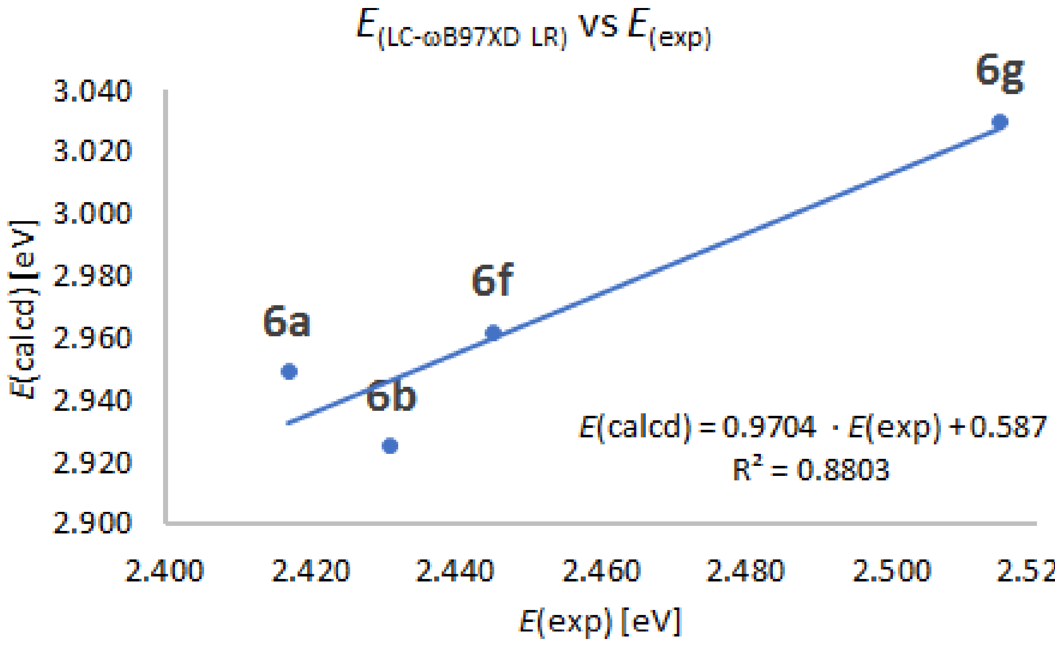

Figure S43. Plot of the calculated excitation energies (LC-ωB97XD LR) of merocyanines **6a**, **6b**, **6f**, and **6g** $E_{(LC\text{-}\omega B97XD\ LR)}$ vs $E_{(exp)}$, linear regression.

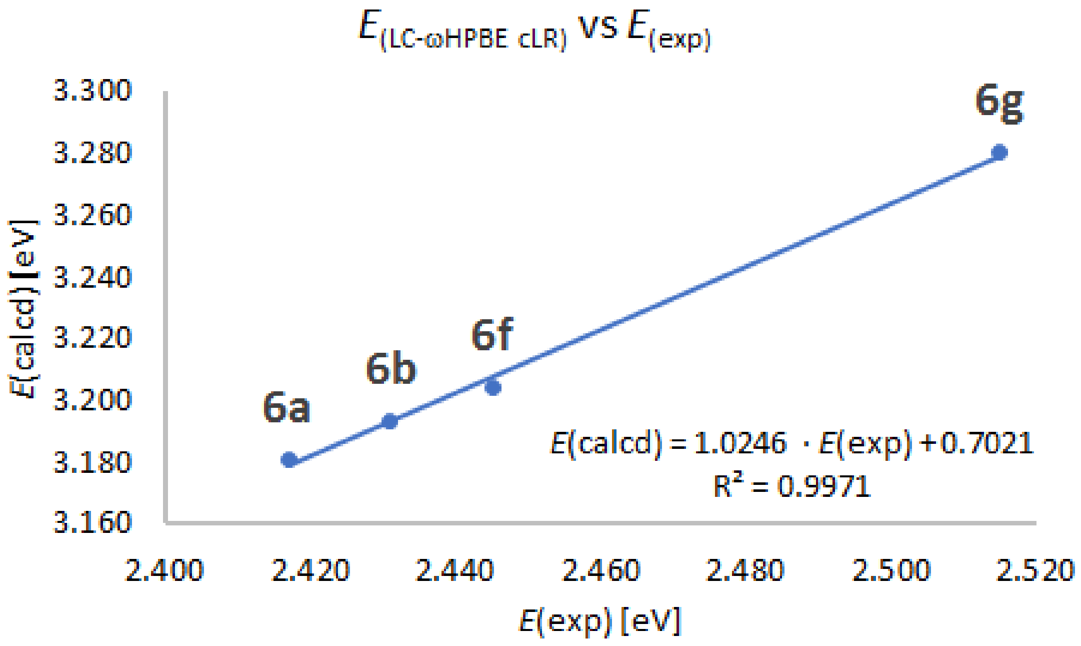

Figure S44. Plot of the calculated excitation energies (LC-ωHPBE cLR) of merocyanines **6a**, **6b**, **6f**, and **6g** $E_{\text{(LC-ωHPBE cLR)}}$ vs $E_{\text{(exp)}}$, linear regression.

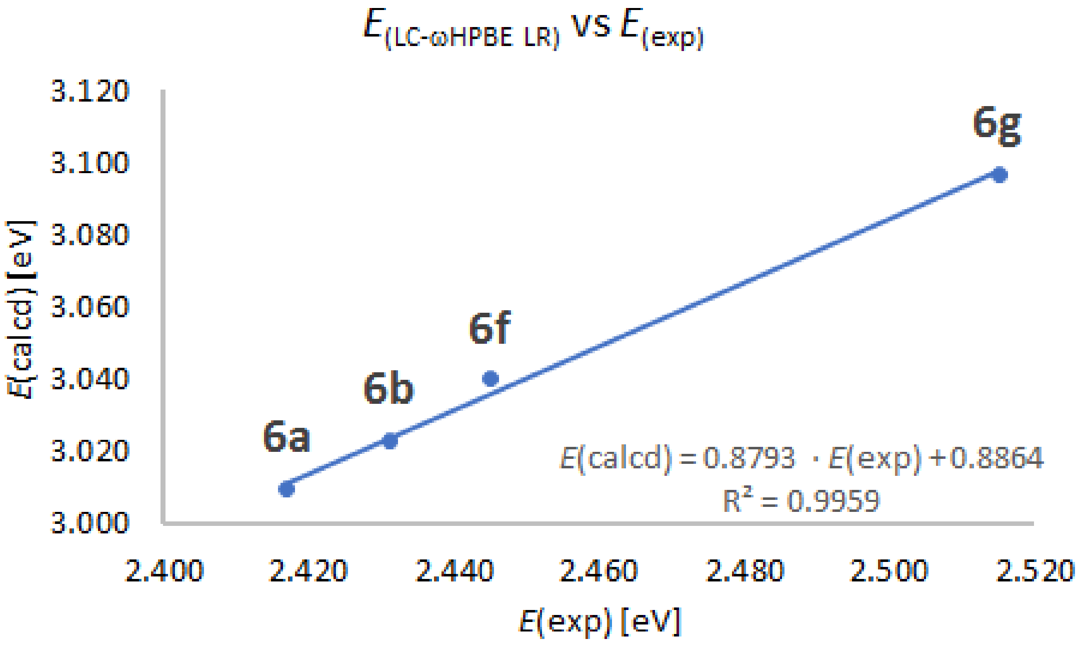

Figure S45. Plot of the calculated excitation energies (LC-ωHPBE LR) of merocyanines **6a**, **6b**, **6f**, and **6g** $E_{\text{(LC-ωHPBE LR)}}$ vs $E_{\text{(exp)}}$, linear regression.

10. References

[1] A. G. Cook, M. L. Absi, V. K. Bowden, *J. Org. Chem.* **1995**, *60*, 3169-3171.

[2] J. Papadopoulos, T. Gerlach, G. J. Reiss, B. Mayer, T. J. J. Müller, Consecutive multicomponent coupling-addition synthesis and chromophore characteristics of cyclohexene embedded merocyanines and cyanines. *Photochem* **2022**, *2*, 672-693. DOI: 10.3390/photochem2030044
